# Betulin–Amino Acid Molecular Hybrids: Synthesis, Structure and Pharmacological Potential

**DOI:** 10.3390/ijms27104445

**Published:** 2026-05-15

**Authors:** Mirosława Grymel, Paweł Naprawca, Daria Dolniak-Budny, Mateusz D. Tomczyk, Mateusz Pielok, Beata Nowrot, Klaudia Skutnik, Karol Erfurt, Anna Lalik

**Affiliations:** 1 Department of Organic Chemistry, Bioorganic Chemistry and Biotechnology, Silesian University of Technology, B. Krzywoustego 4, 44-100 Gliwice, Poland; pawelnaprawca@interia.pl (P.N.); daria.dolniak@gmail.com (D.D.-B.); mateusz.d.tomczyk@polsl.pl (M.D.T.); mp301255@student.polsl.pl (M.P.); 2 Biotechnology Center, Silesian University of Technology, B. Krzywoustego 8, 44-100 Gliwice, Poland; 3 Department of Systems Biology and Engineering, Silesian University of Technology, Akademicka 16, 44-100 Gliwice, Poland; beatnow835@student.polsl.pl (B.N.); ks308126@student.polsl.pl (K.S.); 4 Department of Chemical Organic Technology and Petrochemistry, Silesian University of Technology, B. Krzywoustego 4, 44-100 Gliwice, Poland; karol.erfurt@polsl.pl

**Keywords:** betulin, cytotoxicity, apoptosis, cell cycle, redox balance

## Abstract

The multidirectional bioactivity of betulin (BN), its widespread occurrence in plants, relatively low toxicity, and acceptable safety profile make it an attractive scaffold for scientific research and potential therapeutic applications. Due to the presence of reactive functional groups (C-3-OH and C-28-OH), BN is an interesting source of new semisynthetic bioactive compounds obtained via structural modifications of the parent backbone. In our study, we designed new BN–amino acid (BNAA) molecular hybrids, aiming to exploit synergistically the properties of both components. We prepared and evaluated a total of 18 new compounds for antitumor activity against the two human cancer cell lines (HCT 116 and MCF-7) and one non-cancerous cell line (NHDF) using a standard Cell Counting Kit-8 (CCK-8) assay. The potential signaling pathways of the obtained BN derivatives were identified based on the measurement of p21 and Bax mRNA expression levels using the RT-qPCR method. We successfully synthesized a series of new BN hybrids by conjugation of the C-3 and C-28 hydroxyl groups *via* a succinyl (-CO-CH_2_-CH_2_-CO-, Suc) linker with selected amino acid methyl esters. The structures of all obtained BNAA molecular hybrids were confirmed by spectroscopic analysis (^1^H and ^13^C NMR) and high-resolution mass spectrometry (HR-MS). Analysis of the biological activity of the obtained BN derivatives indicated that both the attached amino acids and the substituents at C3 carbon alter BN activity. The obtained BN–amino acid hybrids represent a useful platform for further optimization, especially derivatives (**3a**, **3e**, **3f**, and **7d**), which showed the most relevant biological profiles in this study.

## 1. Introduction

Advances in medical science, the development of diagnostic techniques and increasing public awareness make it possible to treat an ever-widening spectrum of diseases. Unfortunately, effective therapies are still lacking for many type of cancer, which constitute one of the leading causes of death worldwide in the 21st century, according to the World Health Organization (WHO) [1]. Cancer therapy is still associated with adverse reactions and drug resistance, despite decades of advancement in anticancer research and drug discovery [2,3,4,5,6,7,8]. Therefore, there is still a need for new anticancer compounds with improved selectivity, lower systemic toxicity, and activity against drug-resistant cancer cells.

A promising trend is the search for new bioactive molecules with potential anticancer activity among compounds of natural origin. Researchers’ attention focuses on 3-lup-20(29)-ene-3β,28-diol, one of the most popular and readily available lupane-type pentacyclic triterpenoids, commonly known as betulin (BN) [9,10,11]. BN shows a number of pharmacologically relevant properties, including cytotoxic, anti-inflammatory, antiviral (e.g., against HIV), antibacterial (e.g., against *Streptococcus mutans*), antifungal, antidiabetic and neuroprotective activities, which make it an attractive molecule for scientific research and potential therapeutic applications [9,12,13,14]. The multidirectional activity of BN, its high safety, acceptable side effects, and promising efficacy makes it a potential candidate for the treatment of many diseases, especially cancer, autoimmune, viral, neurodegenerative, and metabolic diseases. In vitro studies have repeatedly demonstrated that BN exhibits cytotoxic activity against many types of cancer cells, including colon carcinoma, colorectal adenocarcinoma, breast, lung, glioma, melanoma, prostate, liver, and osteosarcoma [13,15,16,17,18,19,20,21]. In addition, it exhibits synergistic effects with some anticancer drugs (such as doxorubicin) [22], which can increase the efficacy of chemotherapy and the sensitivity of cancer cells to radiotherapy [13,23]. Moreover, compared to some conventional anticancer drugs, BN appears to be less toxic to healthy cells [9,13].

Despite its multidirectional biological activity and relatively favorable safety profile, BN has poor drug-like properties, including low water solubility, high hydrophobicity, low bioavailability, short half-life, and insufficient intracellular accumulation. These limitations reduce its therapeutic potential in vivo and justify further structural modification of the BN scaffold [9,11,12,13].

**Figure 1:**
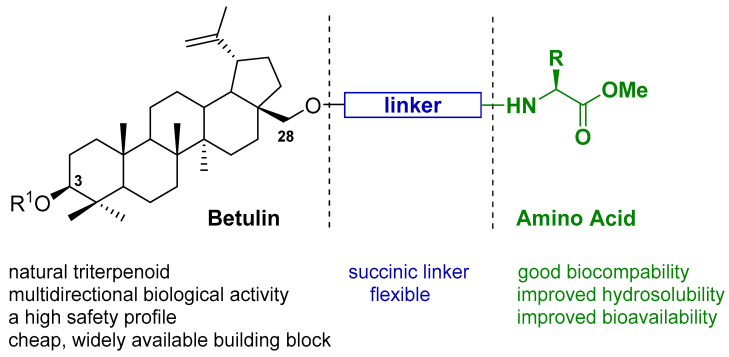
Structural modifications of the skeleton of BN *via* conjugation with amino acid methyl esters: (i) at the C-28 position (28-monosubstituted analog; R^1^ = Ac or H), (ii) at the C-3 and C-28 positions (3,28-disubstituted analog; R^1^ = (CO)CH_2_CH_2_CONHCH(R)COOMe).

In our research, we designed new BN–amino acid molecular hybrids (BNAA), aiming to combine the pharmacological potential of BN with the ability of amino acid residues to modulate polarity, solubility, membrane interactions, and cellular uptake (Figure 1).

Amino acids can increase the permeation of the hybrid across cell membranes, which is crucial for their intracellular efficacy (including in mitochondria), can participate in the binding of the hybrid to target enzymes, and can also be used to improve the stability of the drug in the body, protecting it from enzymatic or metabolic degradation [9,13,23]. In addition, the scientific literature mentions conjugates of BN or betulinic acid (BA) with amino acids in the context of improving water solubility and increasing antitumor activity (Table 1) [24,25,26,27,28].

**Figure 2:**
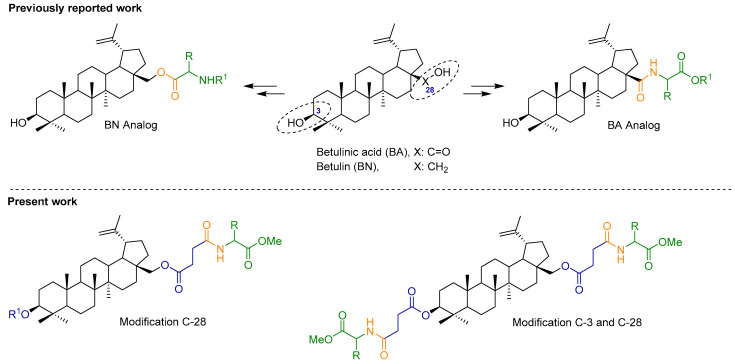
Molecular hybrids of triterpenoids (BN, BA) with amino acids (AA).

Several research groups have previously described a series of compounds, in which the amino acid moiety is attached to the precursor backbone (BN or BA) *via* an ester bond (in BN analogs) or amide bond (in BA analogs) as shown in Figure 2. Both BN and BA are widely distributed in the plant kingdom, especially in the outer layer of birch bark (*Betulaceae*, *Betula*, *Betula pendula*). However, BN is considerably more available, as its content can reach 34%, while BA accounts for only 0.3% of the dry weight of the extract [11]. This is a clear advantage of BN, especially in the context of large-scale drug production.

In this study, we proposed new BN derivatives (Figure 2), considering that the type of bond connecting the BN skeleton with the amino acid may be an important factor influencing the hydrolysis process inside the cell. The type of bond determines the involvement of various enzymes, including esterases or amidases.

We designed BN derivatives in which the amino acid moiety was attached to the precursor skeleton *via* a linker terminated with a carboxyl group (-CO-CH_2_-CH_2_-COOH), enabling the formation of an amide bond (similarly to BA analogs). Moreover, the presence of the succinyl linker should increase the flexibility of the entire molecule and, as a result, facilitate its fit to a potential molecular target.

The use of natural amino acids as modifiers of the BN structure may reduce the risk of toxicity and side effects compared to synthetic modifiers [19]. Amino acid selection can affect lipophilicity, polarity, stability, and interactions of connections to proteins and cell membranes [24,26]. However, it should be remembered that a compromise is necessary between cell membranes permeability and the efficiency of interactions with molecular targets inside cells.

Unfortunately, it is not possible to clearly indicate “the best amino acid”, because different amino acids may influence different aspects of the action of the BNAA molecular hybrid. The presence of hydrophilic, polar, low molecular weight glycine in the BNAA structure theoretically should increase solubility in water, which is beneficial for the bioavailability of the drug. Likewise, non-polar amino acids of hydrophobic nature (e.g., alanine, valine, leucine, tryptophan) have a significant influence on cell membranes penetration and may be useful in modifying the BN skeleton by increasing its lipophilicity. However, it should be remembered that excessive lipophilicity may lead to reduced solubility and a lack of specific interactions with the molecular target [17,23]. On the other hand, polar amino acids (e.g., serine, threonine, tyrosine) may potentially increase the solubility of BNAA, which would be beneficial for its bioavailability [24]. In turn, hydrophobic tryptophan is a relatively inflexible amino acid, which may confer specificity to the hybrid’s interaction with target proteins. The thioether group present in the methionine side chain, due to its ability to reduce peroxides, may potentially act as an antioxidant. The relatively small molecular weight of polar serine compared to that of, e.g., tryptophan may result in better distribution of the drug in the body compared to larger amino acids. Due to its cyclic structure, proline is more resistant to enzymatic degradation, which could improve the stability of the BNAA in a biological environment. On the other hand, its rigid structure may hinder the alignment of BNAA with the active site of the target protein [17,23].

In each case, the designed and then synthesized molecular hybrid required confirmation of the structure, analysis of physicochemical properties and assessment of biological activity.

## 2. Results and Discussion

### 2.1. Chemistry

**Scheme 1:**
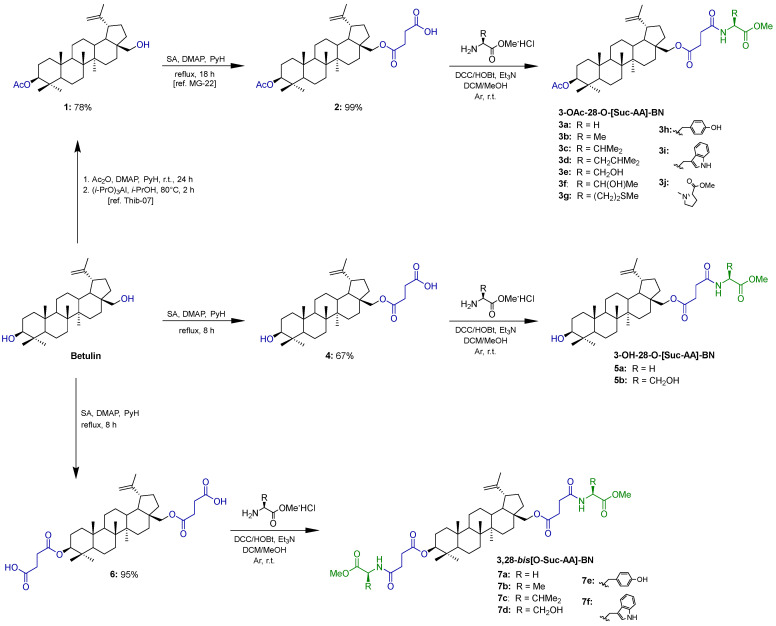
Synthetic route for the preparation of BNAA hybrids (**3a**–**j**, **5a**–**b** and **7a**–**f**).

As a continuation of our previous studies [29,30,31] we synthesized a series of new semisynthetic BN derivatives. In order to obtain a library of compounds intended for the assessment biological activity, structural modifications of the BN skeleton were carried out by introducing one molecule amino acid methyl ester moiety at the C-28 position (monosubstituted analogs: 3-OAc-28-*O*-[Suc-AA]-BN and 3-OH-28-*O*-[Suc-AA]-BN) or two amino acid moieties at the C-3 and C-28 positions (disubstituted analogs: 3,28-*bis*[*O*-Suc-AA]-BN) as shown in Scheme 1.

Research on the synthesis of BNAA hybrids began with the synthesis of 3-*O*-acetylbetulin **1**, 3-*O*-acetyl-28-*O*’-(3′-carboxypropanoyl)betulin **2**, and 3,28-*O*,*O*′-*bis*(3′-carboxypropanoyl)betulin **6** based on procedures described in the literature [30,32], with some modifications.

In this study, we aimed to evaluate whether the free hydroxyl group at the C-3 position of the BN skeleton could influence the biological activity of BN derivatives. To obtain 28-*O*’-(3′-carboxypropanoyl)betulin **4**, the precursor (BN) was esterified with succinic anhydride (SA) in dry pyridine (Py) in the presence of 4-(dimethylamino)pyridine (DMAP), with the reaction carried out under reflux for 8 h. The expected product **4** was obtained with a good yield of 67%. The selective esterification of the primary hydroxyl group (C-28-OH ), while maintaining the free secondary group (C-3-OH), was possible by reducing the excess of succinic anhydride (BN:SA molar ratio, 1:1.5) and carefully controlling the reaction progress (^1^H NMR).

In the next step, BNAA hybrids (**3**, **5**, and **7**) were obtained starting from commercially available L-amino acid methyl ester (glycine, alanine, valine, leucine, tryptophan, serine, threonine, tyrosine, methionine, proline) and the appropriate (carboxyacyl)betulin derivatives (**2**, **4**, **6**). The use of amino acids in the form of esters enabled a reaction between the carboxyl group of the linker -CO-CH_2_-CH_2_-COOH and the amino terminus of the amino acid, allowing the formation of specific combinations *via* the amide bond.

3-*O*-Acetyl-28-*O*’-(3′-carboxypropanoyl)betulin **2** or 28-*O*’-(3′-carboxypropanoyl) betulin **4** was coupled with amino acid methyl ester hydrochloride (AA) using *N*,*N*’-dicyclohexylcarbodiimide (DCC) and 1-hydroxybenzotriazole as coupling agents in the presence of triethylamine (Et_3_N) in a molar ratio of 1.0 mmol (**2** or **4**)/1.5 mmol DCC/1.0 mmol HOBt/1.5 mmol AA/1.5 mmol Et_3_N, at room temperature for 2–5 days. The first experiment was performed by generating an active ester. Method A consisted of mixing the BN analog with DCC/HOBt for 1 h at room temperature under argon, and then adding a solution of amino acid methyl ester and Et_3_N in dry DCM. Then, to simplify the methodology, the active ester generation step was eliminated. In one flask, the BN analog (**2** or **4**), the coupling agents DCC/HOBt, and H_2_NCH(R)COOMe·HCl in dry DCM were mixed at the same molar ratio. Et_3_N was added dropwise after 1 h (Method B).

The synthesis of 3,28-*bis*[O-Suc-AA]-BN **7**, in turn, was performed using the same procedure (Method B), starting from *3*,28-*O*,*O*’-*bis*(3′-carboxypropanoyl)betulin **6**. The reagents were used in a molar ratio of 1.0 mmol **6**/2.5 mmol DCC/2.0 mmol HOBt/2.5 mmol AA/2.5 mmol Et_3_N. In each case, the reaction was carried out under an argon atmosphere at room temperature for 2–5 days. A crucial factor was the selection of an appropriate reaction medium, ensuring contact between all reactants. High conversion of the substrates (**2**, **4**, **6**) was achieved using a DCM/MeOH solvent system.

The final products (**3**, **5,** and **7**) were isolated by extraction with ethyl acetate and subsequent purification by column chromatography to afford the products in satisfactory yields (Table 2). The structures of all synthesized compounds (**3**–**7**) were confirmed by spectroscopic methods (^1^H, ^13^C NMR) and high-resolution mass spectrometry (HRMS), and are available in Supplementary Materials.

### 2.2. Biology

BN can both induce apoptosis and protect cells by blocking the production of reactive oxygen species (ROS) and upregulating antioxidant enzymes from the Nrf2/SOD pathway [33,34]. Amino acids perform a building function in cells, but also play an important role in maintaining redox balance and energy regulation [35]. Amino acids are also a key factor influencing cell cycle length and proliferation [36]. The amino acids attached to BN may therefore not only increase the bioavailability of BN but also modulate its activity. To verify this statement, the biological activity of BNAA hybrids was evaluated using the CCK-8 assay. Since it is suggested that BN and its derivatives exhibit selective toxicity [33], the studies were conducted on two cancer cell lines (HCT 116, MCF-7) and on one normal cell line (NHDF).

**Figure 3:**
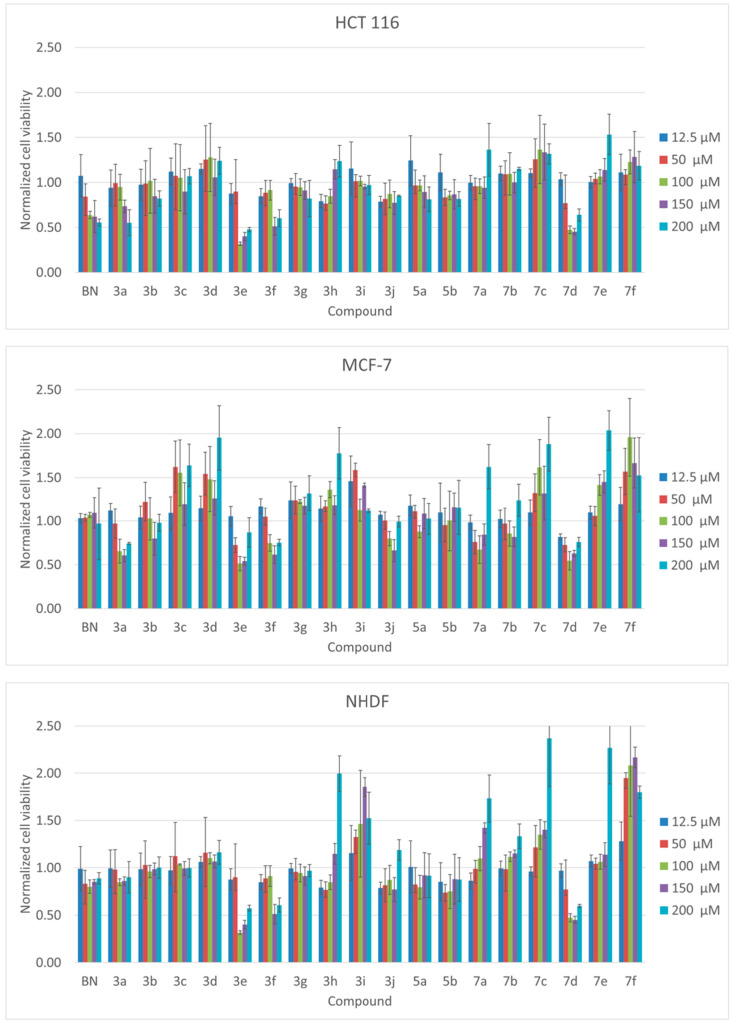
Effect of the tested compounds on the cell viability of HCT 116, MCF-7 and NHDF cells. Cells were treated with different concentrations of BNAA hybrids for 24 h and then cell viability was measured by the CCK-8 assay. The results are provided as mean values with ±SD from at least three independent experiments. Cell viability was normalized to control (untreated) cells.

The results indicated that the biological activity of the BNAA hybrids tested depends on the attached amino acid, the cell type, and the substitution pattern of the BN scaffold (Figure 3). The concentration–response profiles for the tested compounds were non-monotonic and did not allow for a reliable fit to a standard sigmoidal dose–response model therefore, IC_50_ values were not calculated for them (a detailed explanation can be found in the Supplementary Materials in Figure S39). However, statistical analysis of the obtained data showed that the effect of the tested BNAAs on cells is concentration-dependent (Supplementary Materials, Table S1). Importantly, C-28 monosubstitution was sufficient to obtain active derivatives, as shown for **3a**, whereas C-3/C-28 disubstitution did not uniformly improve biological activity.

BNAA hybrid **3a** showed the greatest pharmacological potential, exhibiting toxic effects on cancer cells (HCT 116, MCF-7) at higher concentrations, while having no effect on the viability of normal NHDF cells. **3a** is a hybrid of BN and glycine, so the selective action of this derivative may be the result of **3a** binding to the specific glycine transporter (GlyT1), which may lead to the blocking of GlyT1 activity. Glycine is used in cells to synthesize glutathione (GSH), a key antioxidant found in mammalian cells [37]. Increased ROS production in cancer cells causes an increased demand for GSH. Cancer cells are therefore characterized by overexpression of GlyT1 [38]. Inhibition of GlyT1 leads to a decrease in glycine uptake, which causes a redox imbalance and inhibition of cell proliferation [37]. Normal cells have lower levels of GlyT1 expression and lower levels of intracellular ROS than cancer cells, so blocking GlyT1 activity may have less impact on their viability.

Serine in cells participates in maintaining redox balance and supports GSH synthesis and cell proliferation [39]. Surprisingly, in our studies, betulin derivatives with attached serine (**3e**, **7d**) showed the strongest cytotoxic effect (Figure 3). Analysis of the mechanism of action of serine suggests that BNAA (**3e** and **7d**), like D-serine, may compete with L-serine in binding to the mitochondrial transporter of L-serine, reducing the pool of available substrate for serine hydroxymethyltransferase 2 (SHMT2). This leads to inhibition of one-carbon metabolism and induction of ROS-dependent mitochondrial-mediated apoptosis [40,41].

Interestingly, the addition of tryptophan to BN skeleton (**3i**, **7f**) increased the viability of MCF-7 and NHDF cells (Figure 3). The stimulating effect of BNAA (**3i** and **7f**) may result from the properties of tryptophan. Tryptophan participates in maintaining redox homeostasis in cells and is involved in protein synthesis [42,43]. Tryptophan may also protect proteins from oxidative damage, which may affect the biological activity of cells [44]. It is worth noting that an increase in tryptophan concentration in cells has been linked to the inhibition of apoptosis [45], which may explain the increase in cell viability observed in our studies of cells treated with derivatives **3i** and **7f** (Figure 3).

Studies conducted on cells treated with BN and its derivatives have shown that the cytotoxic effect of BN results mainly from the induction of the mitochondrial apoptosis pathway and cell cycle arrest [46]. To verify the correlation among apoptosis, cell cycle, and reduced cell viability, we determined the expression levels of mRNAs encoding key proteins regulating apoptosis and the cell cycle (p21, Bax). The studies were conducted on HCT 116 cells treated with 100 µM BNAA hybrids exhibiting the highest cytotoxicity (**3e**, **3f**, **7d**), BN, or a BN analog (**3d**) that did not significantly alter cell viability.

Bax is one of the core regulators of the mitochondrial pathway of apoptosis [47]. The key protein regulating cell cycle arrest is p21, an inhibitor of cyclin-dependent kinases. Depending on its cellular location, p21 can also regulate apoptosis, senescence, and gene transcription [48]. Overexpression of p21 inhibits cancer cell proliferation and regulates oxidative stress [49,50]. Thus, both p21 and Bax are important markers of the cellular response to anticancer treatment.

**Figure 4:**
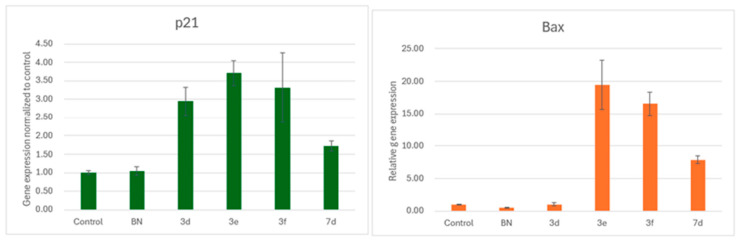
The relative expression levels of p21 and Bax in the HCT 116 cells treated with BNAA hybrids. Cells were treated with 100 µM BNAA hybrids for 24 h, after which the expression levels of p21 and Bax were measured using RT-qPCR. Relative quantification of the results was performed using the ΔΔCt method. Data is presented as the means of triplicate measurements ± SD.

RT-qPCR studies showed that all analyzed BNAA hybrids (**3d**–**f** and **7d**), unlike BN, induce p21 expression, but only in cells treated with BNAA (**3e, 3f,** and **7d**) does the level of Bax mRNA increase (Figure 4). The results obtained, combined with the cell viability test (Figure 3), suggest that derivative **3d** inhibits the cell cycle, and that the toxic effect observed for derivatives (**3e**, **3f**, and **7d**) is associated with the induction of intrinsic apoptosis pathway.

## 3. Materials and Methods

### 3.1. Synthesis and Analysis

#### 3.1.1. General Information

Nuclear magnetic resonance spectra (NMR) were recorded on a Varian spectrometer (Palo Alto, CA, USA) at 600 and 150 MHz using tetramethylsilane (TMS) as the resonance shift standard (^1^H NMR, zero ppm) or residual chloroform (^13^C NMR, 77.16 ppm). All chemical shifts (δ) were reported in ppm, and coupling constants (*J*) in Hz. Multiplicities of the signals are abbreviated as singlet (s), doublet (d), double triplet (dt), triplet (t), quartet (q), multiplet (m) and broad (br). High-Resolution Mass Spectrometry (HRMS) analyses were performed using a Waters Xevo G2 Q-TOF mass spectrometer (Waters Corporation, Milford, MA, USA) equipped with an ESI source operating in positive-ion mode. The accurate mass and composition for the molecular ion adducts were calculated using MassLynx software v.4.1 (Waters Corporation, Milford, MA, USA) incorporated in the instrument. Optical rotation values were measured with a P-2200 polarimeter (JASCO Corporation, Tokyo, Japan) equipped with a sodium lamp (589.3 nm) at room temperature. Reaction progress was monitored by thin-layer chromatography (TLC) analysis using silica gel 60 F254 plates (Merck, Darmstadt, Germany) for R_f_ values; plates were visualized by UV light (λ = 254 nm) or by charring after spraying with a solution of cerium ammonium molybdate in sulfuric acid.

All chemical reagents and solvents used in the synthesis were purchased from Acros Organics (Geel, Belgium) or Avantor (Gliwice, Poland) and used without further purification. 3-*O*-Acetylbetulin **1**, 3-*O*-acetyl-28-*O*’-(3′-carboxypropanoyl)betulin **2**, and 3,28-*O*,*O*′-bis(3′-carboxypropanoyl)betulin **6** were prepared according to the respective published procedures [30,32].

#### 3.1.2. General Procedure for the Synthesis of 3-OAc-28-O-[Suc-AA]-BN Hybrids (**3a**–**j**)

Method A: 3-*O*-acetyl-28-*O*’-(3′-carboxypropanoyl)betulin (**2**, 0.20 mmol, 117.0 mg), DCC (0.30 mmol, 61.9 mg, 1.5 eq.) and HOBt (0.20 mmol, 30.6 mg, 1 eq.) was dissolved in dry DCM (3–7 mL). The heterogenous reaction mixture was stirred for 30 min at 5 °C under argon atmosphere and then for 1 h at room temperature. Next, a solution of the appropriate amino acid methyl ester hydrochloride (H_2_NCH(R)COOMe·HCl (0.30 mmol, 1.5 eq.) and Et_3_N (0.30 mmol, 30.4 mg, 0.04 mL, 1.5 eq.) in DCM (2–7 mL) was added dropwise. Then, methanol was added in an amount sufficient to completely dissolve all the reagents, and the reaction was carried out at room temperature for 2–5 days. After completion of the reaction (monitored on ^1^H NMR or TLC), the mixture was concentrated in vacuo, and the residue was extracted with ethyl acetate (AcOEt). The crude products (**3**) were purified by column chromatography.

Method B: 3-*O*-acetyl-28-*O*’-(3′-carboxypropanoyl)betulin (**2**, 0.20 mmol, 117.0 mg), DCC (0.30 mmol, 61.9 mg, 1.5 eq.), HOBt (0.20 mmol, 30.6 mg, 1 eq.) and H_2_NCH(R)COOMe·HCl (0.30 mmol, 1.5 eq.) were dissolved in dry DCM (5–14 mL) and stirred vigorous for 1 h at room temperature under argon atmosphere. Then, Et_3_N (0.30 mmol, 1.5 eq.) and methanol were added dropwise. The reaction was carried out at room temperature for 2–5 days. After completion of the reaction (monitored on ^1^H NMR or TLC), the expected products **3** were isolated and purified as in Method A.

##### 3-OAc-28-*O*-[Suc-Gly(OMe)]-BN (**3a**)

Viscous oil (75% yield, Method A); R_f_ = 0.29 (DCM/MeOH, 50:1); CC (DCM/MeOH, 50/1); HRMS (ESI^+^): *m*/*z*: calcd for C_39_H_61_NO_7_Na [M+Na]^+^ 678.4346, found 678.4346; [α]^25^_D_ = 10.3 (c = 1, CHCl_3_); ^1^H NMR (600 MHz, CDCl_3_): δ_H_ 0.83, 0.84, 0.96, 1.02 (all s, 15H, CH_3_-23–CH_3_-27), 1.68 (s, 3H, CH_3_-30), 0.72–2.01 (m, 24H, CH, CH_2_, BN scaffold), 2.04 (s, 3H, CH_3_CO), 2.43 (td, 1H, *J*_1_ = 5.4 Hz, *J*_2_ = 10.8 Hz, H-19), 2.56–2.72 (m, 4H, O(CO)CH_2_CH_2_), 3.76 (s, 3H, OCH_3_), 3.88 (d, 1H, *J* = 10.8 Hz, H-28b), 4.05 (d, 2H, *J* = 5.4 Hz, NHC*H*_2_), 4.28 (d, 1H, J = 10.8 Hz, H-28a), 4.47 (dd, 1H, J_1_ = 5.4 Hz, *J*_2_ = 10.8 Hz, H-3), 4.59 (s, br, 1H, H-29b), 4.68 (s, br, 1H, H-29a), 6.23 (s, br, 1H, NH) ppm; ^13^C NMR (150 MHz, CDCl_3_): δ_C_ 14.84 (C-27), 16.15 (C-24), 16.27 (C-26), 16.61 (C-25), 18.28 (C-6), 19.23 (C-30), 20.91 (C-11), 21.43 (*C*H_3_CO), 23.81 (C-2), 25.27 (C-12), 27.16 (C-15), 28.06 (C-23), 29.63 (C-16), 30.90 (C-21), 29.68 and 29.84 ((CO)*C*H_2_*C*H_2_), 34.23 (C-7), 34.63 (C-22), 37.18 (C-10), 37.70 (C-13), 37.91 (C-4), 38.50 (C-1), 41.01 (C-8), 41.42 (NHC), 42.81 (C-14), 46.52 (C-17), 47.84 (C-19), 48.91 (C-18), 50.39 (C-9), 52.49 (OCH_3_), 55.49 (C-5), 63.23 (C-28), 81.05 (C-3), 110.00 (C-29), 150.22 (C-20), 170.47, 171.14, 171.67, 173.32 (4 × CO) ppm.

##### 3-OAc-28-*O*-[Suc-Ala(OMe)]-BN (**3b**)

Viscous oil (68% yield, Method A); R_f_ = 0.53 (DCM/MeOH, 50:1); CC (DCM/MeOH, 50:1); HRMS (ESI^+^): *m*/*z*: calcd for C_40_H_64_NO_7_ [M+H]^+^ 670.4683, found 670.4685; [α]^25^_D_ = 6.0 (c = 1, CHCl_3_); ^1^H NMR (600 MHz, CDCl_3_): δ_H_ 0.83, 0.84, 0.96, 1.02 (all s, 15H, CH_3_-23–CH_3_-27), 1.40 (d, 3H, *J* = 7.2 Hz, CHC*H*_3_), 1.68 (s, 3H, CH_3_-30), 0.75–2.01 (m, 27H, CH, CH_2_, BN scaffold and CHC*H*_3_), 2.04 (s, 3H, CH_3_CO), 2.43 (td, 1H, *J*_1_ = 5.4 Hz, *J*_2_ = 11.4 Hz, H-19), 2.52–2.74 (m, 4H, O(CO)CH_2_CH_2_), 3.75 (s, 3H, OCH_3_), 3.88 (d, 1H, *J* = 10.8 Hz, H-28b), 4.28 (d, 1H, *J* = 10.8 Hz, H-28a), 4.47 (dd, 1H, *J*_1_ = 5.4 Hz, J_2_ = 10.8 Hz, H-3), 4.56–4.61 (m, 2H, H-29b, NHC*H*), 4.68 (s, br, 1H, H-29a), 6.21 (s, br, 1H, NH) ppm; ^13^C NMR (150 MHz, CDCl_3_): δ_C_ 14.85 (C-27), 16.15 (C-24), 16.28 (C-26), 16.62 (C-25), 18.29 (C-6), 18.62 (CH_3_), 19.24 (C-30), 20.92 (C-11), 21.45 (*C*H_3_CO), 23.82 (C-2), 25.28 (C-12), 27.17 (C-15), 28.07 (C-23), 29.62 (C-16), 31.03 (C-21), 29.70 and 29.85 ((CO)*C*H_2_*C*H_2_), 34.24 (C-7), 34.65 (C-22), 37.19 (C-10), 37.71 (C-13), 37.92 (C-4), 38.51 (C-1), 41.02 (C-8), 42.82 (C-14), 46.54 (C-17), 47.84 (C-19), 48.21 (NHC), 48.91 (C-18), 50.40 (C-9), 52.59 (OCH_3_), 55.50 (C-5), 63.18 (C-28), 81.05 (C-3), 110.01 (C-29), 150.25 (C-20), 170.99, 171.15, 173.31, 173.61 (4 × CO) ppm.

##### 3-OAc-28-*O*-[Suc-Val(OMe)]-BN (**3c**)

Viscous oil (92% yield, Method A); R_f_ = 0.47 (DCM/MeOH, 50:1); CC (DCM/MeOH, 50:1); HRMS (ESI^+^): *m*/*z*: calcd for C_42_H_68_NO_7_ [M+H]^+^ 698.4996, found 698.5005; [α]^25^_D_ = 13.3 (c = 1, CHCl_3_); ^1^H NMR (400 MHz, CDCl_3_): δ_H_ 0.83, 0.84, 0.96, 1.02 (all s, 15H, CH_3_-23–CH_3_-27), 0.91 (d, 3H, *J* = 7.2 Hz, CHC*H*_3_), 0.94 (d, 3H, *J* = 6.6 Hz, CHC*H*_3_), 1.23 (sep, 1H, *J* = 13.2 Hz, C*H*(CH_3_)_2_), 1.68 (s, 3H, CH_3_-30), 0.76–2.05 (m, 30H, CH, CH_2_, BN scaffold), 2.04 (s, 3H, CH_3_CO), 2.14-2.17 (m, 1H, NHCHC*H*), 2.42 (td, 1H, *J*_1_ = 6.0 Hz, J_2_ = 11.4 Hz, H-19), 2.54–2.75 (m, 4H, O(CO)CH_2_CH_2_), 3.74 (s, 3H, OCH_3_), 3.89 (d, 1H, *J* = 10.8 Hz, H-28b), 4.26 (d, 1H, *J* = 11.4 Hz, H-28a), 4.46 (dd, 1H, *J*_1_ = 5.4 Hz, *J*_2_ = 10.2 Hz, H-3), 4.55 (dd, 1H, *J*_1_ = 4.8 Hz, *J*_2_ = 8.4 Hz NHC*H*), 4.58 (s, br, 1H, H-29b), 4.68 (s, br, 1H, H-29a), 6.15 (s, br, 1H, NH) ppm. ^13^C NMR (150 MHz, CDCl_3_): δ_C_ 14.87 (C-27), 16.17 (C-24), 16.31 (C-26), 16.64 (C-25), 18.32 (C-6), 19.26 (C-30), 17.95 and 19.07 (CH(*C*H_3_)_2_), 20.94 (C-11), 21.47 (*C*H_3_CO), 23.85 (C-2), 25.30 (C-12), 27.19 (C-15), 28.09 (C-23), 29.71 (C-16), 29.78 and 29.88 ((CO)*C*H_2_*C*H_2_), 31.23 (*C*H(CH_3_)_2_), 31.46 (C-21), 34.27 (C-7), 34.67 (C-22), 37.21 (C-10), 37.73 (C-13), 37.95 (C-4), 38.53 (C-1), 41.04 (C-8), 42.85 (C-14), 46.55 (C-17), 47.86 (C-19), 48.94 (C-18), 50.42 (C-9), 52.28 (OCH_3_), 55.52 (C-5), 57.23 (NHC), 63.24 (C-28), 81.07 (C-3), 110.02 (C-29), 150.28 (C-20), 171.16, 171.44, 172.62, 173.39 (4 × CO) ppm.

##### 3-OAc-28-*O*-[Suc-Leu(OMe)]-BN (**3d**)

Viscous oil (46% yield, Method B); R_f_ = 0.43 (DCM/MeOH, 50:1); CC (DCM/MeOH, 50:1); HRMS (ESI^+^): *m*/*z*: calcd for C_43_H_69_NO_7_Na [M+Na]^+^ 734.4972, found 734.4951; [α]^25^_D_ = −2.1, (c = 0.8, CHCl_3_); ^1^H NMR (600 MHz, CDCl_3_): δ_H_ 0.83, 0.84, 0.96, 1.02 (all s, 15H, CH_3_-23–CH_3_-27), 0.94 (dd, 6H, *J*_1_ = 1.2 Hz, *J*_2_ = 6.0 Hz, CH(C*H*_3_*)*_2_), 1.23 (sep, 1H, *J* = 13.2 Hz, C*H*(CH_3_)_2_), 1.40 (d, 2H, *J* = 10.8 Hz, C*H*_2_CH), 1.68 (s, 3H, CH_3_-30), 0.74–2.07 (m, 32H, CH, CH_2_, BN scaffold and CH_2_CH(CH_3_)_2_), 2.04 (s, 3H, CH_3_CO), 2.43 (td, 1H, *J*_1_ = 6.0 Hz, *J*_2_ = 11.4 Hz, H-19), 2.52–2.74 (m, 4H, O(CO)CH_2_CH_2_), 3.73 (s, 3H, OCH_3_), 3.89 (d, 1H, *J* = 10.8 Hz, H-28b), 4.27 (d, 1H, *J* = 10.8 Hz, H-28a), 4.47 (dd, 1H, *J*_1_ = 6.0 Hz, *J*_2_ = 10.8 Hz, H-3), 4.59 (s, br, 1H, H-29b), 4.60–4.64 (m, 1H, NHC*H*), 4.68 (s, br, 1H, H-29a), 6.11 (s, br, 1H, NH) ppm; ^13^C NMR (150 MHz, CDCl_3_): δ_C_ 14.84 (C-27), 16.13 (C-24), 16.27 (C-26), 16.60 (C-25), 18.27 (C-6), 19.23 (C-30), 20.91 (C-11), 21.43 (*C*H_3_CO), 22.12 and 22.89 (CH(*C*H_3_)_2_), 23.81 (C-2), 24.95 (*C*H(CH_3_)_2_), 25.27 (C-12), 27.15 (C-15), 28.05 (C-23), 29.65 (C-16), 29.69 and 29.84 ((CO)*C*H_2_*C*H_2_), 31.04 (C-21), 34.22 (C-7), 34.63 (C-22), 37.17 (C-10), 37.69 (C-13), 37.90 (C-4), 38.49 (C-1), 41.00 (C-8), 41.82 (*C*H_2_CH), 42.80 (C-14), 46.53 (C-17), 47.82 (C-19), 48.90 (C-18), 50.38 (C-9), 50.87 (NHC), 52.37 (OCH_3_), 55.48 (C-5), 63.16 (C-28), 81.03 (C-3), 109.98 (C-29), 150.20 (C-20), 171.11, 171.28, 173.32, 173.61 (4 × CO) ppm.

##### 3-OAc-28-*O*-[Suc-Ser(OMe)]-BN (**3e**)

Viscous oil (70% yield, Method B); R_f_ = 0.88 (DCM/MeOH, 1:20); CC (DCM/MeOH, 100:1); HRMS (ESI^+^): *m*/*z*: calcd for C_40_H_63_NO_8_Na [M+Na]^+^ 708.4551, found 708.4556; [α]^25^_D_ = 3.3, (c = 1, CHCl_3_); ^1^H NMR (600 MHz, CDCl_3_): δ_H_ 0.83, 0.84, 0.96, 1.02 (all s, 15H, CH_3_-23–CH_3_-27), 1.68 (s, 3H, CH_3_-30), 0.73–1.98 (m, 24H, CH, CH_2_, BN scaffold), 2.04 (s, 3H, CH_3_CO), 2.42 (td, 1H, *J*_1_ = 5.4 Hz, *J*_2_ = 10.8 Hz, H-19), 2.52–2.82 (m, 4H, O(CO)CH_2_CH_2_), 2.91 (s, br, 1H, OH), 3.80 (s, 3H, OCH_3_), 3.87 (d, 1H, *J* = 10.8 Hz, H-28b), 3.94–3.97 (m, 2H, C*H*_2_OH), 4.29 (dd, 1H, *J*_1_ = 1.2 Hz, *J*_2_ = 10.8 Hz, H-28a), 4.46 (dd, 1H, *J*_1_ = 5.4 Hz, *J*_2_ = 10.8 Hz, H-3), 4.59 (s, br, 1H, H-29b), 4.65 (dt, 1H, *J*_1_ = 7.8 Hz, *J*_2_ = 3.6 Hz, NHC*H*), 4.68 (s, br, 1H, H-29a), 6.64 (d, 1H, *J* = 7.2 Hz, NH) ppm; ^13^C NMR (150 MHz, CDCl_3_): δ_C_ 14.85 (C-27), 16.14 (C-24), 16.28 (C-26), 16.61 (C-25), 18.29 (C-6), 19.24 (C-30), 20.91 (C-11), 21.45 (*C*H_3_CO), 23.81 (C-2), 25.27 (C-12), 27.16 (C-15), 28.07 (C-23), 29.68 (C-16), 31.06 (C-21), 29.68 and 29.84 ((CO)*C*H_2_*C*H_2_(CO)), 34.24 (C-7), 34.64 (C-22), 37.18 (C-10), 37.71 (C-13), 37.92 (C-4), 38.50 (C-1), 41.01 (C-8), 42.81 (C-14), 46.53 (C-17), 47.82 (C-19), 48.90 (C-18), 50.39 (C-9), 52.89 (OCH_3_), 55.05 (NHC), 55.49 (C-5), 63.11 (C-28), 63.36 (*C*H_2_OH), 81.07 (C-3), 110.04 (C-29), 150.17 (C-20), 171.05, 171.18, 171.93, 173.59 (4 × CO) ppm.

##### 3-OAc-28-*O*-[Suc-Thr(OMe)]-BN (**3f**)

Viscous oil (52% yield, Method B); R_f_ = 0.85 (AcOEt); CC (AcOEt); HRMS (ESI^+^): *m*/*z*: calcd for C_41_H_65_NO_8_Na [M+Na]^+^ 722.4608, found 722.4603; [α]^25^_D_ = 3.6, (c = 1.1, CHCl_3_); ^1^H NMR (600 MHz, CDCl_3_): δ_H_ 0.83, 0.84, 0.96, 1.02 (all s, 15H, CH_3_-23–CH_3_-27), 1.22 (d, 3H, *J* = 7.2 Hz, C*H*_3_CH(OH)) 1.68 (s, 3H, CH_3_-30), 0.70–2.00 (m, 27H, CH, CH_2_, BN scaffold and C*H*_3_CH), 2.04 (s, 3H, CH_3_CO), 2.43 (td, 1H, *J*_1_ = 5.4 Hz, *J*_2_ = 10.8 Hz, H-19), 2.59–2.80 (m, 4H, O(CO)CH_2_CH_2_), 3.77 (s, 3H, OCH_3_), 3.89 (d, 1H, *J* = 11.4 Hz, H-28b), 4.27 (d, 1H, *J* = 11.4 Hz, H-28a), 4.33 (m, 1H, OH), 4.46 (dd, 1H, *J*_1_ = 5.4 Hz, *J*_2_ = 10.2 Hz, H-3), 4.59 (s, 1H, H-29b), 4.59-4.61 (m, 1H, NHC*H*), 4.68 (s, 1H, H-29a), 6.48 (s, br, 1H, NH) ppm; ^13^C NMR (150 MHz, CDCl_3_): δ_C_ 14.85 (C-27), 16.14 (C-24), 16.28 (C-26), 16.61 (C-25), 18.29 (C-6), 19.23 (C-30), 20.12 (CH(OH)*C*H_3_), 20.91 (C-11), 21.44 (*C*H_3_CO), 23.81 (C-2), 25.27 (C-12), 27.16 (C-15), 28.06 (C-23), 29.67 (C-16), 29.67 and 29.85 ((CO)*C*H_2_*C*H_2_), 31.05 (C-21), 34.24 (C-7), 34.63 (C-22), 37.18 (C-10), 37.70 (C-13), 37.92 (C-4), 38.50 (C-1), 41.01 (C-8), 42.82 (C-14), 46.53 (C-17), 47.82 (C-19), 48.90 (C-18), 50.39 (C-9), 52.71 (OCH_3_), 55.49 (C-5), 57.39 (NHC), 63.25 (C-28), 68.30 (CH(OH)*C*H_3_), 81.07 (C-3), 110.02 (C-29), 150.22 (C-20), 171.19, 171.61, 172.10, 173.40 (4 × CO) ppm.

##### 3-OAc-28-*O*-[Suc-Met(OMe)]-BN (**3g**)

Viscous oil (66% yield, Method B); R_f_ = 0.89 (AcOEt); CC (AcOEt); HRMS (ESI^+^): *m*/*z*: calcd for C_42_H_68_NO_7_S [M+H]^+^ 730.4716, found 730.4725; [α]^25^_D_ = 2.2, (c = 1.2, CHCl_3_); ^1^H NMR (600 MHz, CDCl_3_): δ_H_ 0.83, 0.84, 0.96, 1.02 (all s, 15H, CH_3_-23–CH_3_-27), 1.68 (s, 3H, CH_3_-30), 0.78–2.02 (m, 24H, CH, CH_2_, BN scaffold), 2.04 (s, 3H, CH_3_CO), 2.10 (s, 3H, SCH_3_), 2.14-2.20 (m, 4H, SCH_2_CH_2_), 2.43 (td, 1H, *J*_1_ = 5.4 Hz, *J*_2_ = 11.4 Hz, H-19), 2.51–2.76 (m, 4H, O(CO)CH_2_CH_2_), 3.76 (s, 3H, OCH_3_), 3.88 (d, 1H, *J* = 10.8 Hz, H-28b), 4.28 (d, 1H, *J* = 10.8 Hz, H-28a), 4.47 (dd, 1H, *J*_1_ = 5.4 Hz, J_2_ = 9.9 Hz, H-3), 4.59 (s, br, 1H, H-29b), 4.68 (s, br, 1H, H-29a), 4.70-4.73 (m, 1H, NHC*H*), 6.32 (d, 1H, *J* = 7.8 Hz, NH) ppm; ^13^C NMR (150 MHz, CDCl_3_): δ_C_ 14.86 (C-27), 15.63 (SCH_3_), 16.16 (C-24), 16.29 (C-26), 16.62 (C-25), 18.30 (C-6), 19.26 (C-30), 20.92 (C-11), 21.46 (*C*H_3_CO), 23.83 (C-2), 25.29 (C-12), 27.17 (C-15), 28.08 (C-23), 29.59 (C-16), 29.71 and 29.86 ((CO)*C*H_2_*C*H_2_), 31.09 (C-21), 30.05 i 31.89 (*C*H_2_*C*H_2_S), 34.24 (C-7), 34.66 (C-22), 37.20 (C-10), 37.72 (C-13), 37.93 (C-4), 38.52 (C-1), 41.03 (C-8), 42.83 (C-14), 46.55 (C-17), 47.85 (C-19), 48.92 (C-18), 50.40 (C-9), 51.71 (NHC), 52.68 (OCH_3_), 55.51 (C-5), 63.23 (C-28), 81.06 (C-3), 110.02 (C-29), 150.25 (C-20), 171.16, 171.35, 172.55, 173.31 (4 × CO) ppm.

##### 3-OAc-28-*O*-[Suc-Tyr(OMe)]-BN (**3h**)

Viscous oil (46% yield, Method B); R_f_ = 0.96 (AcOEt); CC (AcOEt); HRMS (ESI^+^): *m*/*z*: calcd for C_46_H_67_NO_8_Na [M+Na]^+^ 784.4764, found 784.4768; [α]^25^_D_ = 12.7, (c = 1.1, CHCl_3_); ^1^H NMR (600 MHz, CDCl_3_): δ_H_ 0.83, 0.84, 0.96, 1.02 (all s, 15H, CH_3_-23–CH_3_-27), 1.68 (s, 3H, CH_3_-30), 0.77–1.99 (m, 24H, CH, CH_2_, BN scaffold), 2.04 (s, 3H, CH_3_CO), 2.43 (td, 1H, *J*_1_ = 5.4 Hz, *J*_2_ = 10.8 Hz, H-19), 2.49–2.70 (m, 4H, O(CO)CH_2_CH_2_), 3.04 (dd, 2H, *J*_1_ = 6.0 Hz, *J*_2_ = 14.4 Hz, NHCHC*H*_2_), 3.72 (s, 3H, OCH_3_), 3.88 (d, 1H, *J* = 10.8 Hz, H-28b), 4.28 (d, 1H, *J* = 11.4 Hz, H-28a), 4.47 (dd, 1H, *J*_1_ = 5.4 Hz, *J*_2_ = 10.8 Hz, H-3), 4.59 (s, br, 1H, H-29b), 4.69 (s, br, 1H, H-29a), 4.82–4.85 (m, 1H, NHC*H*), 5.54 (s, br, 1H, OH), 6.12 (d, 1H, *J* = 7.8 Hz, NH), 6.74 (d, 2H, *J* = 8.4 Hz, Ph), 6.96 (d, 2H, *J* = 8.4 Hz, Ph) ppm; ^13^C NMR (150 MHz, CDCl_3_): δ_C_ 14.87 (C-27), 16.18 (C-24), 16.30 (C-26), 16.64 (C-25), 18.31 (C-6), 19.26 (C-30), 20.94^a^ (C-11), 20.94 (*C*H_2_C_6_H_4_OH), 21.47 (*C*H_3_CO), 23.84 (C-2), 25.30 (C-12), 27.19 (C-15), 28.09 (C-23), 29.55 (C-16), 29.72 and 29.87 ((CO)*C*H_2_*C*H_2_), 31.03 (C-21), 34.26 (C-7), 34.68 (C-22), 37.21 (C-10), 37.73 (C-13), 37.94 (C-4), 38.52 (C-1), 41.04 (C-8), 42.84 (C-14), 46.56 (C-17), 47.86 (C-19), 48.93 (C-18), 50.42 (C-9), 52.49 (OCH_3_), 53.49 (NHC), 55.52 (C-5), 63.28 (C-28), 81.12 (C-3), 110.03 (C-29), 150.26 (C-20), 115.64, 127.70, 130.57, 155.15 (C_6_H_4_OH), 171.14, 171.24, 172.22, 173.36 (4 × CO) ppm.

##### 3-OAc-28-*O*-[Suc-Trp(OMe)]-BN (**3i**)

Viscous oil (88% yield, Method B); R_f_ = 0.93 (DCM/MeOH, 10:1); CC (DCM/MeOH, 10:1); HRMS (ESI^+^): *m*/*z*: calcd for C_48_H_68_N_2_O_7_Na [M+Na]^+^ 807.4924, found 807.4926; [α]^25^_D_ = 6.9, (c = 1.0, CHCl_3_); ^1^H NMR (600 MHz, CDCl_3_): δ_H_ 0.83, 0.84, 0.96, 1.02 (all s, 15H, CH_3_-23–CH_3_-27), 1.68 (s, 3H, CH_3_-30), 0.73–1.99 (m, 24H, CH, CH_2_, BN scaffold), 2.04 (s, 3H, CH_3_CO), 2.42 (td, 1H, *J*_1_ = 6.0 Hz, *J*_2_ = 12.0 Hz H-19), 2.46–2.73 (m, 4H, O(CO)CH_2_CH_2_), 3.30–3.31 (m, NHCHC*H*_2_), 3.68 (s, 3H, OCH_3_), 3.85 (d, 1H, *J* = 11.4 Hz, H-28b), 4.27 (d, 1H, *J* = 11.4 Hz, H-28a), 4.46 (dd, 1H, *J*_1_ = 6.6 Hz, *J*_2_ = 10.2 Hz, H-3), 4.58 (s, br, 1H, H-29b), 4.68 (s, br, 1H, H-29a), 4.90–4.95 (m, 1H, NHC*H*), 6.39 (d, 1H, *J* = 7.8 Hz, NH), 7.02-7.53 (m, 5H, C_8_H_5_NH), 8.74 (s, br, 1H, NH) ppm; ^13^C NMR (150 MHz, CDCl_3_): δ_C_ 14.78 (C-27), 16.06 (C-24), 16.21 (C-26), 16.54 (C-25), 18.23 (C-6), 19.16 (C-30), 20.86 (C-11), 21.37 (*C*H_3_CO), 23.75 (C-2), 25.22 (C-12), 27.10 (C-15), 27.68 (CH_2_), 28.00 (C-23), 29.45 (C-16), 29.63 and 29.77 ((CO)*C*H_2_*C*H_2_), 30.84 (C-21), 34.17 (C-7), 34.57 (C-22), 37.13 (C-10), 37.65 (C-13), 37.86 (C-4), 38.44 (C-1), 40.96 (C-8), 42.76 (C-14), 46.48 (C-17), 47.79 (C-19), 48.85 (C-18), 50.33 (C-9), 52.41 (OCH_3_), 53.23 (NHC), 55.44 (C-5), 63.15 (C-28), 81.19 (C-3), 109.93 (C-29), 109.69, 111.36, 118.51, 119.57, 122.12, 122.97, 127.68 and 136.14 (C_8_H_6_N), 150.20 (C-20), 171.30, 171.39, 172.50, 173.35 (4 × CO) ppm.

##### 3-OAc-28-*O*-[Suc-Pro(OMe)]-BN (**3j**)

Viscous oil (52% yield, Method B); R_f_ = 0.89 (DCM/MeOH, 50:1); CC (DCM/MeOH, 50:1); HRMS (ESI^+^): *m*/*z*: calcd for C_42_H_65_NO_7_ [M+H]^+^ 718.4659, found 718.4650; [α]^25^_D_ = −20.4, (c = 1.0, CHCl_3_); ^1^H NMR (600 MHz, CDCl_3_): δ_H_ 0.83, 0.84, 0.96, 1.02, (all s, 15H, CH_3_-23–CH_3_-27), 1.68 (s, 3H, CH_3_-30), 0.77–2.20 (m, 30H, CH, CH_2_, BN scaffold, Pro:CH_2_CH_2_CH_2_), 2.04 (s, 3H, CH_3_CO), 2.42 (td, 1H, *J*_1_ = 5.4 Hz, J_2_ = 10.8 Hz, H-19), 2.58–2.80 (m, 4H, O(CO)CH_2_CH_2_), 3.71 (s, 3H, OCH_3_), 3.54–3.68 (m, 2H, CH_2_), 3.83 (d, 1H, *J* = 11.4 Hz, H-28b), 4.31 (d, 1H, *J* = 11.4 Hz, H-28a), 4.45-4.50 (m, 2H, H-3 and NCH), 4.59 (s, br, 1H, H-29b), 4.68 (s, br, 1H, H-29a) ppm; ^13^C NMR (150 MHz, CDCl_3_): δ_C_ 14.84 (C-27), 16.15 (C-24), 16.27 (C-26), 16.61 (C-25), 18.29 (C-6), 19.24 (C-30), 20.92 (C-11), 21.44 (*C*H_3_CO), 23.82 (C-2), 24.85 (CH_2_), 25.28 (C-12), 27.19 (C-15), 28.07 (C-23), 29.07 (CH_2_), 29.34 (C-16), 29.73 and 29.87 ((CO)*C*H_2_*C*H_2_), 31.56 (C-21), 34.23 (C-7), 34.67 (C-22), 37.19 (C-10), 37.69 (C-13), 37.92 (C-4), 38.51 (C-1), 41.02 (C-8), 42.82 (C-14), 46.56 (C-17), 46.99 (NHCH_2_), 47.86 (C-19), 48.92 (C-18), 50.40 (C-9), 52.32 (OCH_3_), 55.50 (C-5), 58.79 (CH), 62.99 (C-28), 81.06 (C-3), 109.95 (C-29), 150.30 (C-20), 170.23, 171.13, 172.95, 173.51 (4 × CO) ppm.

#### 3.1.3. Procedure for Preparation of 28-O’-(3′-Carboxypropanoyl)betulin (**4**)

BN (4.50 mmol, 2.0 g), SA (6.75 mmol, 0.68 g, 1.5 eq.) and DMAP (6.75 mmol, 0.82 g, 1.5 eq.) were dissolved in dry pyridine (36 mL). The reaction mixture was stirred under reflux for 8 h. After cooling, 10% hydrochloric acid solution (80 mL) and water (100 mL) were added. The product was extracted with CHCl_3_ (4 × 100 mL). The combined organic layers were concentrated to a 100 mL volume, and washed with water (60 mL), 5% hydrochloric acid solution (2 × 120 mL), brine (100 mL), water (60 mL) and dried over MgSO_4_. After that followed by filtration and concentrated *in vacuo*. The residue was purified by column chromatography (DCM/MeOH, gradient 100:1 to 20:1).

Viscous oil (67% yield); R_f_ = 0.25 (DCM/MeOH, 20:1); HRMS (ESI^+^) *m*/*z*: calcd for C_34_H_54_O_5_Na [M+Na]^+^ 565.3869, found 565.3860; ^1^H NMR (600 MHz, CDCl_3_): δ_H_ 0.75, 0.82, 0.96, 0.97, 1.03 (all s, 3H each, CH_3_-23–CH_3_-27), 1.68 (s, 3H, CH_3_-30), 0.67–2.08 (m, 24H, CH, CH_2_, BN scaffold), 2.43 (td, 1H, *J*_1_ = 5.8 Hz, *J*_2_ = 11.1 Hz, H-19), 2.56–2.66 (m, 4H, O(CO)CH_2_CH_2_), 3.17 (dd, 1H, *J*_1_ = 5.7 Hz, *J*_2_ = 10.8 Hz, H-3), 3.23–3.58 (s, br, 1H, OH), 3.88 (d, 1H, *J* = 10.8 Hz, H-28b), 4.29 (d, 1H, *J* = 11.4 Hz, H-28a), 4.59 (s, br, 1H, H-29b), 4.69 (s, br, 1H, H-29a), ppm; ^13^C NMR (150 MHz, CDCl_3_): δ_C_ 14.77 (C-27), 15.37 (C-24), 15.99 (C-26), 16.08 (C-25), 18.31 (C-6), 19.13 (C-30), 20.80 (C-11), 25.24 (C-12), 27.09 (C-2), 27.06 (C-15), 27.93 (C-23), 29.59 (C-16), 29.38 and 29.74 (CO)*C*H_2_*C*H_2_), 30.94 (C-21), 34.21 (C-7), 34.51 (C-22), 37.16 (C-10), 37.64 (C-13), 38.76 (C-4), 38.84 (C-1), 40.90 (C-8), 42.72 (C-14), 46.46 (C-17), 47.74 (C-19), 48.85 (C-18), 50.40 (C-9), 55.35 (C-5), 63.14 (C-28), 78.90 (C-3), 109.84 (C-29), 150.17 (C-20), 172.32, 173.08, 177.76 (3 × CO) ppm.

#### 3.1.4. General Procedure for the Synthesis of Hybrids 3-OH-28-O-[Suc-AA]-BN (**5a**–**b**)

3-OH-28-*O*’-(3′-Carboxypropanoyl)betulin (**4**, 0.20 mmol, 108.4 mg), DCC (0.30 mmol, 61.9 mg, 1.5 eq.), HOBt (0.20 mmol, 30.6 mg, 1 eq.) and H_2_NCH(R)COOMe·HCl (0.30 mmol, 1.5 eq.) in dry DCM (9–14 mL) stirred for 1 h at room temperature under argon atmosphere. Then, Et_3_N (0.30 mmol, 30.4 mg, 0.04 mL, 1.5 eq.) and methanol (0.9–1.4 mL) was added dropwise. The reaction was carried out at room temperature for 2–5 days. After the reaction was completed (was monitored on ^1^H NMR), the mixture was concentrated *in vacuo* and the residue extracted with AcOEt. The organic layers were combined, the solvent was removed under reduced pressure. Then, crude products **5** were purified by column chromatography.

##### 3-OH-28-*O*-[Suc-Gly(OMe)]-BN (**5a**)

Viscous oil (75% yield) R_f_ = 0.32 (DCM/MeOH, 20:1); CC (DCM/MeOH, gradient: 50:1 to 20:1); HRMS (ESI^+^): *m*/*z*: calcd for C_37_H_59_NO_6_Na [M+Na]^+^ 636.4240, found 636.4234; [α]^25^_D_ = 4.5 (c = 1.0, CHCl_3_); ^1^H NMR (600 MHz, CDCl_3_): δ_H_ 0.76, 0.82, 0.96, 0.97, 1.02 (all s, 15H, CH_3_-23–CH_3_-27), 1.68 (s, 3H, CH_3_-30), 0.63–1.98 (m, 24H, CH, CH_2_, BN scaffold), 2.42 (td, 1H, *J*_1_ = 5.8 Hz, *J*_2_ = 11.1 Hz, H-19), 2.55–2.72 (m, 4H, O(CO)CH_2_CH_2_), 3.18 (dd, 1H, *J*_1_ = 4.8 Hz, *J*_2_ = 11.4 Hz, H-3), 3.76 (s, 3H, OCH_3_), 3.88 (d, 1H, *J* = 11.4 Hz, H-28b), 4.04 (d, 2H, *J* = 5.4 Hz, NHC*H*_2_), 4.28 (d, 1H, *J* = 10.8 Hz, H-28a), 4.58 (s, br, 1H, H-29b), 4.68 (s, br, 1H, H-29a), 6.20 (s, br, 1H, NH) ppm; ^13^C NMR (150 MHz, CDCl_3_): δ_C_ 14.90 (C-27), 15.50 (C-24), 16.18 (C-26), 16.23 (C-25), 18.43 (C-6), 19.28 (C-30), 20.93 (C-11), 25.27 (C-12), 27.54 (C-2), 27.20 (C-15), 28.13 (C-23), 29.65 (C-16), 30.94 (C-21), 29.73 and 29.88 (CO)*C*H_2_*C*H_2_), 34.33 (C-7), 34.66 (C-22), 37.30 (C-10), 37.75 (C-13), 39.00 (C-4), 38.86 (C-1), 41.45 (NHC), 41.02 (C-8), 42.84 (C-14), 46.55 (C-17), 47.84 (C-19), 48.95 (C-18), 52.52 (OCH_3_), 50.52 (C-9), 55.44 (C-5), 63.26 (C-28), 79.10 (C-3), 109.98 (C-29), 150.25 (C-20), 170.48, 171.67, 173.33 (3 × CO) ppm.

##### 3-OH-28-*O*-[Suc-Ser(OMe)]-BN (**5b**)

Viscous oil (78% yield) R_f_ = 0.42 (DCM/MeOH, 20:1); CC (DCM/MeOH, gradient: 50:1 to 20:1); HRMS (ESI^+^): *m*/*z*: calcd for C_38_H_61_NO_7_Na [M+Na]^+^ 666.4346, found 666.4341; [α]^25^_D_ = 14.3, (c = 1.0, CHCl_3_); ^1^H NMR (600 MHz, CDCl_3_): δ_H_ 0.76, 0.82, 0.97, 1.02 (all s, 15H, CH_3_-23–CH_3_-27), 1.68 (s, 3H, CH_3_-30), 0.67–1.98 (m, 24H, CH, CH_2_, BN scaffold), 2.42 (td, 1H, *J*_1_ = 6.0 Hz, *J*_2_ = 11.1 Hz, H-19), 2.52–2.72 (m, 4H, O(CO)CH_2_CH_2_), 2.77-2.82 (m, 1H, OH), 3.19 (dd, 1H, *J*_1_ = 4.8 Hz, *J*_2_ = 11.4 Hz, H-3), 3.80 (s, 3H, OCH_3_), 3.87 (d, 1H, *J* = 10.8 Hz, H-28b), 3.95 (d, 2H, *J* = 3.6 Hz, CHC*H*_2_), 4.28 (d, 1H, *J* = 10.8 Hz, H-28a), 4.58 (s, 1H, H-29b), 4.65 (m, 1H, NHC*H*), 4.68 (s, 1H, H-29a), 6.69 (d, 1H, NH) ppm; ^13^C NMR (150 MHz, CDCl_3_): δ_C_ 14.89 (C-27), 15.51 (C-24), 16.14 (C-26), 16.22 (C-25), 18.41 (C-6), 19.26 (C-30), 20.90 (C-11), 25.32 (C-12), 27.18 (C-15), 27.47 (C-2), 28.12 (C-23), 29.68 (C-16), 29.66^a^ and 29.85 (CO)*C*H_2_*C*H_2_), 31.03 (C-21), 34.32 (C-7), 34.64 (C-22), 37.27 (C-10), 37.73 (C-13), 38.84 (C-4), 38.98 (C-1), 41.00 (C-8), 42.82 (C-14), 46.53 (C-17), 47.80 (C-19), 48.92 (C-18), 50.49 (C-9), 52.86 (OCH_3_), 55.03 (NHC), 55.42 (C-5), 63.06 (C-28), 63.37 (CH_2_O), 79.19 (C-3), 110.00 (C-29), 150.17 (C-20), 171.08, 171.93, 173.62 (3 × CO) ppm.

#### 3.1.5. General Procedure for the Synthesis of 3,28-bis[*O*-Suc-AA]-BN (**7a**–**f**)

The solution of 3,28-*O*,*O*′-*bis*(3′-carboxypropanoyl)betulin (**6**, 0.20 mmol, 128.8 mg), DCC (0.50 mmol, 103.4 mg, 2.5 eq.), HOBt (0.40 mmol, 61.4 mg, 2 eq.), H_2_NCH(R)COOMe·HCl (0.50 mmol, 2.5 eq.) in dry DCM (7–10 mL) was stirred vigorous for 1 h at room temperature under argon atmosphere. Then, Et_3_N (0.50 mmol, 50.7 mg, 0.07 mL, 2.5 eq.) and methanol (0.5–1.5 mL) was added dropwise. The reaction was carried out at room temperature for 2–5 days. After the reaction was completed, the mixture was concentrated *in vacuo* and the residue was extracted with ethyl acetate. The organic layers were combined, the solvent was removed under reduced pressure. Then, crude products **7** were purified by column chromatography.

##### 3,28-bis[*O*-Suc-Gly(OMe)]-BN (**7a**)

Viscous oil (67% yield); R_f_ = 0.79 (AcOEt/MeOH, 50:1); CC (AcOEt/MeOH, 50:1); HRMS (ESI^+^): *m*/*z*: calcd for C_44_H_69_N_2_O_10_ [M+H]^+^ 785.4952, found 785.4951; [α]^25^_D_ = 9.3, (c = 1.0, CHCl_3_); ^1^H NMR (600 MHz, CDCl_3_): δ_H_ 0.83, 0.84, 0.96, 1.02 (all s, 15H, CH_3_-23–CH_3_-27), 1.68 (s, 3H, CH_3_-30), 0.70–2.10 (m, 24H, CH, CH_2_, BN scaffold), 2.42 (td, 1H, *J*_1_ = 6.0 Hz, *J*_2_ = 11.4 Hz, H-19), 2.54–2.73 (m, 8H, 2 × O(CO)CH_2_CH_2_), 3.77 (s, 3H, OCH_3_) and 3.76 (s, 3H, OCH_3_), 3.88 (d, 1H, *J* = 11.4 Hz, H-28b), 4.04 (d, 2H, *J* = 4.8 Hz, NHC*H*_2_), 4.05 (d, 2H, *J* = 5.4 Hz, NHC*H*_2_), 4.28 (d, 1H, *J* = 11.4 Hz, H-28a), 4.48 (m, 1H, H-3), 4.58 (s, 1H, H-29b), 4.68 (s, 1H, H-29a), 6.19 (s, 2H, 2 × NH) ppm; ^13^C NMR (150 MHz, CDCl_3_): δ_C_ 14.87 (C-27), 16.16 (C-24), 16.26 (C-26), 16.64 (C-25), 18.28 (C-6), 19.25 (C-30), 20.92 (C-11), 23.79 (C-2), 25.28 (C-12), 27.17 (C-15), 28.09 (C-23), 29.64 (C-16), 29.70, 29.85, 29.93, 30.92 (2 × ((CO)*C*H_2_*C*H_2_), 31.00 (C-21), 34.23 (C-7), 34.64 (C-22), 37.19 (C-10), 37.71 (C-13), 37.98 (C-4), 38.49 (C-1), 41.02 (C-8), 41.44 (2 × NHC), 42.82 (C-14), 46.56 (C-17), 47.86 (C-19), 48.92 (C-18), 50.40 (C-9), 52.51 and 52.52 (2 × OCH_3_), 55.52 (C-5), 63.25 (C-28), 81.58 (C-3), 110.01 (C-29), 150.25 (C-20), 170.48, 170.50, 171.66, 171.73, 172.73, 173.33 (6 × CO) ppm.

##### 3,28-bis[*O*-Suc-Ala(OMe)]-BN (**7b**)

Viscous oil (68% yield); R_f_ = 0.75 (AcOEt); CC (AcOEt/MeOH, 50:1); HRMS (ESI^+^): *m*/*z*: calcd for C_46_H_73_N_2_O_10_ [M+H]^+^ 813.5265, found 813.5262; [α]^25^_D_ = 8.7 (c = 1.0, CHCl_3_); ^1^H NMR (600 MHz, CDCl_3_): δ_H_ 0.83, 0.84, 0.96, 1.02 (all s, 15H, CH_3_-23–CH_3_-27), 1.68 (s, 3H, CH_3_-30), 0.70–2.00 (m, 30H, CH, CH_2_, BN scaffold and 2 × CHC*H*_3_), 2.42 (td, 1H, *J*_1_ = 6.0 Hz, *J*_2_ = 11.4 Hz, H-19), 2.51–2.74 (m, 8H, 2 × O(CO)CH_2_CH_2_), 3.74 (s, 3H, OCH_3_) and 3.75 (s, 3H, OCH_3_), 3.88 (d, 1H, *J* = 11.1 Hz, H-28b), 4.04 (d, 2H, *J* = 4.8 Hz, NHC*H*_2_), 4.05 (d, 2H, *J* = 5.4 Hz, NHC*H*_2_), 4.27 (d, 1H, *J* = 10.8 Hz, H-28a), 4.47–4.50 (m, 1H, H-3), 4.56–4.61 (m, 2H, H-29b and NHC*H*), 4.66 (s, br, 1H, H-29a), 6.30 (s, 2H, 2 × NH) ppm; ^13^C NMR (150 MHz, CDCl_3_): δ_C_ 14.82 (C-27), 16.10 (C-24), 16.21 (C-26), 16.62 (C-25), 18.23 (C-6), 18.53 (2 × CH_3_), 19.20 (C-30), 20.88 (C-11), 23.75 (C-2), 25.24 (C-12), 27.13 (C-15), 28.05 (C-23), 29.58 (C-16), 29.65, 29.81, 29.90 and 30.95 (2 × ((CO)*C*H_2_*C*H_2_), 31.04 (C-21), 34.19 (C-7), 34.61 (C-22), 37.14 (C-10), 37.66 (C-13), 37.94 (C-4), 38.44 (C-1), 40.97 (C-8), 42.78 (C-14), 46.50 (C-17), 47.80 (C-19), 48.14 (NHC), 48.17 (NHC), 48.87 (C-18), 50.35 (C-9), 52.52 (2 × OCH_3_), 55.47 (C-5), 63.13 (C-28), 81.44 (C-3), 109.97 (C-29), 150.18 (C-20), 171.01, 171.08, 172.64, 173.28, 173.59, 173.60 (6 × CO) ppm.

##### 3,28-bis[*O*-Suc-Val(OMe)]-BN (**7c**)

Viscous oil (82% yield); R_f_ = 0.78 (AcOEt); CC (AcOEt); HRMS (ESI^+^): *m*/*z*: calcd for C_50_H_81_N_2_O_10_ [M+H]^+^ 869.5891, found 869.5892; [α]^25^_D_ = 18.7, (c = 1.0, CHCl_3_); ^1^H NMR (600 MHz, CDCl_3_): δ_H_ 0.83, 0.84, 0.96, 1.02 (all s, 15H, CH_3_-23–CH_3_-27), 0.90 (d, 3H, *J* = 3.0 Hz, CH(C*H*_3_)_2_), 0.91 (d, 3H, *J* = 3.0 Hz, CH(C*H*_3_)_2_), 0.92 (d, 3H, J = 4.2 Hz, CH(C*H*_3_)_2_), 0.94 (d, 3H, *J* = 4.2 Hz, CH(C*H*_3_)_2_), 1.68 (s, 3H, CH_3_-30), 0.73–2.00 (m, 36H, CH, CH_2_, BN scaffold), 2.10–2.19 (m, 2H, 2 × C*H*(CH_3_)_2_), 2.39–2.46 (td, 1H, *J*_1_ = 8.4 Hz, *J*_2_ = 16.2 Hz H-19), 2.49–2.78 (m, 8H, 2 × O(CO)CH_2_CH_2_), 3.73 (s, 3H, OCH_3_), 3.74 (s, 3H, OCH_3_), 3.89 (d, 1H, *J* = 16.2 Hz, H-28b), 4.26 (d, 1H, *J* = 15.0 Hz, H-28a), 4.47–4.51 (m, 1H, H-3), 4.56 (dd, 1H, *J*_1_ = 4.8 Hz, *J*_2_ = 7.2 Hz, NHC*H*), 4.54 (dd, 1H, *J*_1_ = 4.5 Hz, *J*_2_ = 7.5 Hz, NHC*H*), 4.58 (s, br, 1H, H-29b), 4.68 (s, br, 1H, H-29a), 6.18 (s, br, 1H, NH), 6.20 (s, br, 1H, NH) ppm; ^13^C NMR (150 MHz, CDCl_3_): δ_C_ 14.86 (C-27), 16.14 (C-24), 16.26 (C-26), 16.66 (C-25), 18.28 (C-6), 17.93, 17.95, 19.03 and 19.05 (2 × CH(*C*H_3_)_2_), 19.24 (C-30), 20.93 (C-11), 23.80 (C-2), 25.29 (C-12), 27.18 (C-15), 28.11 (C-23), 29.69 (C-16), 29.75, 29.87, 30.08 and 31.19 (2 × ((CO)*C*H_2_*C*H_2_), 31.30 (C-21), 34.24 (C-7), 34.65 (C-22), 37.18 (C-10), 37.71 (C-13), 37.99 (C-4), 38.49 (C-1), 41.02 (C-8), 42.83 (C-14), 46.54 (C-17), 47.83 (C-19), 48.92 (C-18), 50.40 (C-9), 52.23 (OCH_3_), 52.25 (OCH_3_), 55.51 (C-5), 57.21 (NHC), 57.22 (NHC), 63.21 (C-28), 81.49 (C-3), 110.00 (C-29), 150.24 (C-20), 171.43, 171.51, 172.61, 172.74, 173.37 (5 × CO) ppm.

##### 3,28-bis[*O*-Suc-Ser(OMe)]-BN (**7d**)

Viscous oil (87% yield); R_f_ = 0.91 (DCM/MeOH, 1:10); CC (DCM/MeOH, 1:10); HRMS (ESI^+^): *m*/*z*: calcd for C_46_H_72_N_2_O_12_Na [M+Na]^+^ 867.4983, found 867.4985; [α]^25^_D_ = 1.3, (c = 1.3, CHCl_3_); ^1^H NMR (600 MHz, CDCl_3_): δ_H_ 0.83, 0.84, 0.96, 1.02 (all s, 15H, CH_3_-23–CH_3_-27), 1.68 (s, 3H, CH_3_-30), 0.76–1.98 (m, 24H, CH, CH_2_, BN scaffold), 2.42 (td, 1H, *J*_1_ = 6.0 Hz, *J*_2_ = 10.8 Hz, H-19), 2.52-2.81 (m, 8H, 2 × O(CO)CH_2_CH_2_), 3.17–3.21 (m, 2H, 2 × OH), 3.78 (s, 3H, OCH_3_), 3.79 (s, 3H, OCH_3_), 3.87 (d, 1H, *J* = 10.8 Hz, H-28b), 3.90–3.97 (m, 4H, 2 × C*H*_2_OH), 4.28 (d, 1H, *J* = 10.8 Hz, H-28a), 4.46–4.49 (m, 1H, H-3), 4.59 (s, br, 1H, H-29b), 4.64–4.66 (m, 2H, 2 × NHC*H*), 4.68 (s, br, 1H, H-29a), 6.74 (s, br, 1H, NH), 6.75 (s, br, 1H, NH) ppm; ^13^C NMR (150 MHz, CDCl_3_): δ_C_ 14.84 (C-27), 16.10 (C-24), 16.22 (C-26), 16.63 (C-25), 18.25 (C-6), 19.21 (C-30), 20.88 (C-11), 23.72 (C-2), 25.22 (C-12), 27.13 (C-15), 28.07 (C-23), 29.61 (C-16), 29.63, 29.80, 29.92 and 30.94 (2 × ((CO)*C*H_2_*C*H_2_), 31.03 (C-21), 34.19 (C-7), 34.60 (C-22), 37.14 (C-10), 37.67 (C-13), 37.95 (C-4), 38.44 (C-1), 40.97 (C-8), 42.79 (C-14), 46.50 (C-17), 47.78 (C-19), 48.86 (C-18), 50.34 (C-9), 52.81 (2 × OCH_3_), 54.93 (NHC), 54.96 (NHC), 55.44 (C-5), 63.01 (CH_2_OH), 63.04 (CH_2_OH), 63.31 (C-28), 81.68 (C-3), 110.03 (C-29), 150.11 (C-20), 171.09, 171.10, 171.94, 172.01, 172.98, 173.58 (6 × CO) ppm.

##### 3,28-bis[*O*-Suc-Tyr(OMe)]-BN (**7e**)

Viscous oil (60% yield); R_f_ = 0.80 (AcOEt); CC (AcOEt); HRMS (ESI^+^): *m*/*z*: calcd for C_58_H_80_N_2_O_12_Na [M+Na]^+^ 1019.5609, found 1019.5672; [α]^25^_D_ = 15.6, (c = 0.9, CHCl_3_); ^1^H NMR (600 MHz, CDCl_3_): δ_H_ 0.82, 0.83, 0.96, 1.01 (all s, 15H, CH_3_-23–CH_3_-27), 1.67 (s, 3H, CH_3_-30), 0.76–1.97 (m, 24H, CH, CH_2_, BN scaffold), 2.42 (td, 1H, *J*_1_ = 6.0 Hz, *J*_2_ = 11.4 Hz, H-19), 2.47–2.71 (m, 8H, 2 × O(CO)CH_2_CH_2_), 2.98–3.08 (m, 4H, 2 × NHCHC*H*_2_), 3.72 (s, 3H, OCH_3_) and 3.73 (s, 3H, OCH_3_), 3.87 (d, 1H, *J* = 11.4 Hz, H-28b), 4.28 (d, 1H, *J* = 10.8 Hz, H-28a), 4.47–4.49 (m, 1H, H-3), 4.58 (s, br, 1H, H-29b), 4.68 (s, br, 1H, H-29a), 4.81–4.85 (m, 2H, 2 ×NHC*H*), 5.53 (s, br, 1H, OH), 5.54 (s, br, 1H, OH), 6.13 (d, 1H, *J* = 7.8 Hz, NH), 6.14 (d, 1H, J = 7.8 Hz, NH), 6.72–6.74 (m, 4H, Ph), 6.95–6.97 (m, 4H, Ph) ppm; ^13^C NMR (150 MHz, CDCl_3_): δ_C_ 14.88 (C-27), 16.14 (C-24), 16.26 (C-26), 16.67 (C-25), 18.27 (C-6), 19.22 (C-30), 20.91 (C-11 and 2 × CH_2_), 23.77 (C-2), 25.27 (C-12), 27.17 (C-15), 28.10 (C-23), 29.56 (C-16), 29.69, 29.85, 29.91, 30.99 (2 × (CO)*C*H_2_*C*H_2_), 31.09 (C-21), 34.21 (C-7), 34.65 (C-22), 37.17 (C-10), 37.69 (C-13), 37.98 (C-4), 38.46 (C-1), 41.00 (C-8), 42.82 (C-14), 46.53 (C-17), 47.81 (C-19), 48.91 (C-18), 50.36 (C-9), 52.52 (2 × OCH_3_), 53.54 (NHC), 53.56 (NHC), 55.47 (C-5), 63.36 (C-28), 81.71 (C-3), 110.04 (C-29), 150.20 (C-20), 115.69, 127.25, 130.45, 130.46, 155.51 (2 × C_6_H_4_OH), 171.44, 171.55, 172.31, 172.85, 173.47 (5 × CO) ppm.

##### 3,28-bis[*O*-Suc-Trp(OMe)]-BN (**7f**)

Viscous oil (47% yield); R_f_ = 0.95 (DCM/MeOH, 1:1); CC (DCM/MeOH, gradient: 10:1 to 1:1); HRMS (ESI^+^): *m*/*z*: calcd for C_62_H_82_N_4_O_10_Na [M+Na]^+^ 1065.5929, found 1065.6036; [α]^25^_D_ = 9.5, (c = 1.0, CHCl_3_); ^1^H NMR (600 MHz, CDCl_3_): δ_H_ 0.81, 0.83, 0.96, 1.01 (all s, 15H, CH_3_-23–CH_3_-27), 1.67 (s, 3H, CH_3_-30), 0.75–1.98 (m, 24H, CH, CH_2_, BN scaffold), 2.41 (td, 1H, *J*_1_ = 6.0 Hz, *J*_2_ = 10.8 Hz, H-19), 2.45-2.64 (m, 8H, 2 × O(CO)CH_2_CH_2_), 3.26–3.29 (m, 4H, 2 × NHCHC*H*_2_), 3.66 (s, 3H, OCH_3_), 3.67 (s, 3H, OCH_3_), 3.85 (d, 1H, *J* = 11.4 Hz, H-28b), 4.27 (d, 1H, *J* = 11.4 Hz, H-28a), 4,43–4.46 (m, 1H, H-3), 4.58 (s, br, 1H, H-29b), 4.68 (s, br, 1H, H-29a), 4.84–4.88 (m, 2H, 2 × NHC*H*), 6.80 (s, br, 1H, NH), 6.81 (s, br, 1H, NH), 7.01–7.53 (m, 10H, 2 × C_8_H_5_), 9.47 (s, 2H, 2 × NH) ppm; ^13^C NMR (150 MHz, CDCl_3_): δ_C_ 14.60 (C-27), 15.84 (C-24), 15.97 (C-26), 16.33 (C-25), 18.03 (C-6), 18.95 (C-30), 20.69 (C-11), 23.48 (C-2), 25.06 (C-12), 26.93 (C-15), 27.49 (C-23), 27.77 (*C*H_2_C_8_H_6_N), 27.77 (*C*H_2_C_8_H_6_N), 29.31 (C-16), 29.46, 29.59, 29.64 and 30.52 (2 × (CO)*C*H_2_*C*H_2_), 30.59 (C-21), 33.65 (C-7), 34.37 (C-22), 36.95 (C-10), 37.51 (C-13), 37.72 (C-4), 38.25 (C-1), 40.78 (C-8), 42.60 (C-14), 46.31 (C-17), 47.62 (C-19), 48.89 (C-18), 50.17 (C-9), 52.17 (2 × OCH_3_), 53.14 (NHC), 53.23 (NHC), 55.29 (C-5), 63.04 (C-28), 81.53 (C-3), 109.73 (C-29), 109.14, 109.20, 111.32, 111.37, 118.15, 119.15, 121.71, 123.07, 123.23, 127.46, 136.18 and 136.33 (2 × CH_2_*C_8_*H_6_N), 150.00 (C-20), 171.55, 171.63, 171.71, 172.55, 172.75, 173.32 (6 × CO) ppm.

### 3.2. Biological Activity Evaluation

#### 3.2.1. Cell Lines

Breast (MCF-7) and colon (HCT-116) cancer cell lines were obtained from ATCC (Manassas, VA, USA). Normal fibroblasts (NHDF) were obtained from Lonza (Basel, Switzerland). The cells were cultured in DMEM/F12 supplemented with 10% FBS and 1× Antibiotic/Antimycotic Solution (Capricorn Scientific, Dreihausen, Germany). Cells were incubated under standard conditions (37 °C, 5% CO_2_).

#### 3.2.2. Cell Viability Assay

Cells were seeded into 96-well plates. When the cell confluence reached about 80%, the cells were treated with different concentrations of BNor BNAA molecular hybrids and 0.1% Tween 80. After 24 h of incubation, the cell viability was determined using the Cell Counting Kit-8 (Bimake, Houston, TX, USA) and a BioTek Epoch Microplate Reader (Winooski, VT, USA).

Data was analyzed using the Shapiro–Wilk normality test, followed by one-way ANOVA with a significant level of 0.05. Statistical analysis was performed using Statistica software v.13.3 (TIBCO Software, Palo Alto, CA, USA).

#### 3.2.3. Gene Expression Level

HCT 116 cells were treated with 100 µM BN or one of its derivatives (**3e**, **3f**, **7d**) for 24 h. Total RNA was then isolated from the cells using a Universal RNA/miRNA Purification Kit (EURx, Gdańsk, Poland). Changes in the expression of p21 and Bax genes were assessed by RT-qPCR using RT PCR Mix EvaGreen (A&A Biotechnology, Gdańsk, Poland) and the CFX96 Touch Real-Time PCR Detection System (Bio-Rad, Laboratories, Hercules, CA, USA). Primers for p21 were (forward) 5′-CTCTACATCTTCTGCCTTAGTCTC-3′ and (reverse) 5′-GGTCACCCTGCCCAACCTTA-3′. Primers for Bax were (forward) 5′-GTCTTTTTCCGAGTGGCAGC-3′ and (reverse) 5′-TGGTTCTGATCAGTTCCGGC-3′. Data were analyzed using the ΔΔCt method [51].

## 4. Conclusions

In this study, we report a series of new molecular hybrids, composed of two building blocks: a betulin backbone and selected amino acid methyl esters. We conjugated three BN derivatives (3-*O*-acetyl-28-*O*’-(3′-carboxypropanoyl)betulin **2**, 28-*O*’-(3′-carboxypropanoyl)betulin **4**, and *3*,28-*O*,*O*’-*bis*(3′-carboxypropanoyl)betulin **6**) with different amino acids *via* a flexible linker -CO-CH_2_-CH_2_-COOH. The developed synthesis method proceeds under mild conditions (room temperature) and ensures good product yields (Table 2). As a result of the described reactions, 18 new compounds were obtained, and their structures were confirmed by spectroscopic methods (^1^H, ^13^C NMR) and high-resolution mass spectrometry (HR-MS, Supplementary Materials).

Studies conducted on cell lines have shown that the biological activity of the synthesized BN derivatives depends on the type of amino acid attached (Figure 3 and Figure 4). The effects of the obtained derivatives may include both a protective effect (e.g., the tryptophan derivatives of BN, **3i** and **7f**), most likely associated with a reduction in oxidative stress, and a cytotoxic effect causing cell cycle arrest (e.g., the threonine derivative of BN **3f**) or the induction of the mitochondrial apoptosis pathway (e.g., the serine derivatives of BN **3e** and **7d**). The 3-OAc-28-*O*-[Suc-Gly(OMe)]-BN **3a** showed the most favorable selectivity profile, whereas BNAA (**3e**, **3f**, and **7d**) showed the strongest cytotoxic/cellular response. Overall, C-28 monosubstitution was sufficient to obtain active derivatives, while C-3/C-28 disubstitution did not uniformly improve biological activity. A comparison of the biological activity of three serine-based BN derivatives differing in the group attached to the carbon atom at position 3 (**3e**, **5b**, **7d**) suggests that the substituent at the C-3 position also modulates the activity of BN derivatives. These results encourage us to conduct further research and pursue the rational optimization of the BN scaffold.

## Figures and Tables

**Table 1:** Representative molecular hybrids of triterpenoids (BN, BA) with amino acids (AA).

Molecular Hybrid	Tested AA	Methodology	Best Research Effects ^a^			Ref.
			IC_50_ (µM), BNAA/BN	AA	Cell Line	
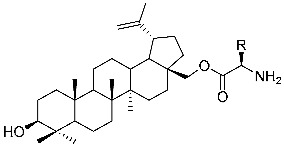	BN-Ala, Dap Dab, Orn Lys	MTT test	10.1/80.2 7.3/80.2	Orn Lys	A431 A431	[24]
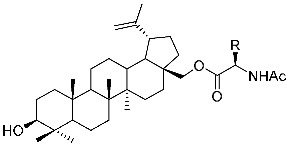	Gly, Ala Val, Leu Ile, Pro	MTT test	6.58/>100 4.24/>100 10.56/>100	Ala	HT 29 MPC2 MDAH2774	[25]
			14.09/>100 11.43/>100 10.87/>100	Val	HT 29 MPC2 MDAH2774	
			18.12/>100 19.71/>100 1.38/>100	Leu	HT 29 MPC2 MDAH2774	
			15.03/>100 10.17/>100 7.26/>100	Ile	HT 29 MPC2 MDAH2774	
			8.88/>100 5.32/>100 2.13/>100	Pro	HT 29 MPC2 MDAH2774	
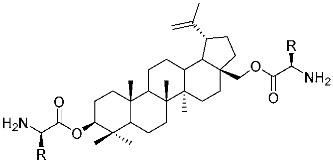	Gly, Ala, Phe, Lys Met	SRB test	4.6/50.0 3.8/50 4.1/26	Ala ^b^ Lys ^b^	EPP85-181P EPP85-181P EPG85-257P	[26]
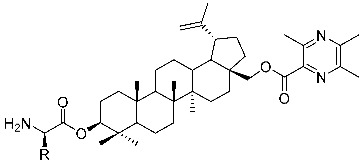	Gly, Ala Pro, Phe Ile, Leu Val, Pyr	MTT test	4.91/12.20 4.07/>20 4.24/>20M 7.83/>20 8.62/>20	Gly Ala Pyr	HeLa HepG2 BGC-823 BGC-823 BGC-823	[27]
			**IC_50_** (µM), **BAAA/BA**			
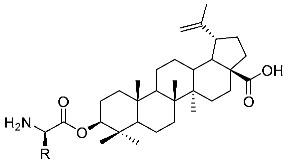	Ala, Phe Abu, Lys	SRB test	9.7/42	Lys ^b^	EPG85-257P	[26]
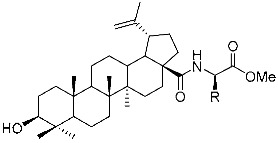	Gly, Ala, Phe, Val Leu, Met Trp, Asp Ile, Glu Tyr, Pro	SRB test	3.5/4.2 2.1/4.2	Ala ^b^ Val ^b^	MEL-2 MEL-2	[28]

^a^ Incubation time: 24 h; ^b^ Improvement in solubility.

**Table 2:** Yields of obtained BNAA hybrids (**3a**–**j**, **5a**–**b** and **7a**–**f**).

BN Analog	Attached Amino Acid	Betulin-Amino Acid Molecular Hybrids				
		No.	R^1^	R	Yield (%)	Acronym
**Modifications C-28**						
**2**	Gly(OMe)	**3a**	OAc	H	75	3-OAc-28-*O*-[Suc-Gly(OMe)]-BN
**2**	Ala(OMe)	**3b**	OAc	Me	68	3-OAc-28-*O*-[Suc-Ala(OMe)]-BN
**2**	Val(OMe)	**3c**	OAc	CHMe_2_	92	3-OAc-28-*O*-[Suc-Val(OMe)]-BN
**2**	Leu(OMe)	**3d**	OAc	CH_2_CHMe_2_	46	3-OAc-28-*O*-[Suc-Leu(OMe)]-BN
**2**	Ser(OMe)	**3e**	OAc	CH_2_OH	70	3-OAc-28-*O*-[Suc-Ser(OMe)]-BN
**2**	Thr(OMe)	**3f**	OAc	CH(OH)Me	52	3-OAc-28-*O*-[Suc-Thr(OMe)]-BN
**2**	Met(OMe)	**3g**	OAc	(CH_2_)_2_SMe	66	3-OAc-28-*O*-[Suc-Met(OMe)]-BN
**2**	Tyr(OMe)	**3h**	OAc	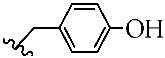	46	3-OAc-28-*O*-[Suc-Tyr(OMe)]-BN
**2**	Trp(OMe)	**3i**	OAc	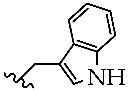	88	3-OAc-28-*O*-[Suc-Trp(OMe)]-BN
**2**	Pro(OMe)	**3j**	OAc	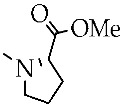	52	3-OAc-28-*O*-[Suc-Pro(OMe)]-BN
**4**	Gly(OMe)	**5a**	OH	H	75	3-OH-28-*O*-[Suc-Gly(OMe)]-BN
**4**	Ser(OMe)	**5b**	OH	CH_2_OH	78	3-OH-28-*O*-[Suc-Ser(OMe)]-BN
**Modifications C-3 and C-28**						
**6**	Gly(OMe)	**7a**	─	H	67	3,28-*bis*[*O*-Suc-Gly(OMe)]-BN
**6**	Ala(OMe)	**7b**	─	Me	68	3,28-*bis*[*O*-Suc-Ala(OMe)]-BN
**6**	Val(OMe)	**7c**	─	CHMe_2_	82	3,28-*bis*[*O*-Suc-Val(OMe)]-BN
**6**	Ser(OMe)	**7d**	─	CH_2_OH	87	3,28-*bis*[*O*-Suc-Ser(OMe)]-BN
**6**	Tyr(OMe)	**7e**	─	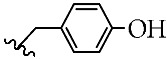	60	3,28-*bis*[*O*-Suc-Tyr(OMe)]-BN
**6**	Trp(OMe)	**7f**	─	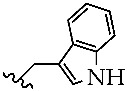	47	3,28-*bis*[*O*-Suc-Trp(OMe)]-BN

## Data Availability

The original contributions presented in this study are included in the article/Supplementary Materials. Further inquiries can be directed to the corresponding author(s).

## Supplementary Materials

The following supporting information can be downloaded at: https://www.mdpi.com/article/10.3390/ijms27104445/s1.
