# Supplementary material for: Betulin–Amino Acid Molecular Hybrids: Synthesis, Structure and Pharmacological Potential
Source: Int J Mol Sci. 2026 May 15;27(10):4445. doi: 10.3390/ijms27104445 (PMC13206932; doi:10.3390/ijms27104445)
Supplement: Supplementary file 1 [file ijms-27-04445-s001.zip › ijms-4304861-supplementary.pdf]

Supplementary Materials

Betulin–amino acid molecular hybrids: Synthesis, Structure and Pharmacological Potential

Mirosława Grymel[1,2,*,‡], Paweł Naprawca[1], Daria Dolniak-Budny[1], Mateusz D. Tomczyk[1], Mateusz Pielok[1], Beata Nowrot[3], Klaudia Skutnik[3], Karol Erfurt[4], Anna Lalik[2,3,*,‡]

[1] Department of Organic Chemistry, Bioorganic Chemistry and Biotechnology, Silesian University of Technology, B. Krzywoustego 4, 44-100 Gliwice, Poland; pawelnaprawca@interia.pl (P.N.); daria.dolniak@gmail.com (D.D.-B.); mp301255@student.polsl.pl (M.P.); mateusz.d.tomczyk@polsl.pl (M.D.T.)

[2] Biotechnology Center, Silesian University of Technology, B. Krzywoustego 8, 44-100 Gliwice, Poland

[3] Department of Systems Biology and Engineering, Silesian University of Technology, Akademicka 16, 44-100 Gliwice, Poland; beatnow835@student.polsl.pl (B.N.); ks308126@student.polsl.pl (K.S.)

[4] Department of Chemical Organic Technology and Petrochemistry, Silesian University of Technology, B. Krzywoustego 4, 44-100 Gliwice, Poland; karol.erfurt@polsl.pl (K.E.)

* Correspondence: miroslawa.grymel@polsl.pl (M.G.); anna.lalik@polsl.pl (A.L.); Tel.: +48-32-237-1873 (M.G.); Tel.: +48-32-237-2769 (A.L.)

‡ These authors contributed equally.

TABLE OF CONTENTS

Figures S01—S20: ^{1}H, ^{13}C NMR spectra of 3-OAc-28-*O*-[Suc-AA]-BN (**3a-j**) .... 2—21

Figures S21—S22: ^{1}H, ^{13}C NMR spectra of 28-*O*′-(3'-carboxypropanoyl)betulin (**4**) .... 22—23

Figures S23—S26: ^{1}H, ^{13}C NMR spectra of 3-OH-28-*O*-[Suc-AA]-BN (**5a-b**) .... 24—27

Figures S27—S38: ^{1}H, ^{13}C NMR spectra of 3,28-*bis*[*O*-Suc-AA]-BN (**7a-f**) .... 28—39

Table S1. One-way ANOVA summary table 40

Figure S39. Effect of the tested BNAA hybrids (**12e, 13e**) on the cell viability of HCT 116 and NHDF cells 41

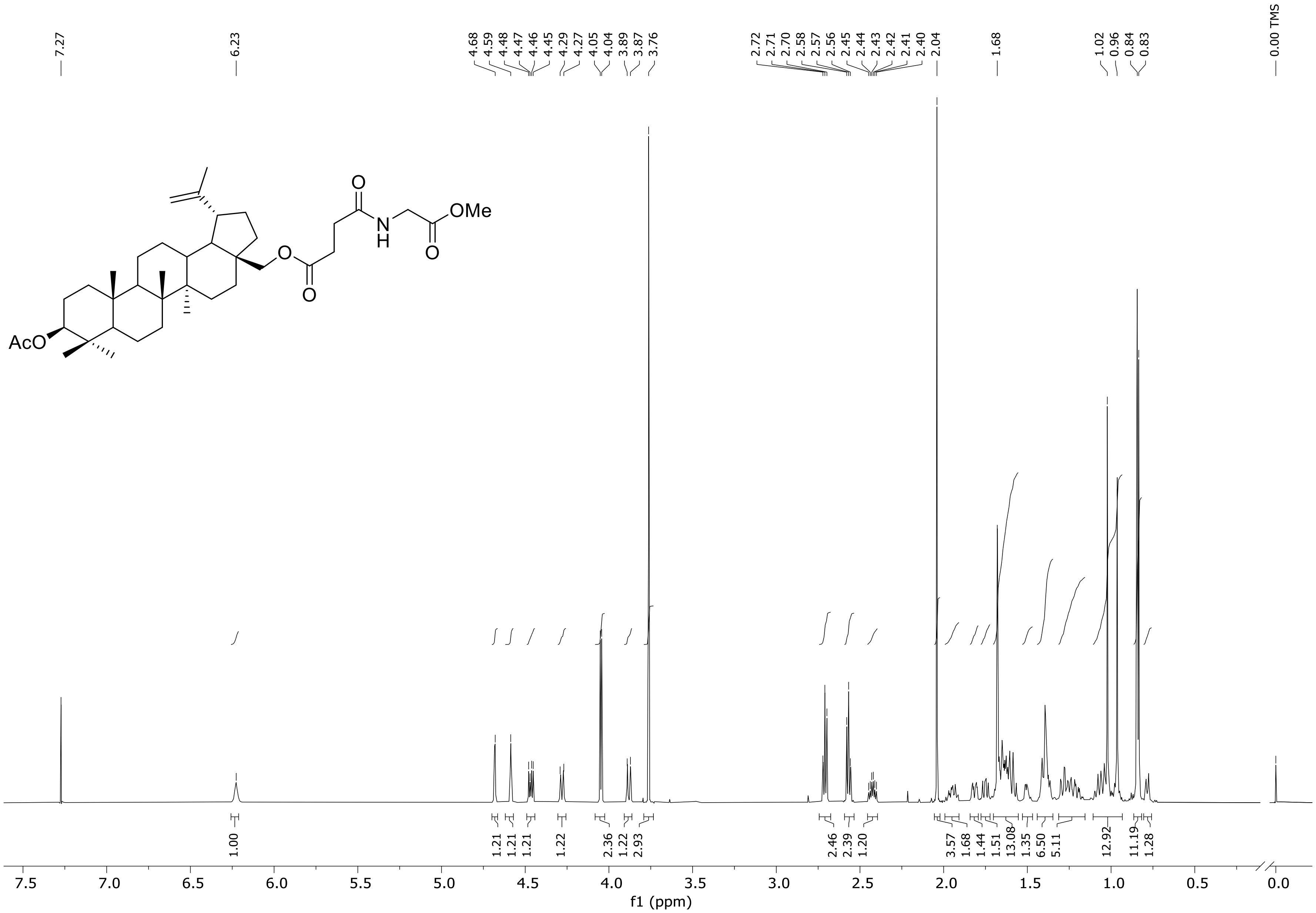

Figure S1. ^{1}H NMR spectrum of 3-OAc-28-O-[Suc-Gly(OMe)]-BN (**3a**); 600 MHz/$CDCl_3$/TMS; δ (ppm).

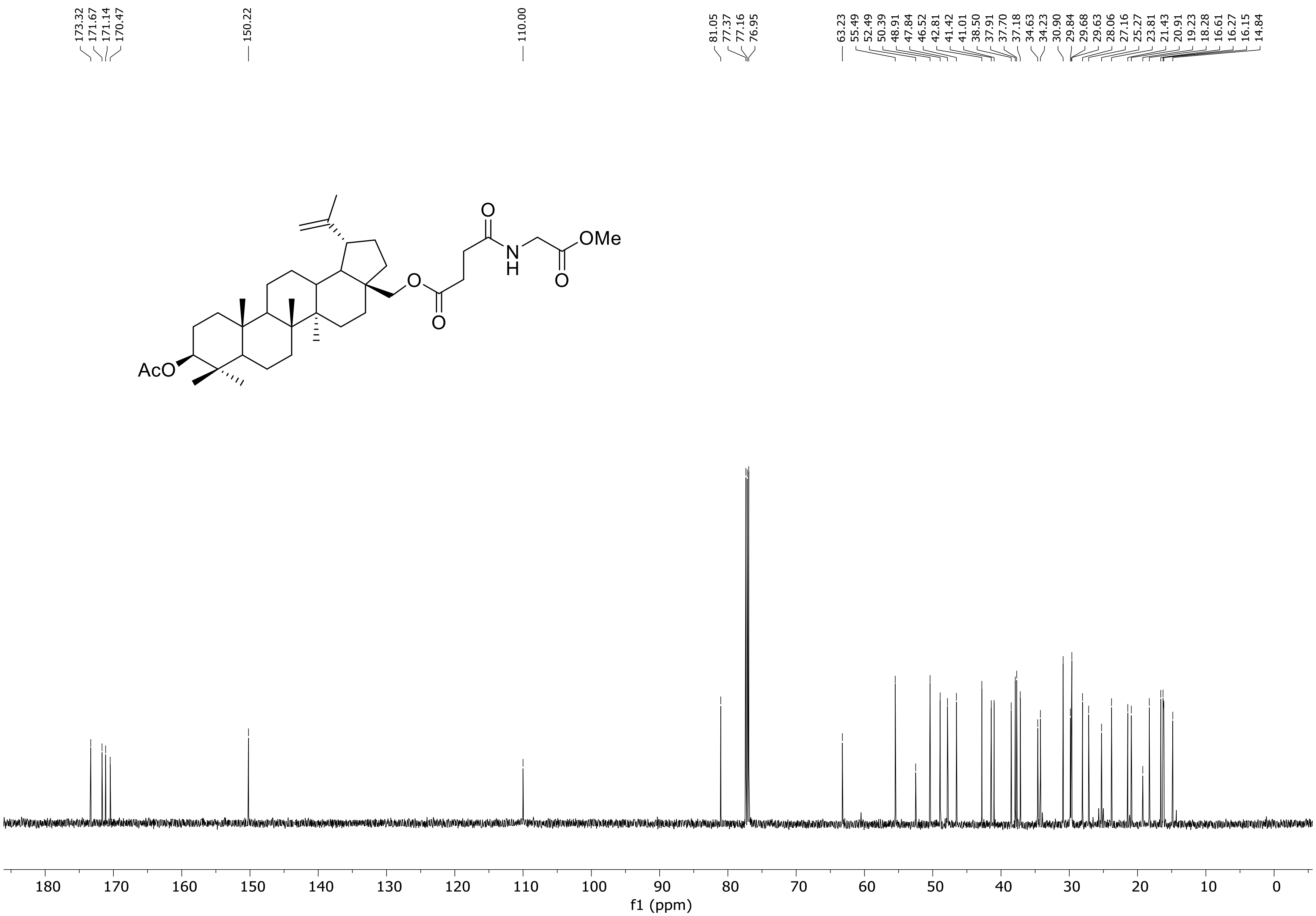

Figure S2. ^{13}C NMR spectrum of 3-OAc-28-O-[Suc-Gly(OMe)]-BN (**3a**); 150 MHz/$CDCl_3$/TMS; δ (ppm).

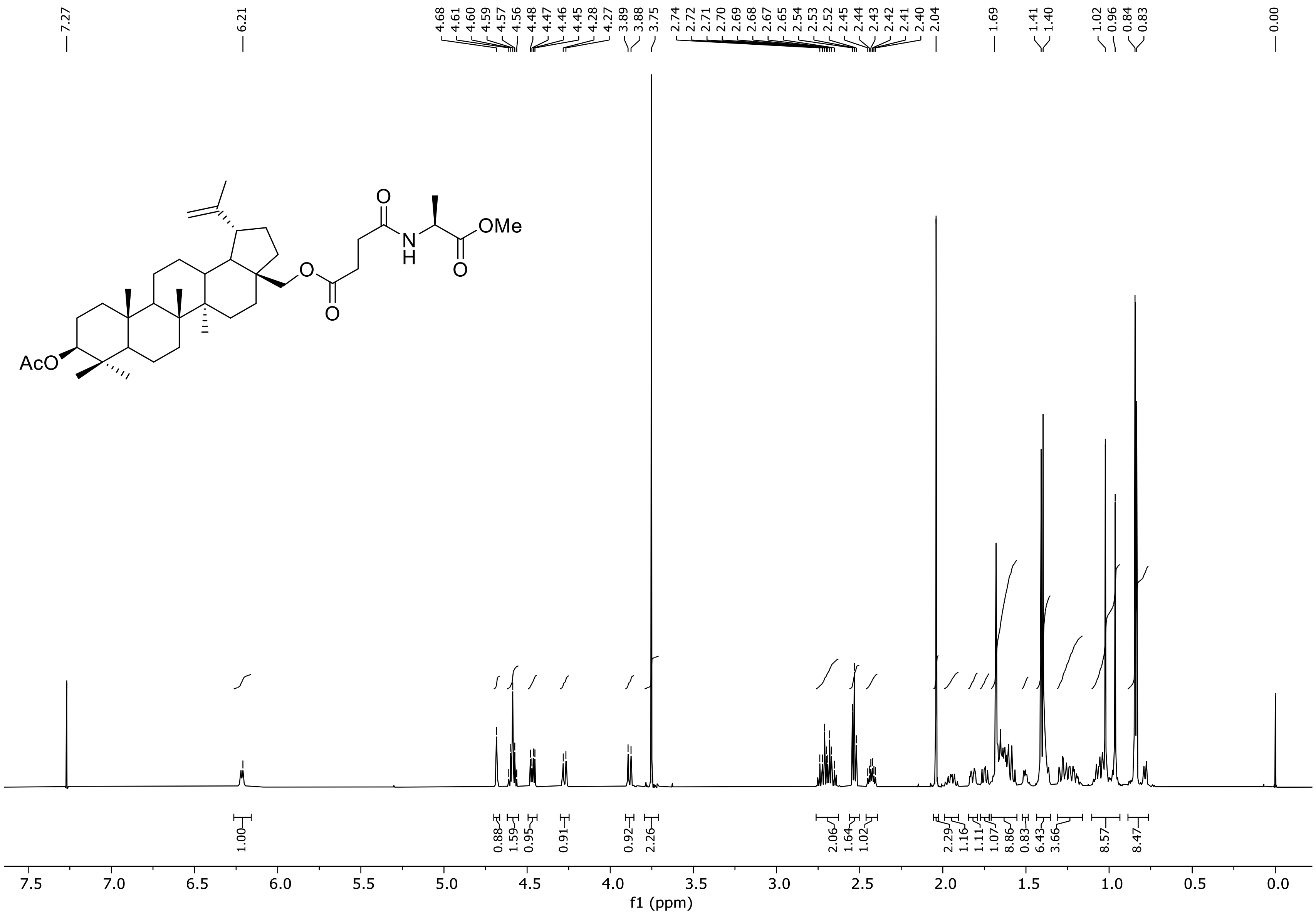

Figure S3. ^{1}H NMR spectrum of 3-OAc-28-O-[Suc-Ala(OMe)]-BN (**3b**); 600 MHz/$CDCl_3$/TMS; δ (ppm).

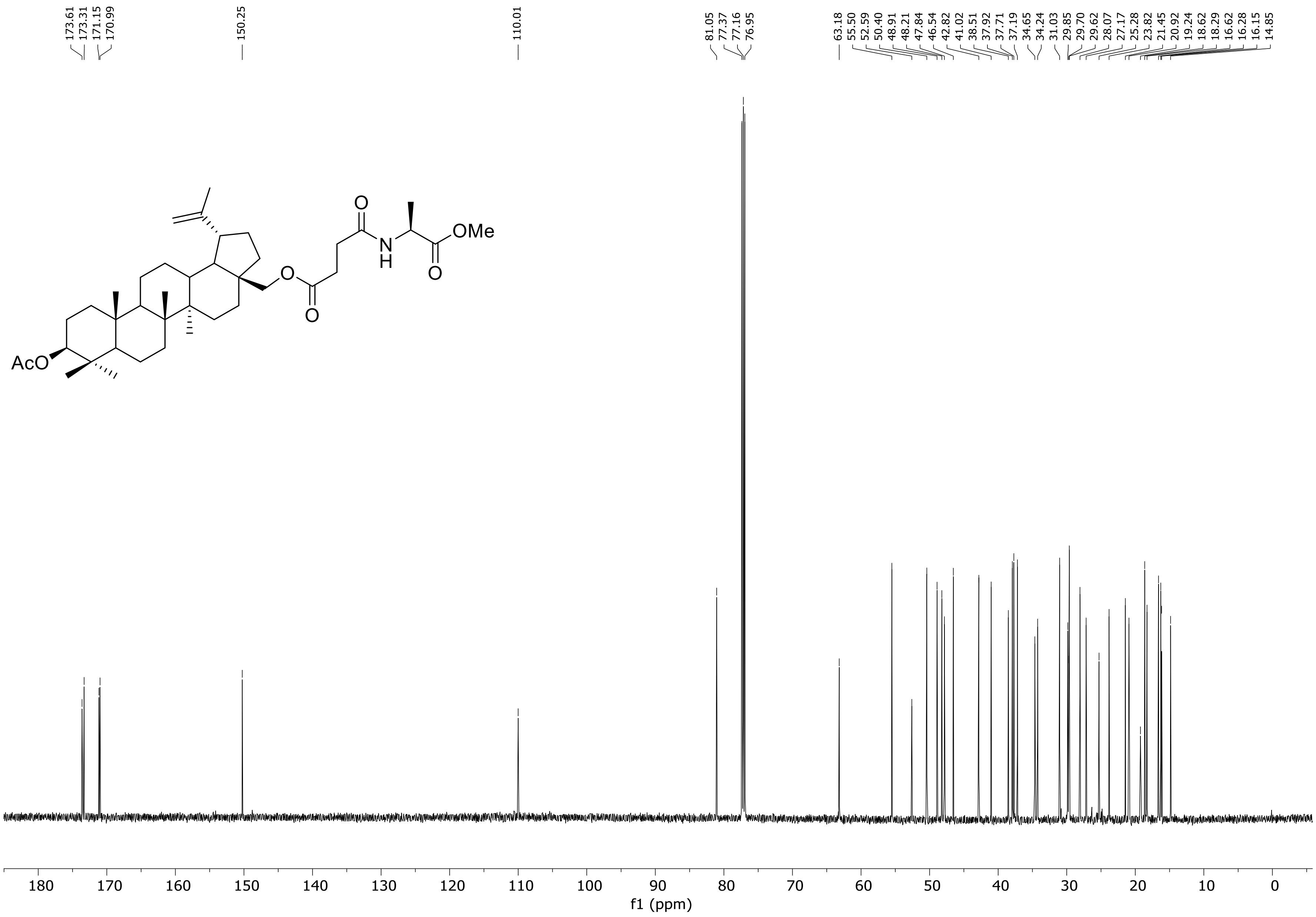

Figure S4. ^{13}C NMR spectrum of 3-OAc-28-O-[Suc-Ala(OMe)]-BN (**3b**); 150 MHz/$CDCl_3$/TMS; δ (ppm).

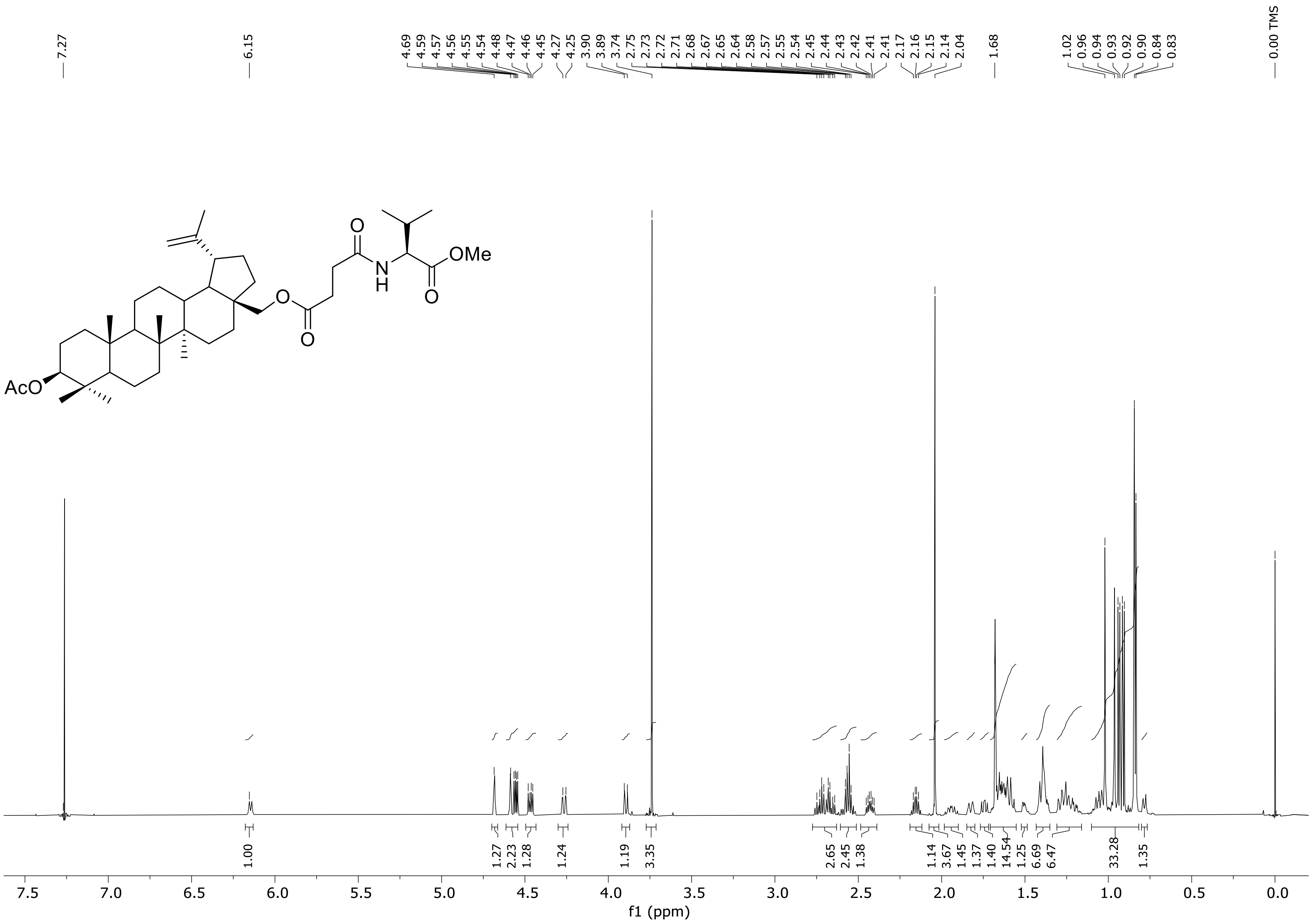

Figure S5. ^{1}H NMR spectrum of -OAc-28-O-[Suc-Val(OMe)]-BN (**3c**); 600 MHz/$CDCl_3$/TMS; δ (ppm).

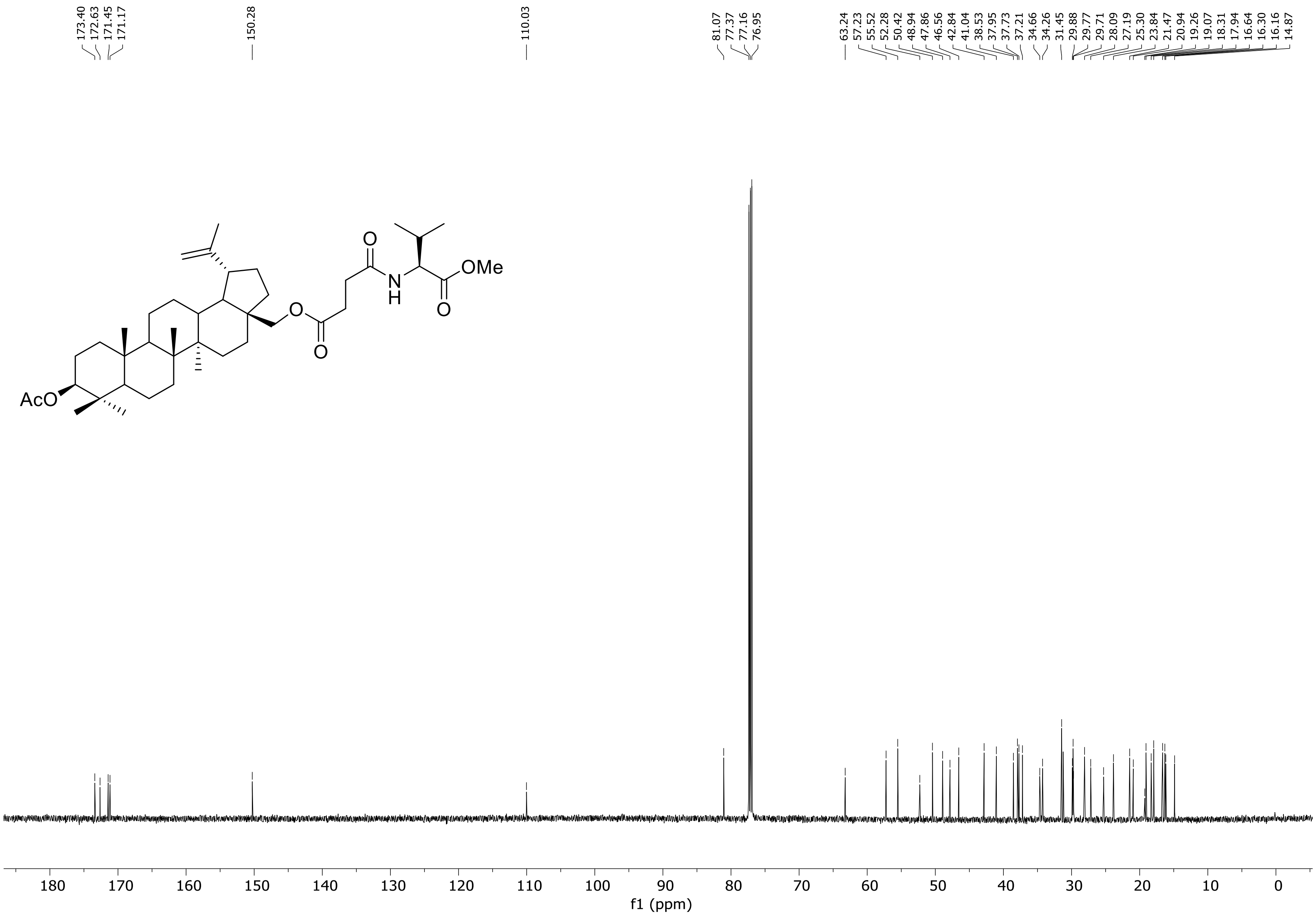

Figure S6. ^{13}C NMR spectrum of -OAc-28-O-[Suc-Val(OMe)]-BN (**3c**); 150 MHz/$CDCl_3$/TMS; δ (ppm).

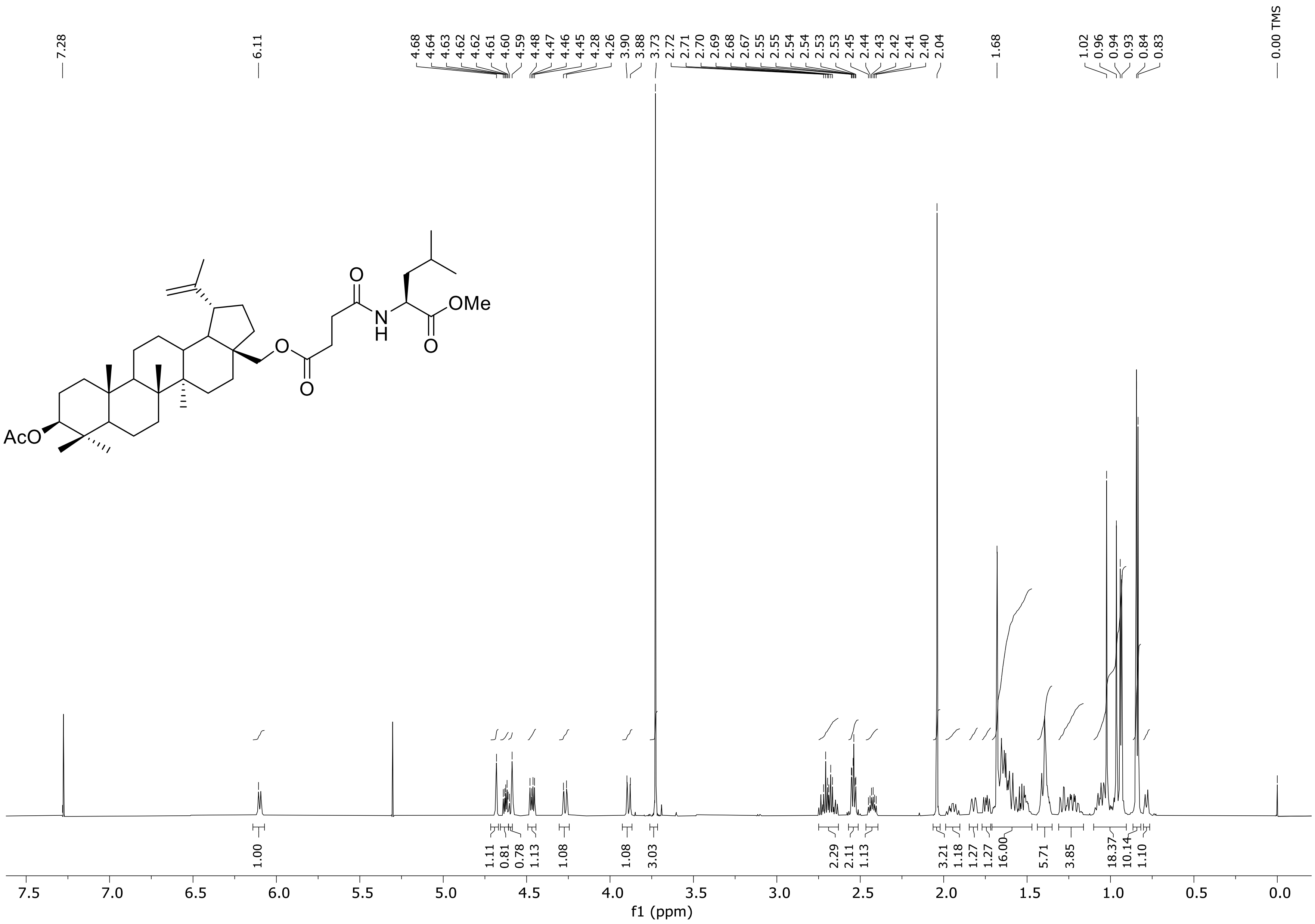

Figure S7. ^{1}H NMR spectrum of 3-OAc-28-O-[Suc-Leu(OMe)]-BN (**3d**); 600 MHz/$CDCl_3$/TMS; δ (ppm).

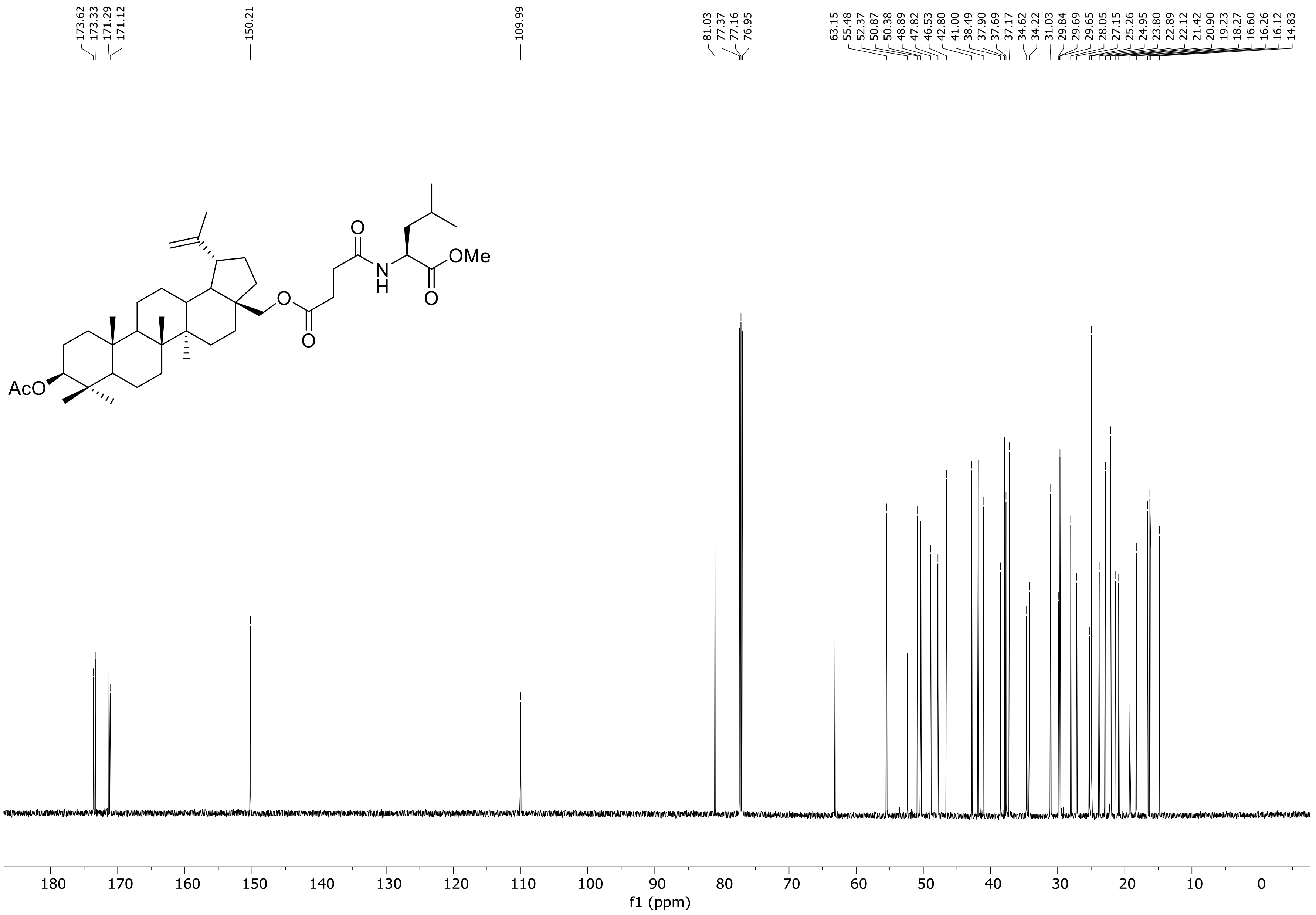

Figure S8. ^{13}C NMR spectrum of 3-OAc-28-O-[Suc-Leu(OMe)]-BN (**3d**); 150 MHz/$CDCl_3$/TMS; δ (ppm).

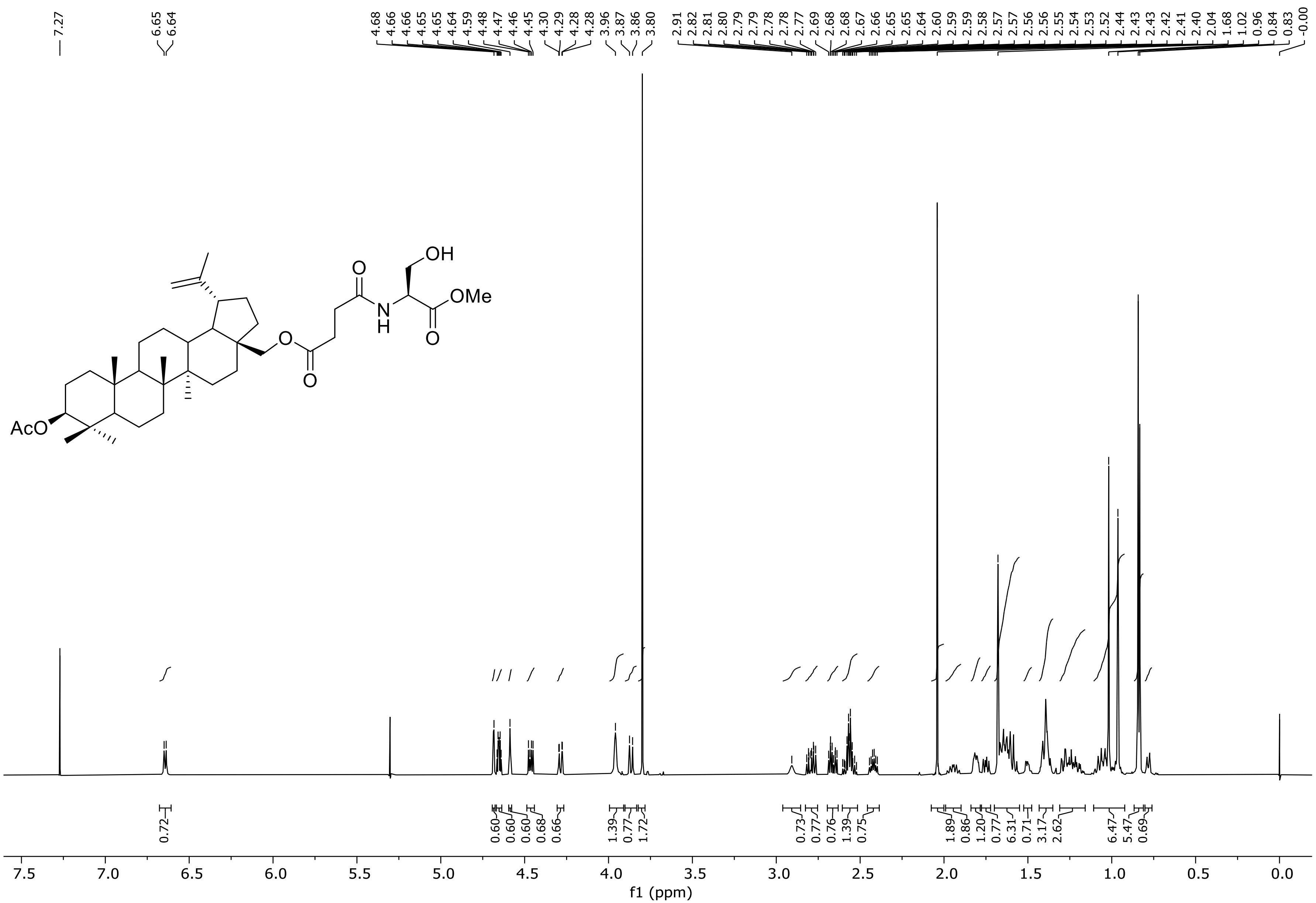

Figure S9. ^{1}H NMR spectrum of 3-OAc-28-O-[Suc-Ser(OMe)]-BN (**3e**); 600 MHz/CDCl$_3$/TMS; δ (ppm).

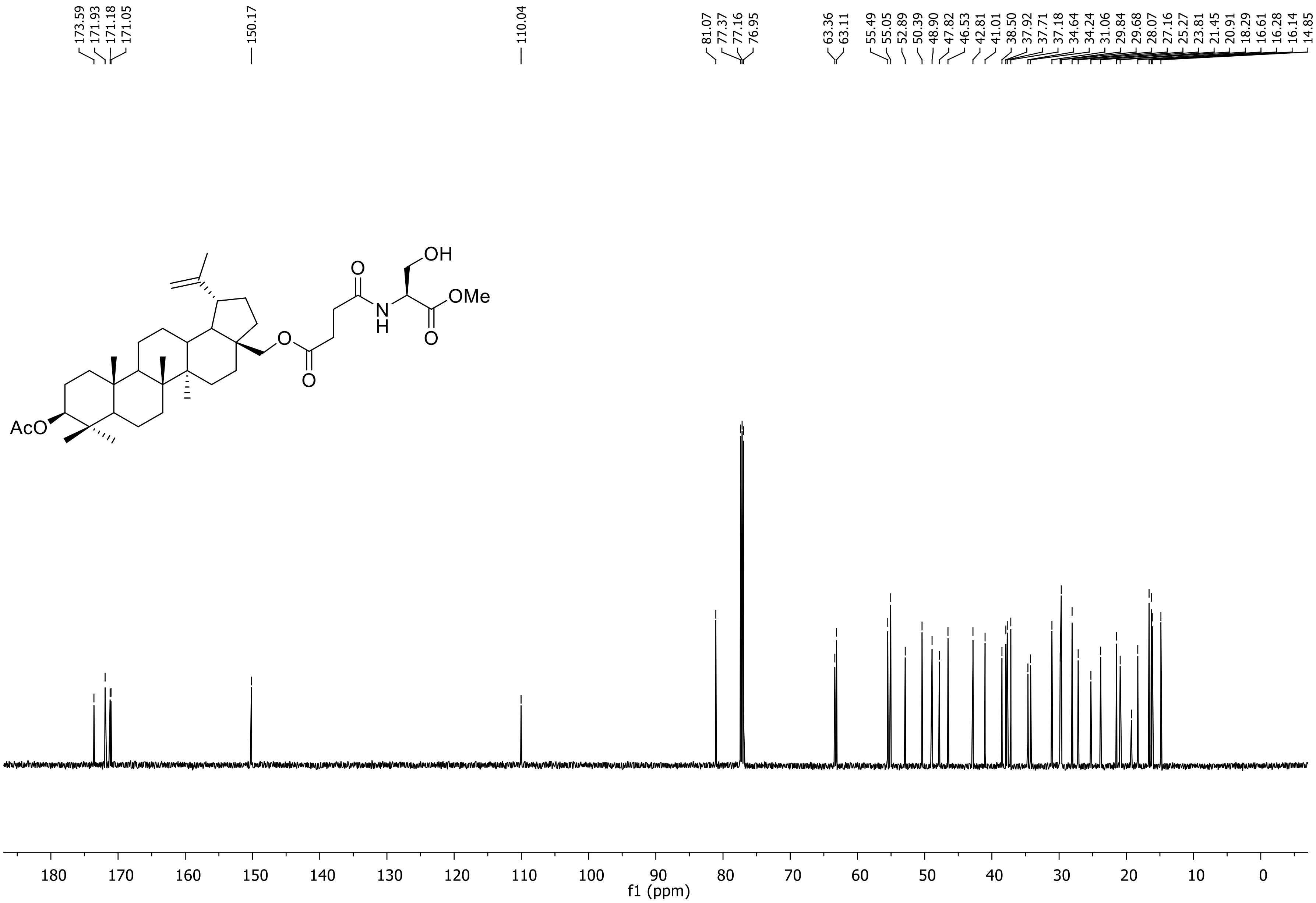

Figure S10. ^{13}C NMR spectrum of 3-OAc-28-O-[Suc-Ser(OMe)]-BN (**3e**); 150 MHz/$CDCl_3$/TMS; *δ* (ppm).

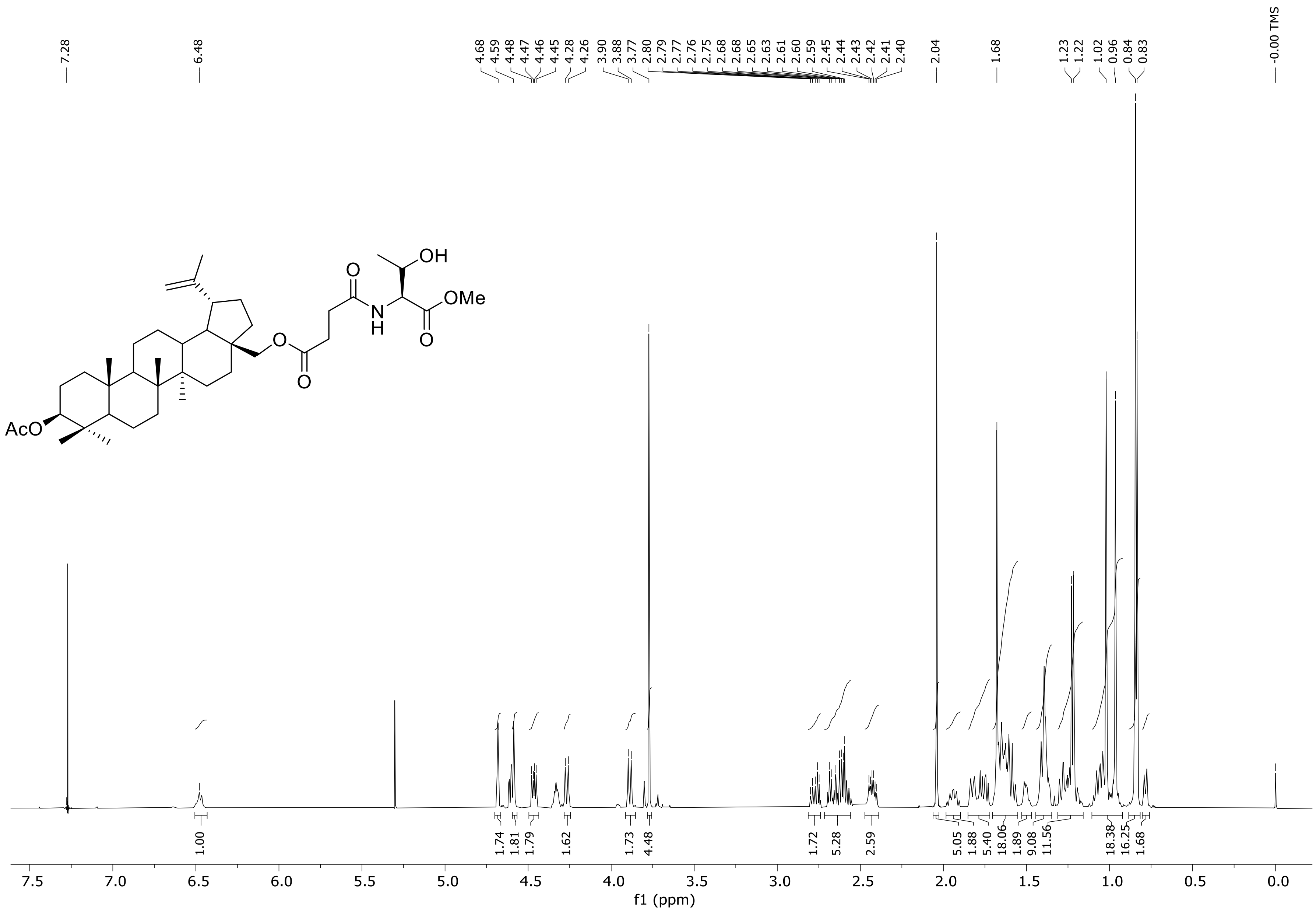

Figure S11. ^{1}H NMR spectrum of 3-OAc-28-O-[Suc-Thr(OMe)]-BN (**3f**); 600 MHz/$CDCl_3$/TMS; δ (ppm).

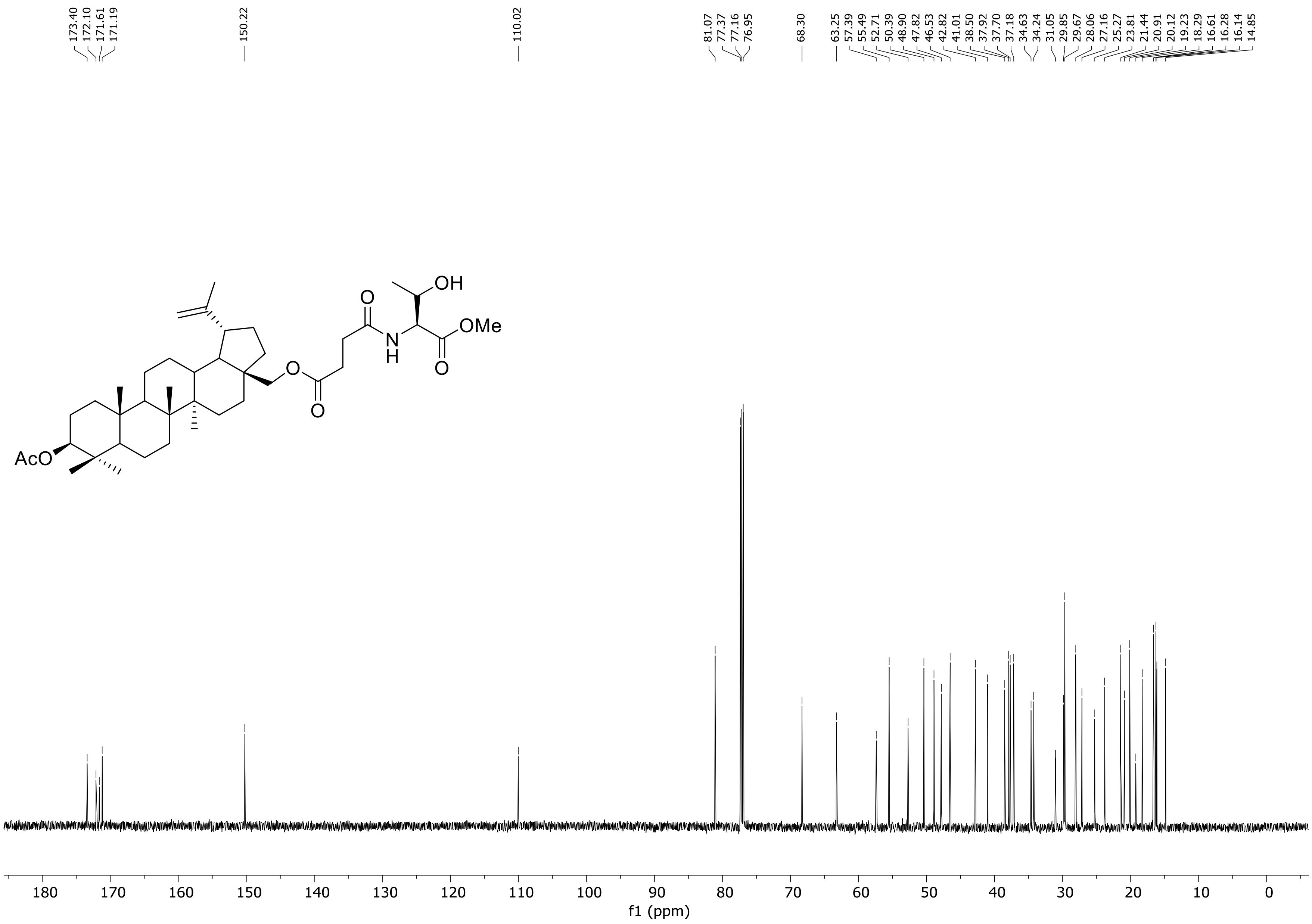

Figure S12. ^{13}C NMR spectrum of 3-OAc-28-O-[Suc-Thr(OMe)]-BN (**3f**); 150 MHz/$CDCl_3$/TMS; δ (ppm).

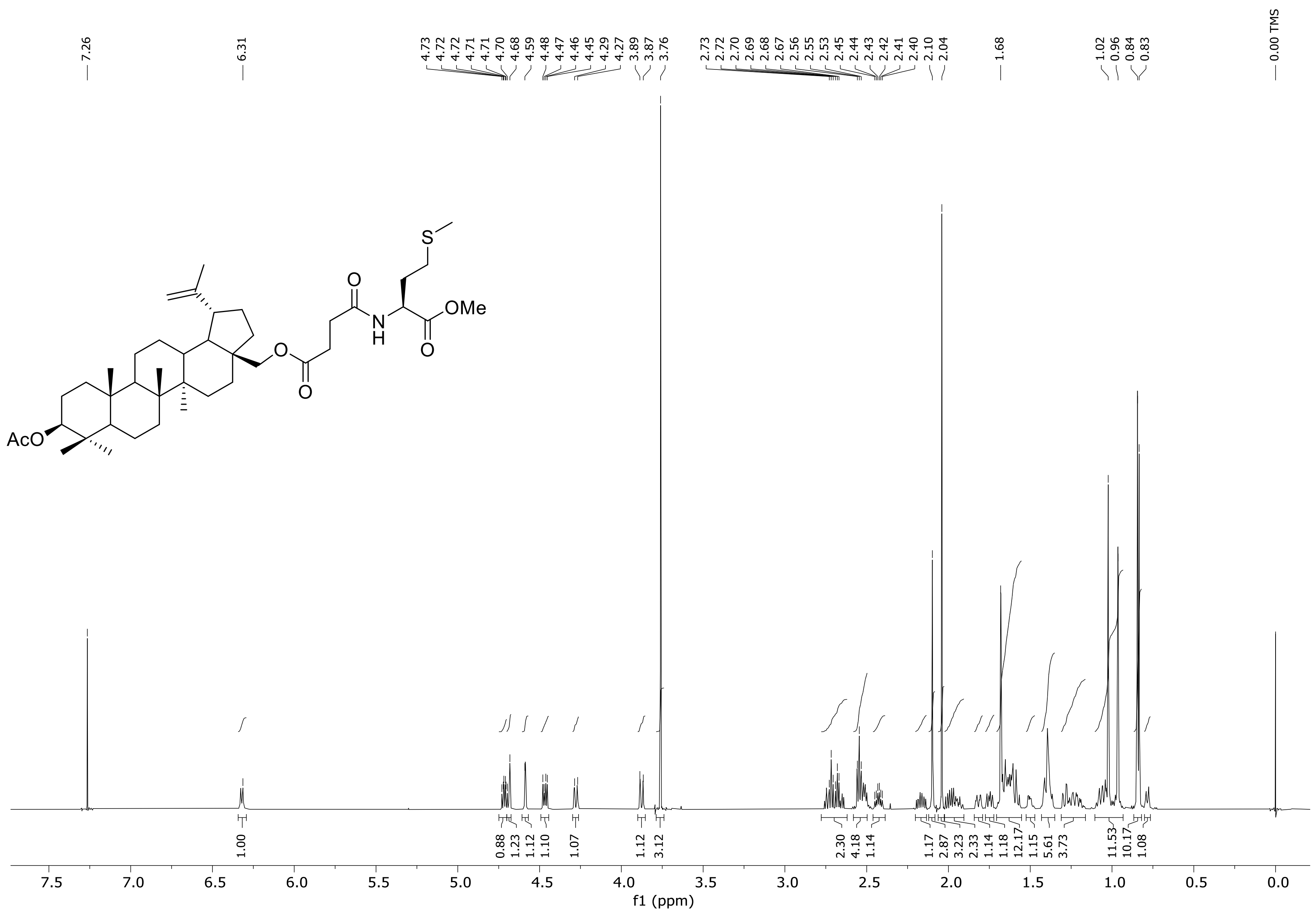

Figure S13. ^{1}H NMR spectrum of 3-OAc-28-O-[Suc-Met(OMe)]-BN (**3g**); 600 MHz/$CDCl_3$/TMS; δ (ppm).

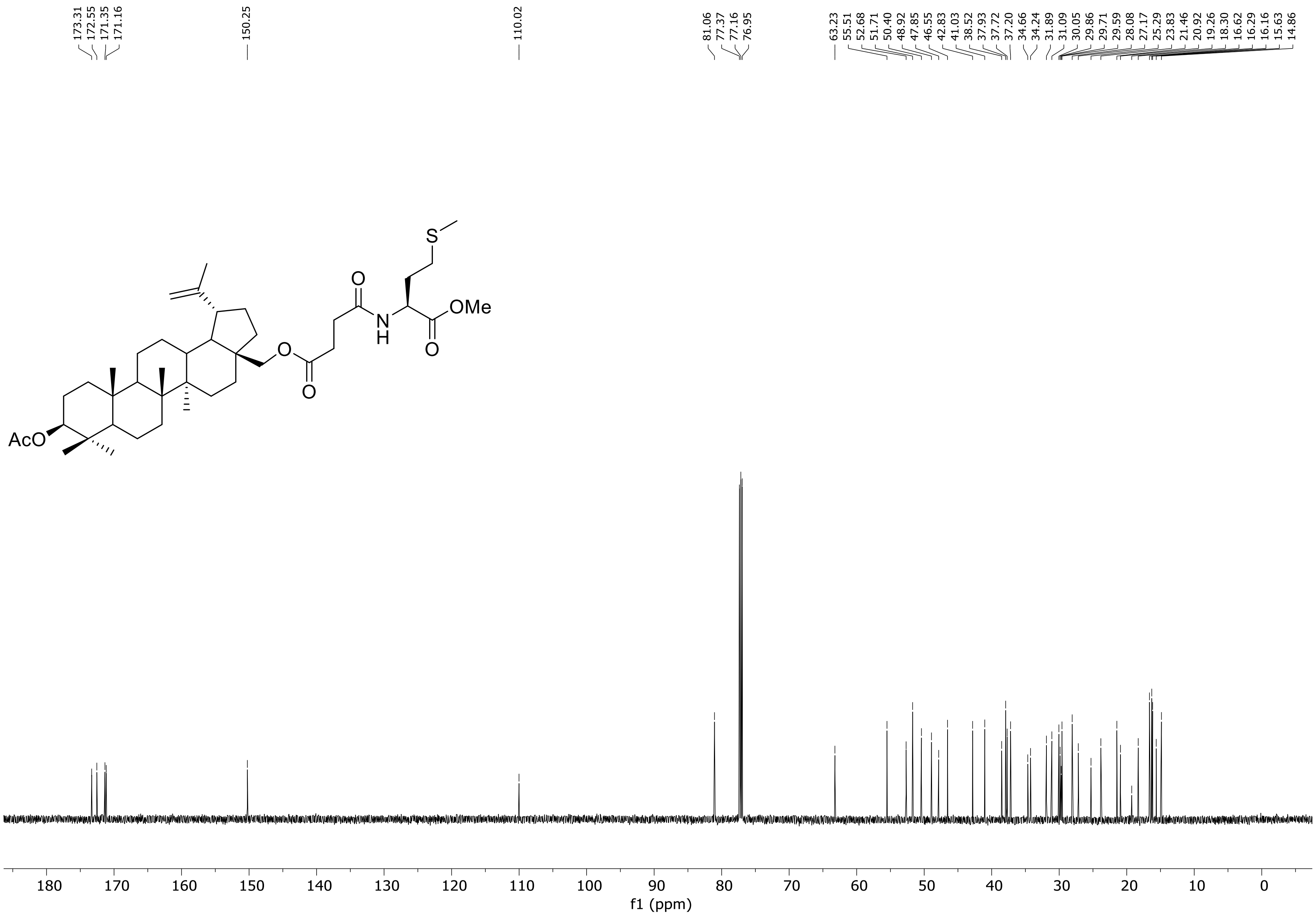

Figure S14. ^{13}C NMR spectrum of 3-OAc-28-O-[Suc-Met(OMe)]-BN (**3g**); 150 MHz/CDCl$_3$/TMS; δ (ppm).

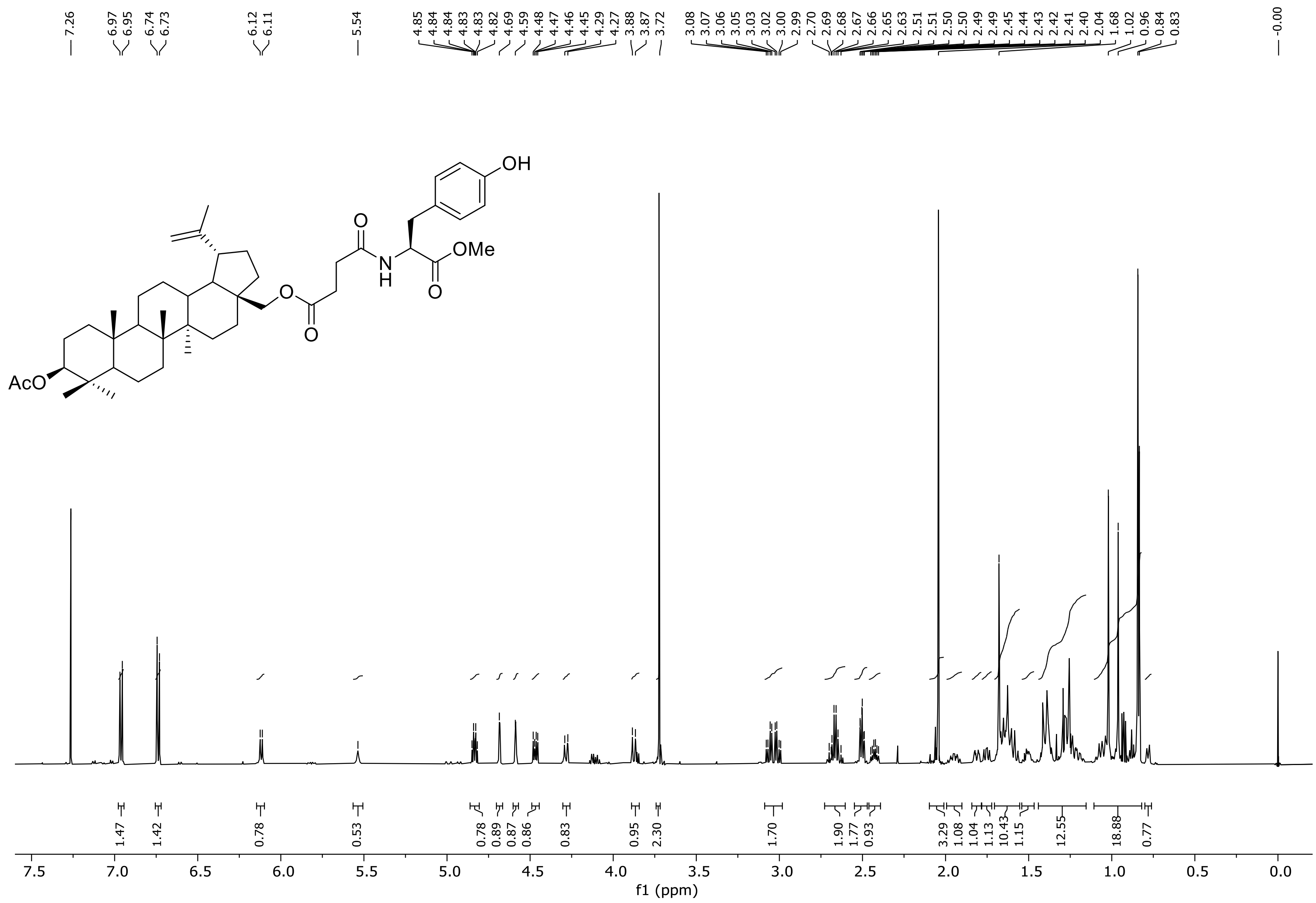

Figure S15. ^{1}H NMR spectrum of 3-OAc-28-O-[Suc-Tyr(OMe)]-BN (**3h**); 600 MHz/$CDCl_3$/TMS; δ (ppm).

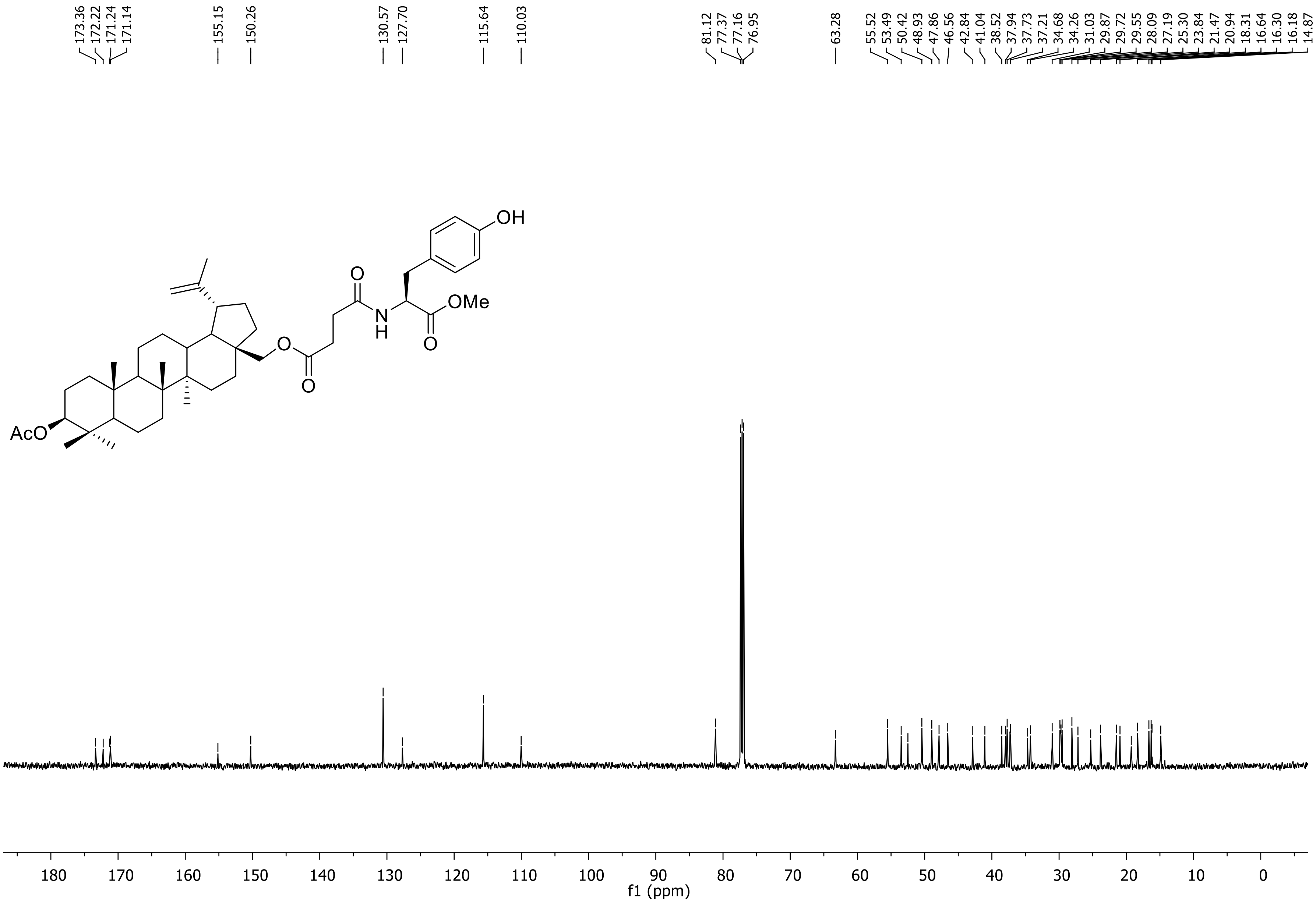

Figure S16. ^{13}C NMR spectrum of 3-OAc-28-O-[Suc-Tyr(OMe)]-BN (**3h**); 150 MHz/$CDCl_3$/TMS; δ (ppm).

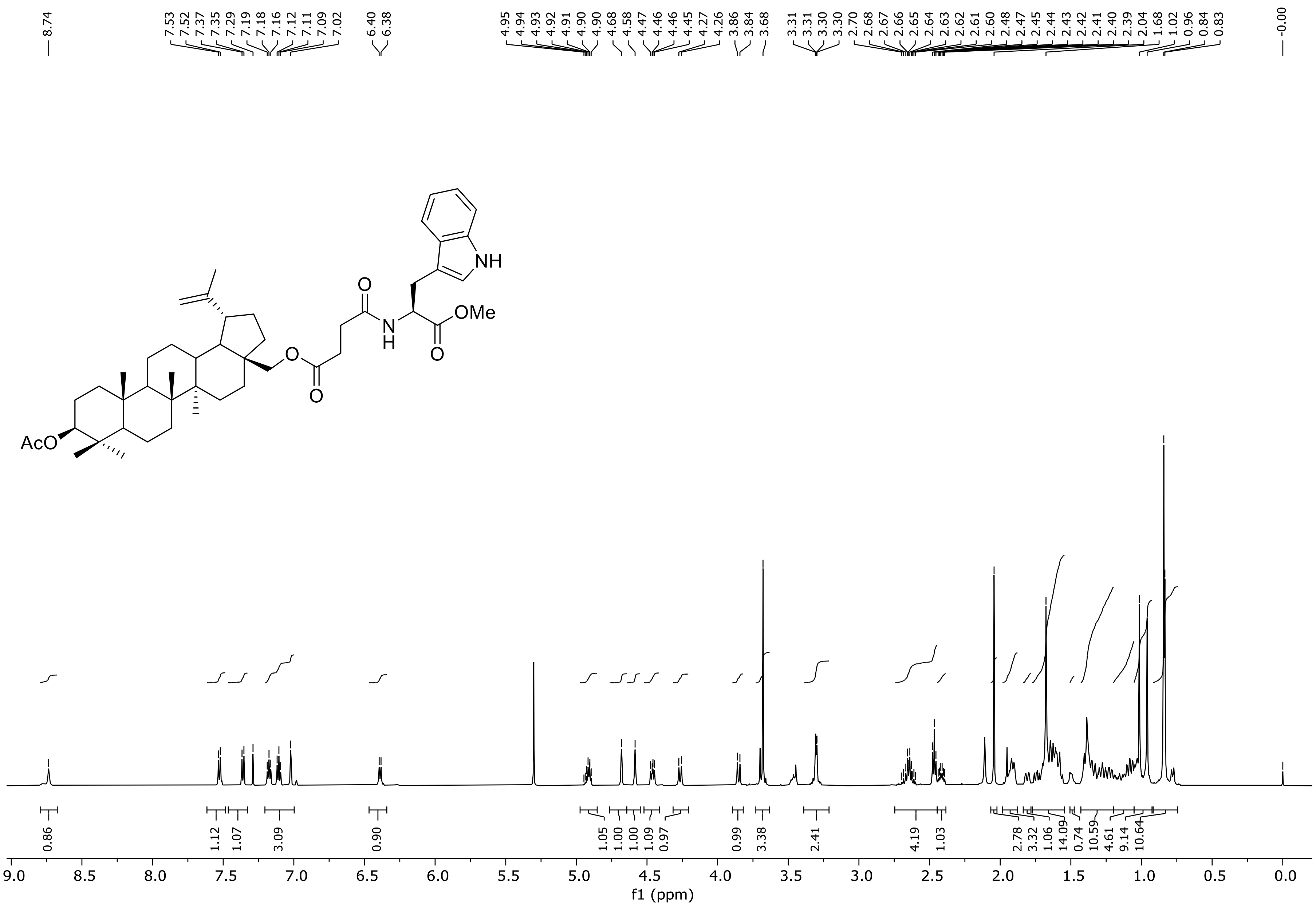

Figure S17. ^{1}H NMR spectrum of 3-OAc-28-O-[Suc-Trp(OMe)]-BN (**3i**); 600 MHz/$CDCl_3$/TMS; δ (ppm).

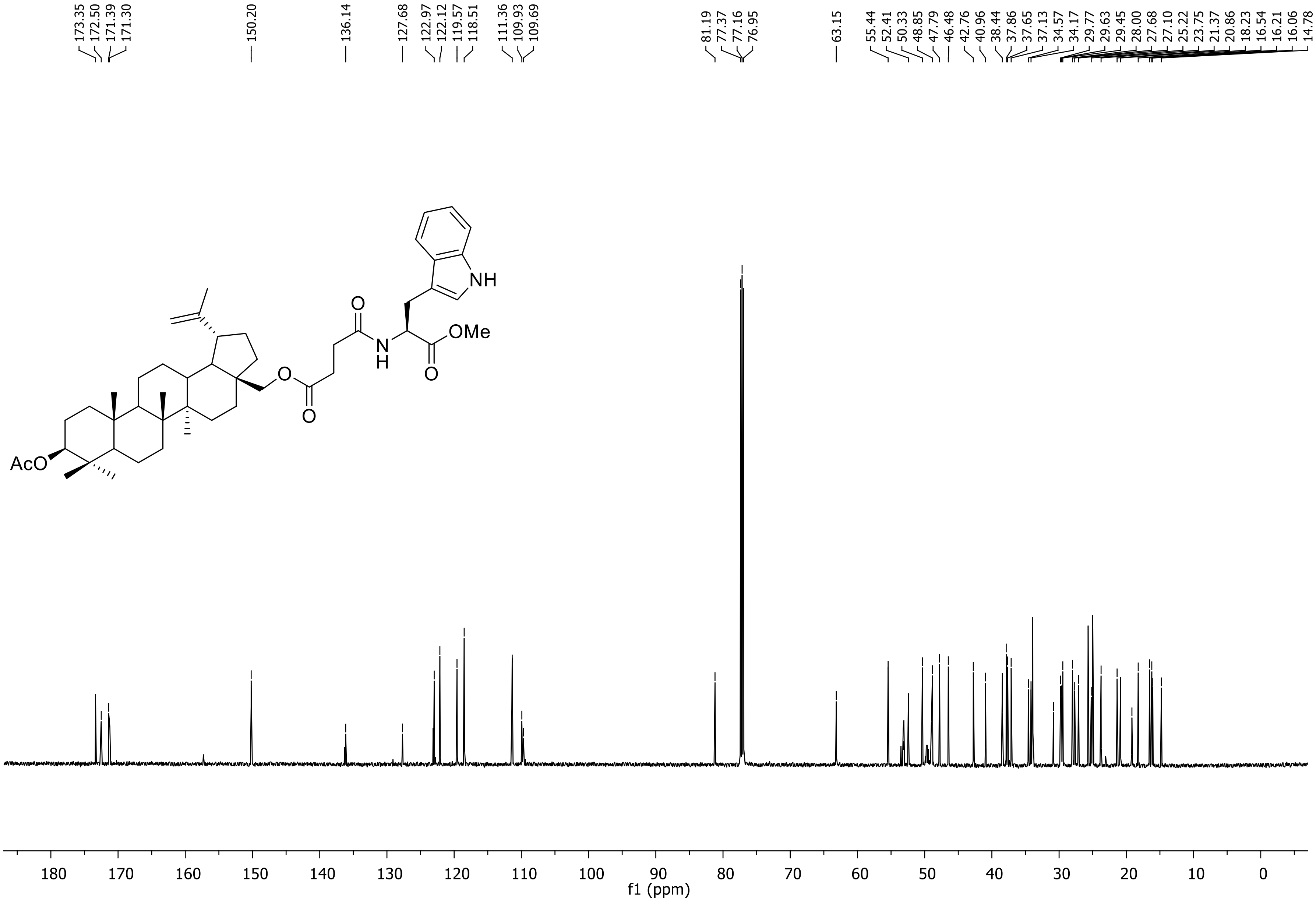

Figure S18. ^{13}C NMR spectrum of 3-OAc-28-O-[Suc-Trp(OMe)]-BN (**3i**); 150 MHz/$CDCl_3$/TMS; δ (ppm).

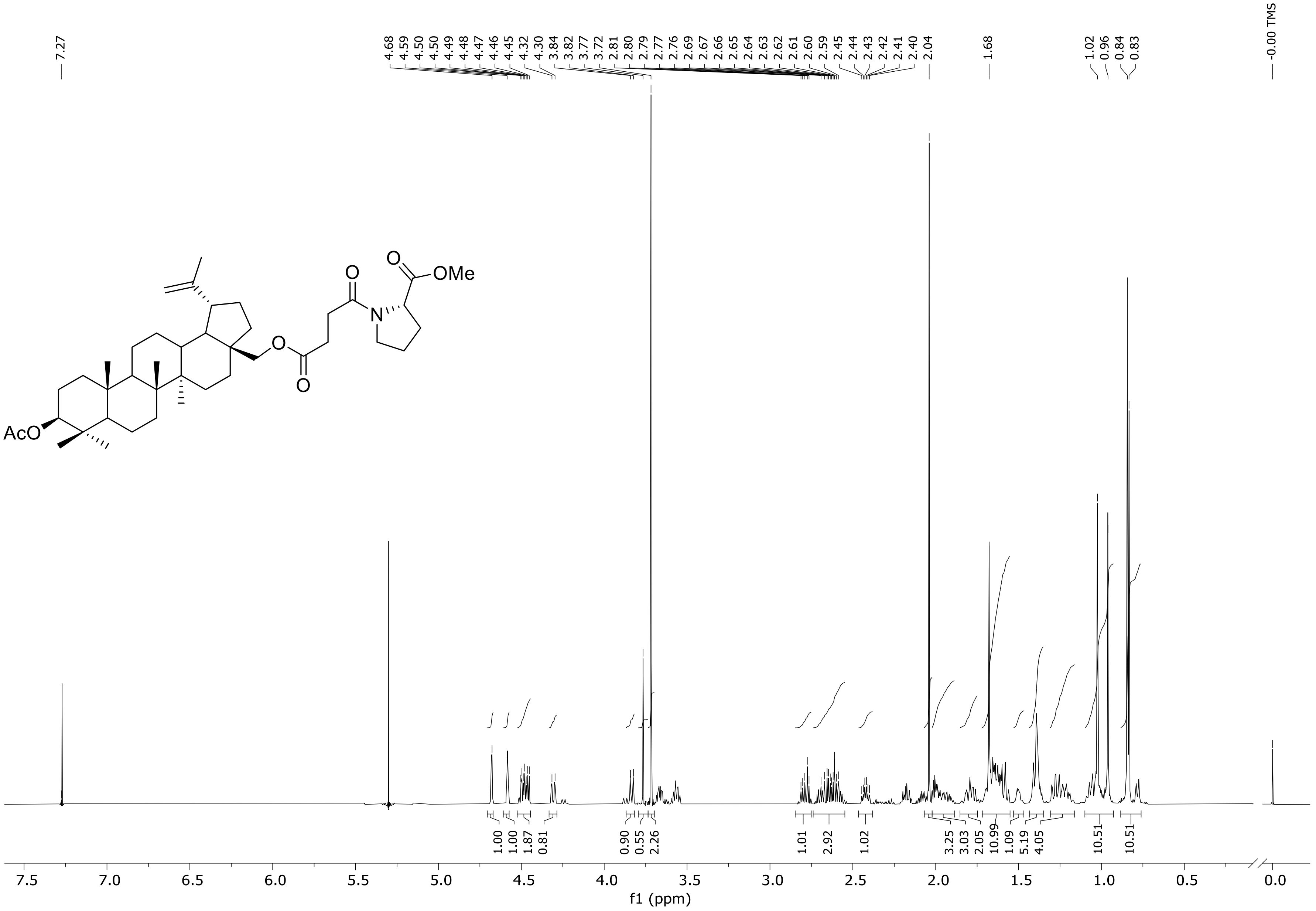

Figure S19. ^{1}H NMR spectrum of 3-OAc-28-O-[Suc-Pro(OMe)]-BN (**3j**); 600 MHz/$CDCl_3$/TMS; δ (ppm).

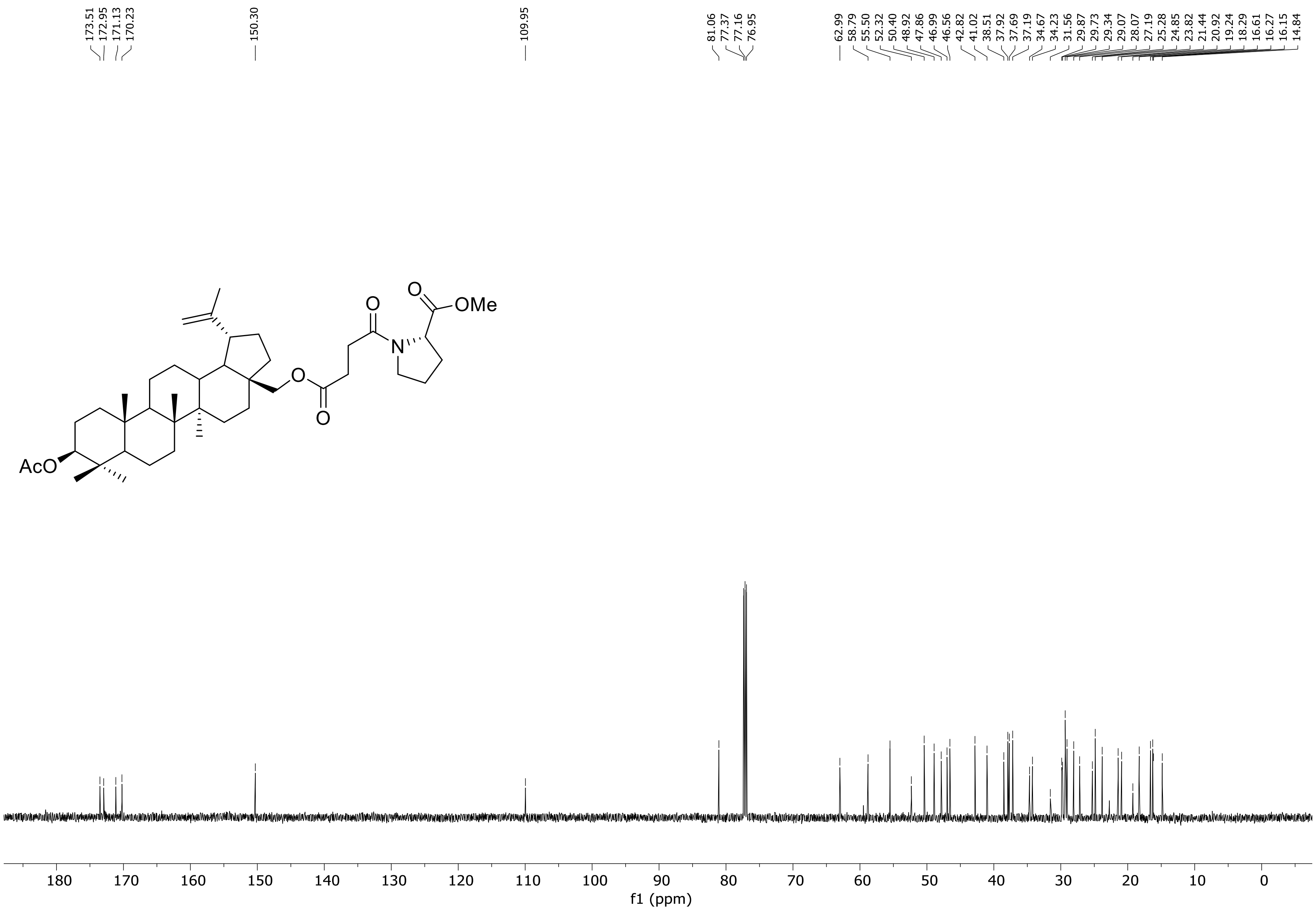

Figure S20. ^{13}C NMR spectrum of 3-OAc-28-O-[Suc-Pro(OMe)]-BN (**3j**); 150 MHz/CDCl$_3$/TMS; δ (ppm).

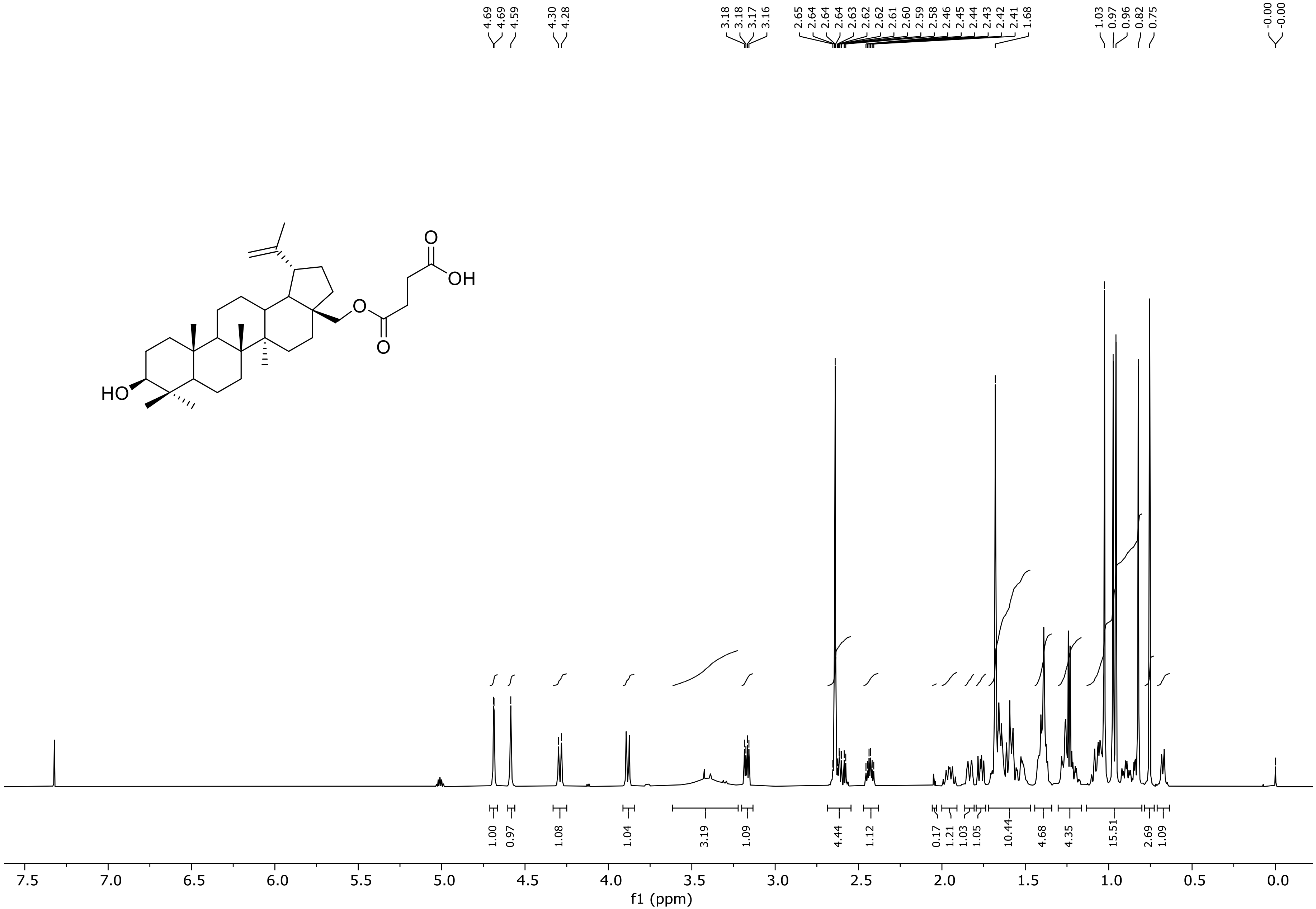

Figure S21. ^{1}H NMR spectrum of 28-O'-(3'-carboxypropanoyl)betulin (**4**); 600 MHz/$CDCl_3$/TMS; δ (ppm).

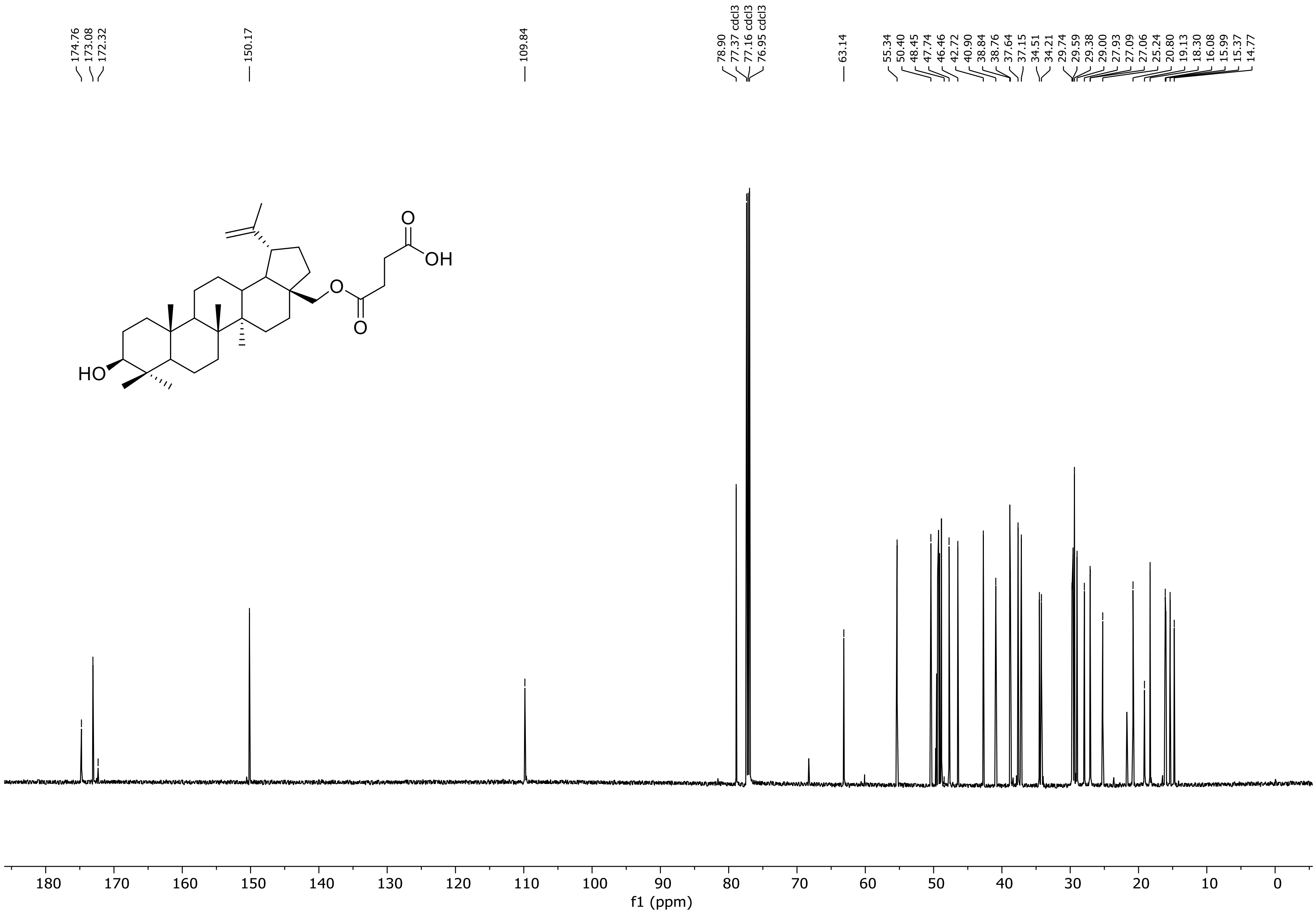

Figure S22. ^{13}C NMR spectrum of 28-O’-(3'-carboxypropanoyl)betulin (**4**); 150 MHz/$CDCl_3$/TMS; δ (ppm).

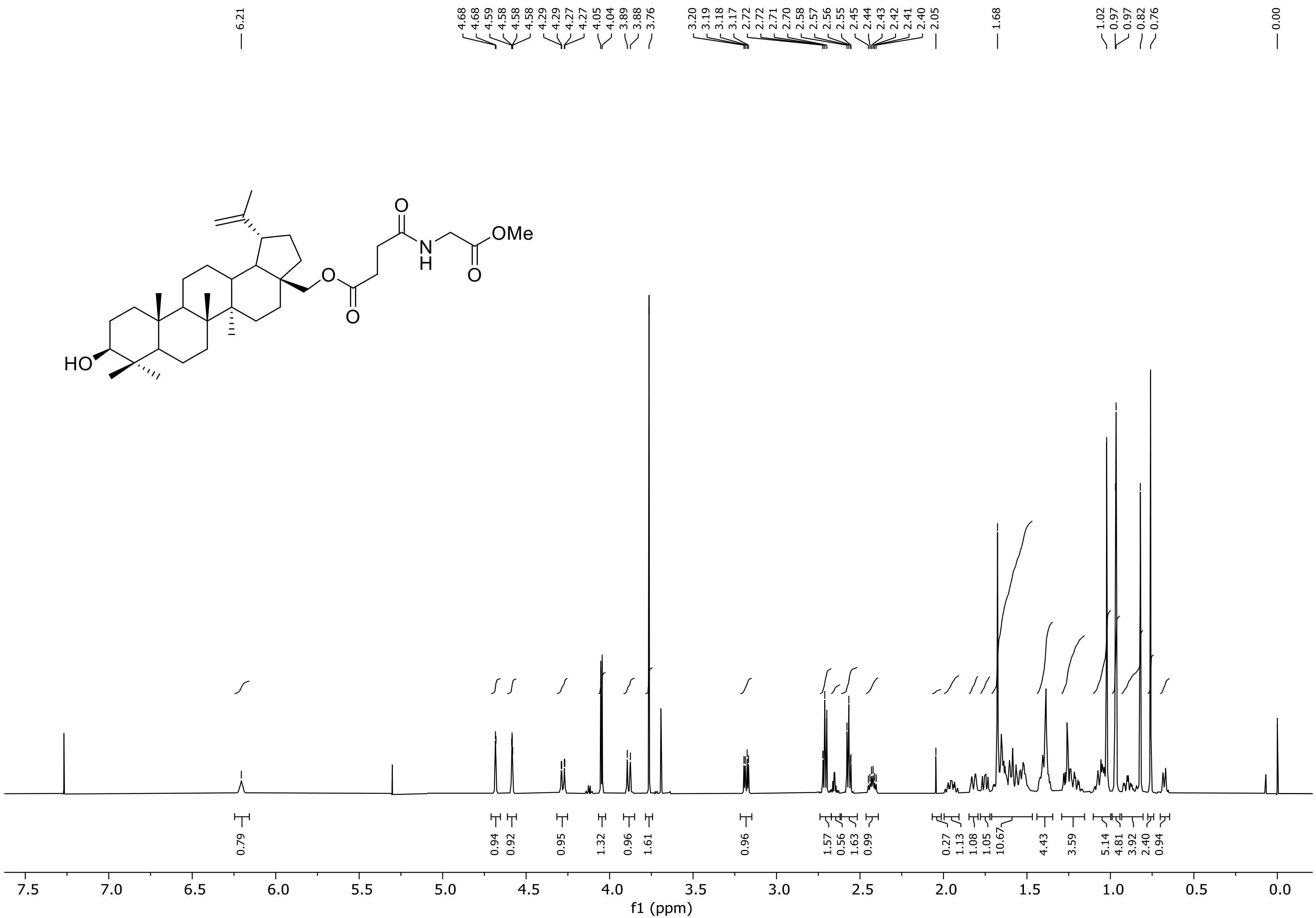

Figure S23. ^{1}H NMR spectrum of 3-OH-28-O-[Suc-Gly(OMe)]-BN (**5a**); 600 MHz/$CDCl_3$/TMS; δ (ppm).

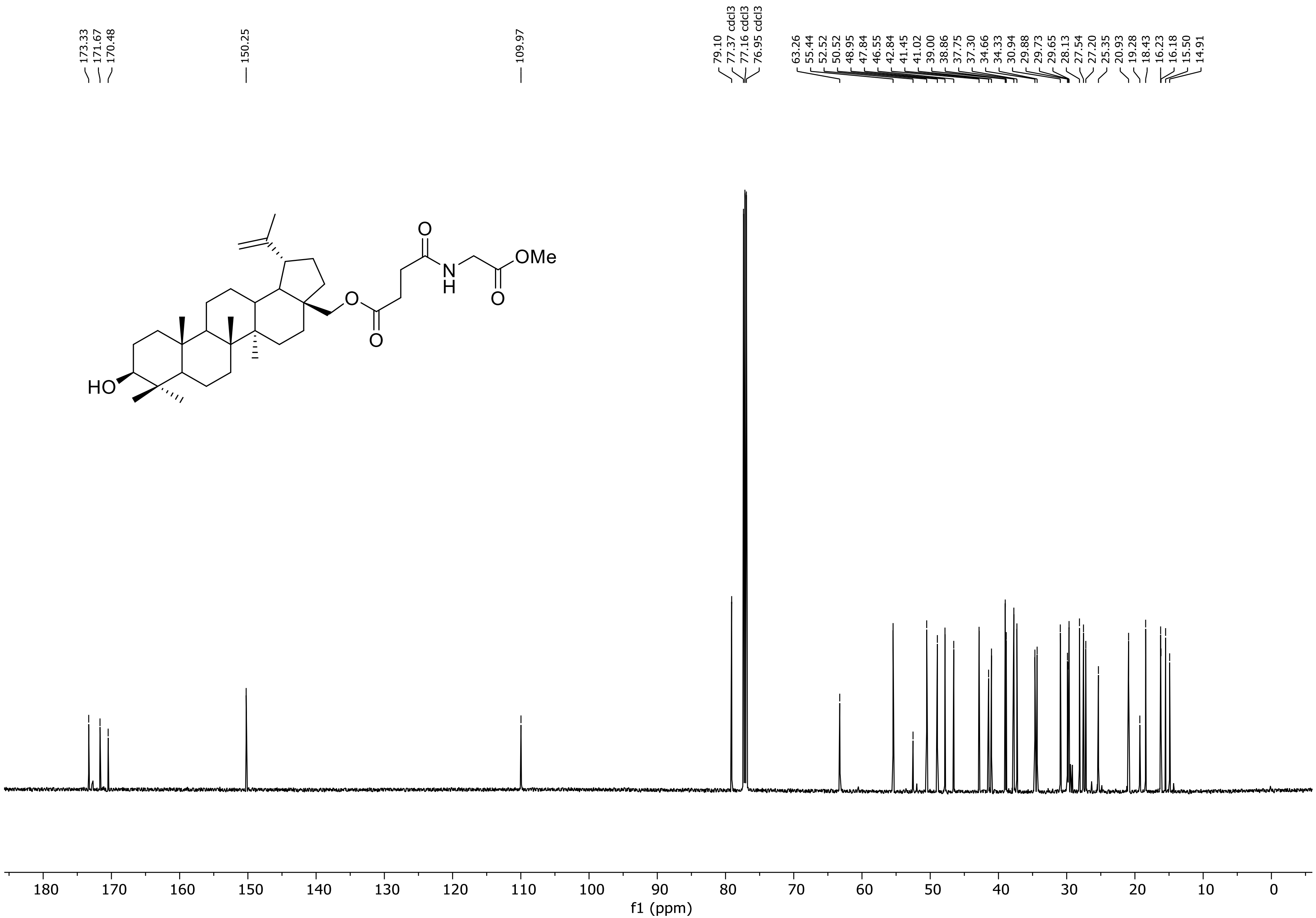

Figure S24. ^{13}C NMR spectrum of 3-OH-28-O-[Suc-Gly(OMe)]-BN (**5a**); 150 MHz/$CDCl_3$/TMS; δ (ppm).

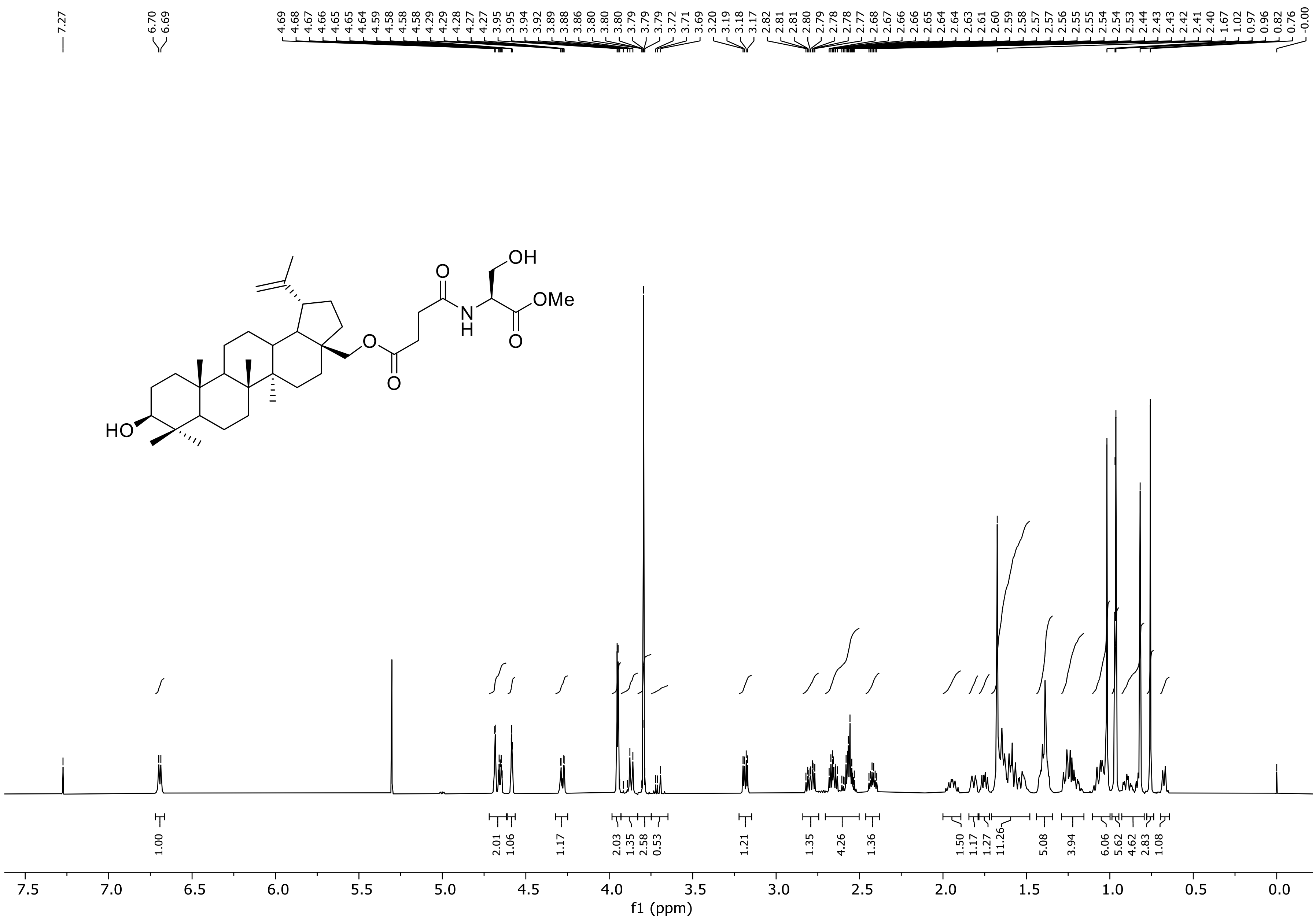

Figure S25. ^{1}H NMR spectrum of 3-OH-28-O-[Suc-Ser(OMe)]-BN (**5b**); 600 MHz/$CDCl_3$/TMS; δ (ppm).

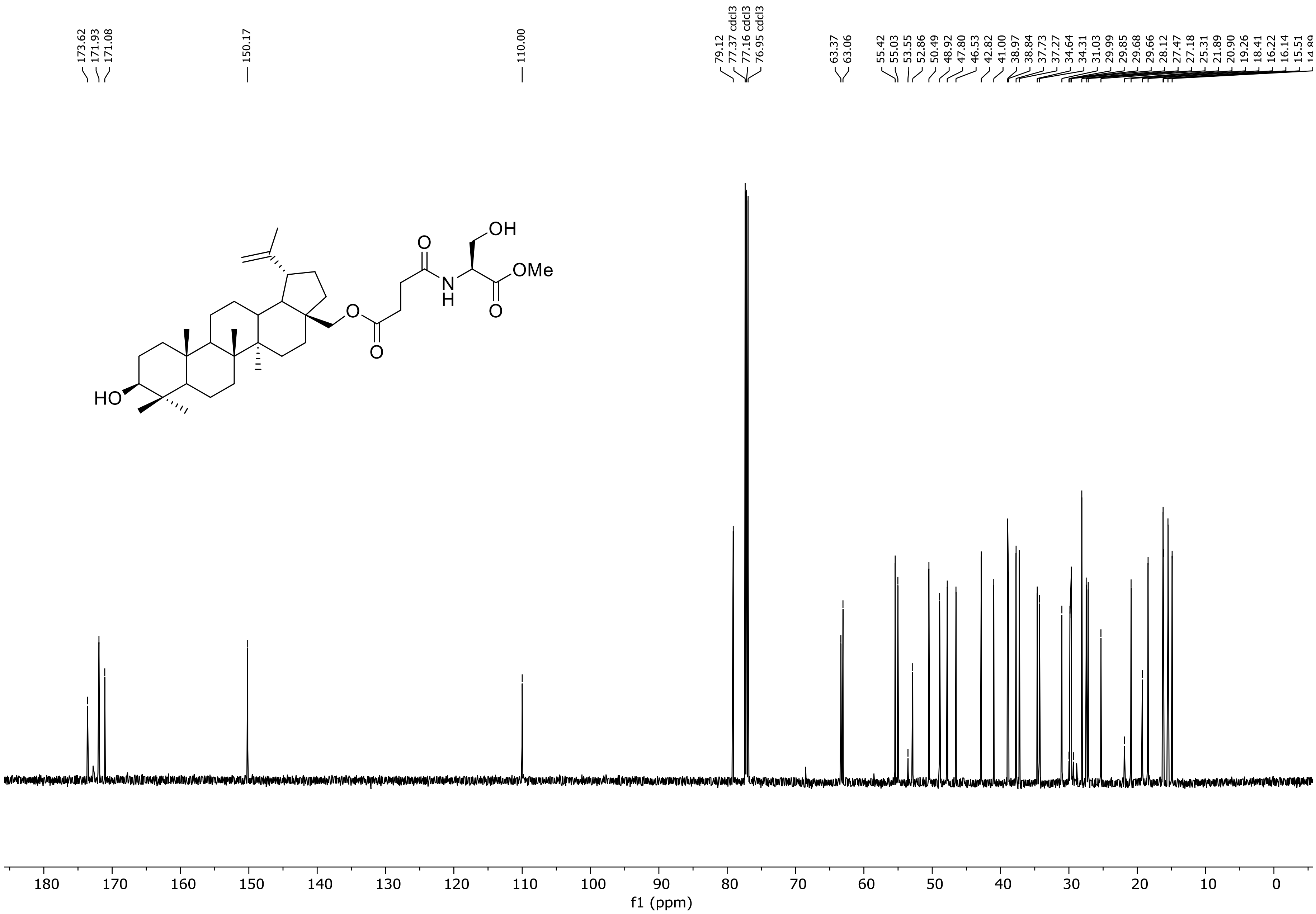

Figure S26. ^{13}C NMR spectrum of 3-OH-28-O-[Suc-Ser(OMe)]-BN (**5b**); 150 MHz/$CDCl_3$/TMS; *δ* (ppm).

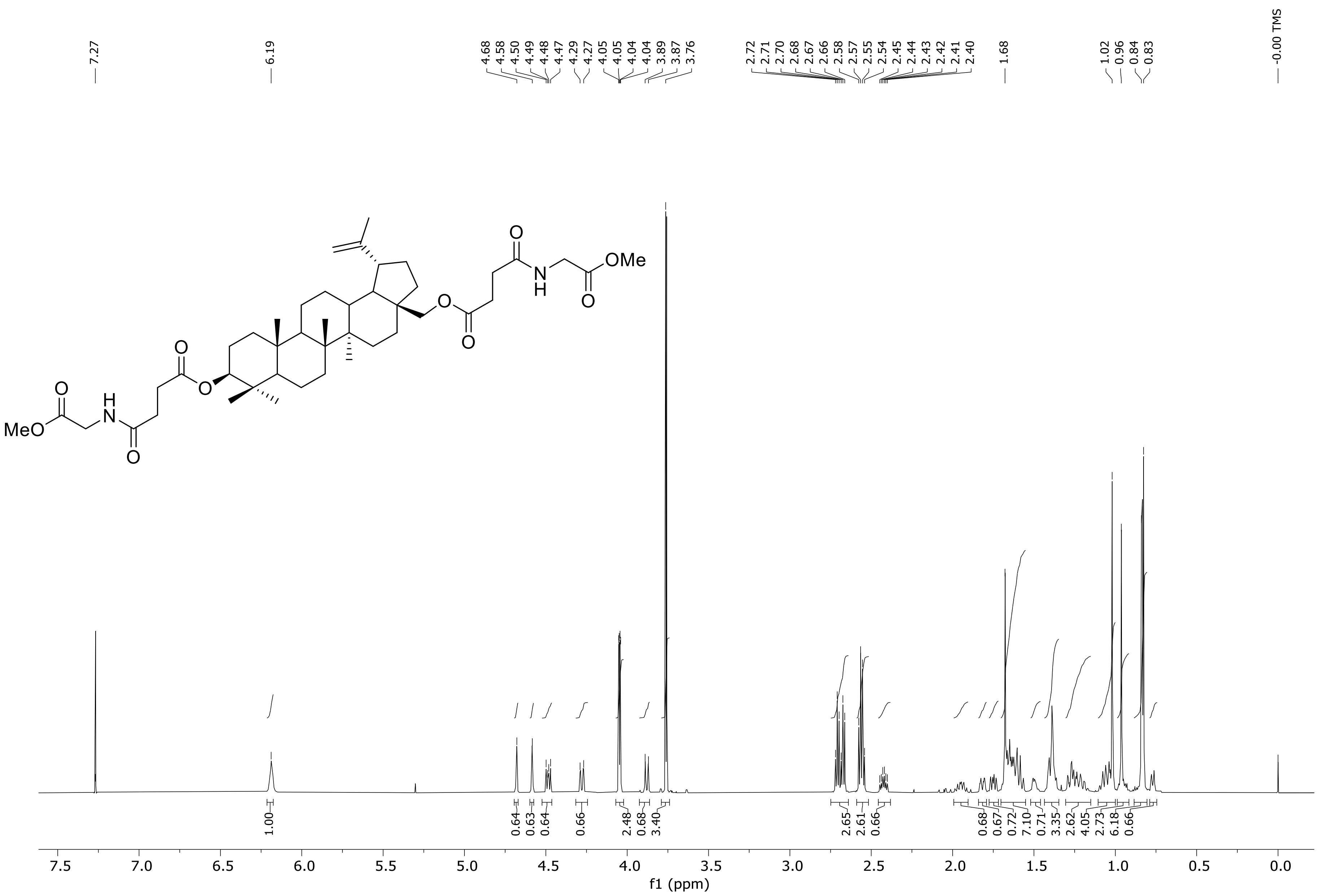

Figure S27. ¹H NMR spectrum of 3,28-*bis*[O-Suc-Gly(OMe)]-BN (**7a**); 600 MHz/$CDCl_3$/TMS; δ (ppm).

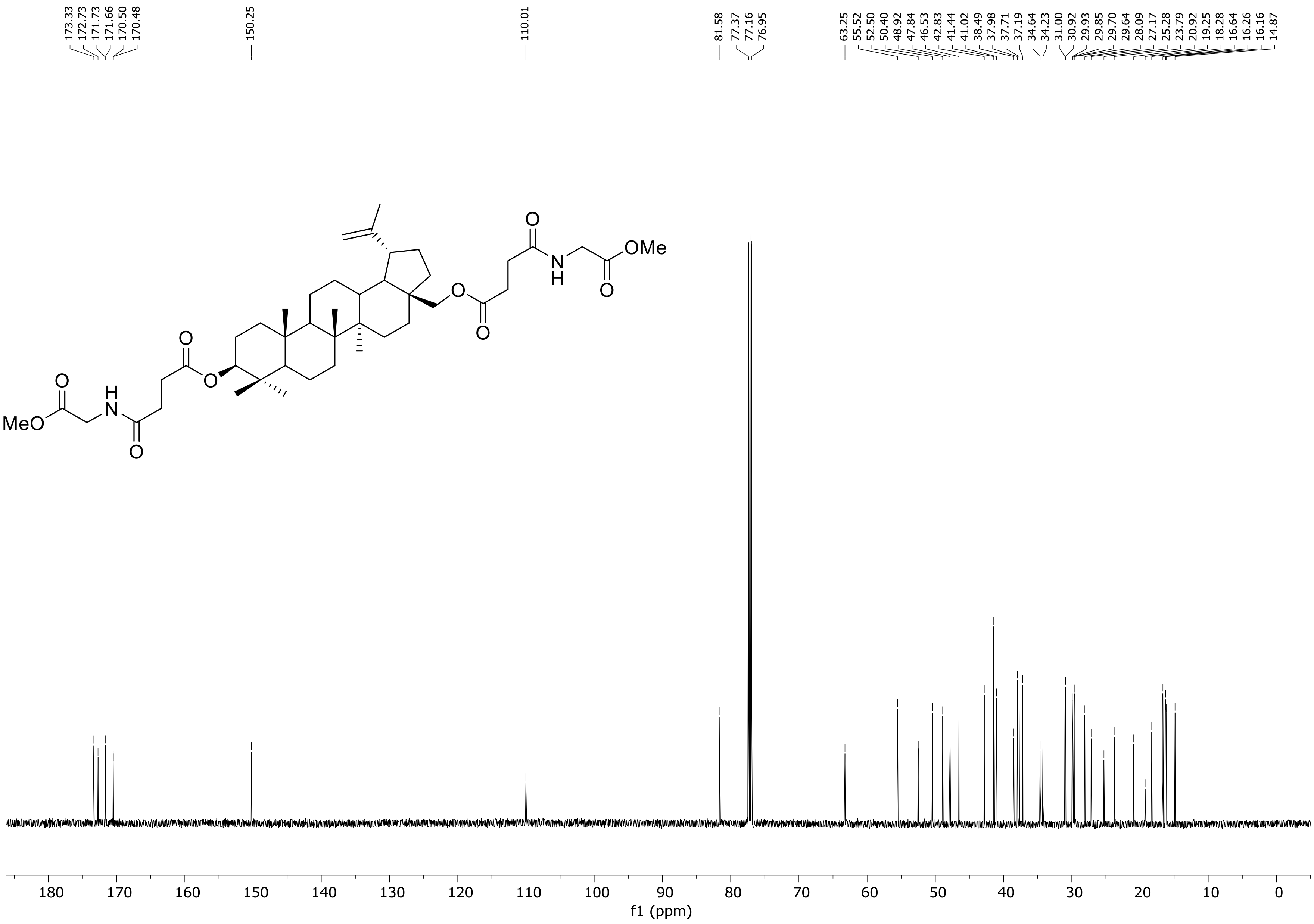

Figure S28. ^{13}C NMR spectrum of 3,28-*bis*[O-Suc-Gly(OMe)]-BN (**7a**); 150 MHz/$CDCl_3$/TMS; δ (ppm).

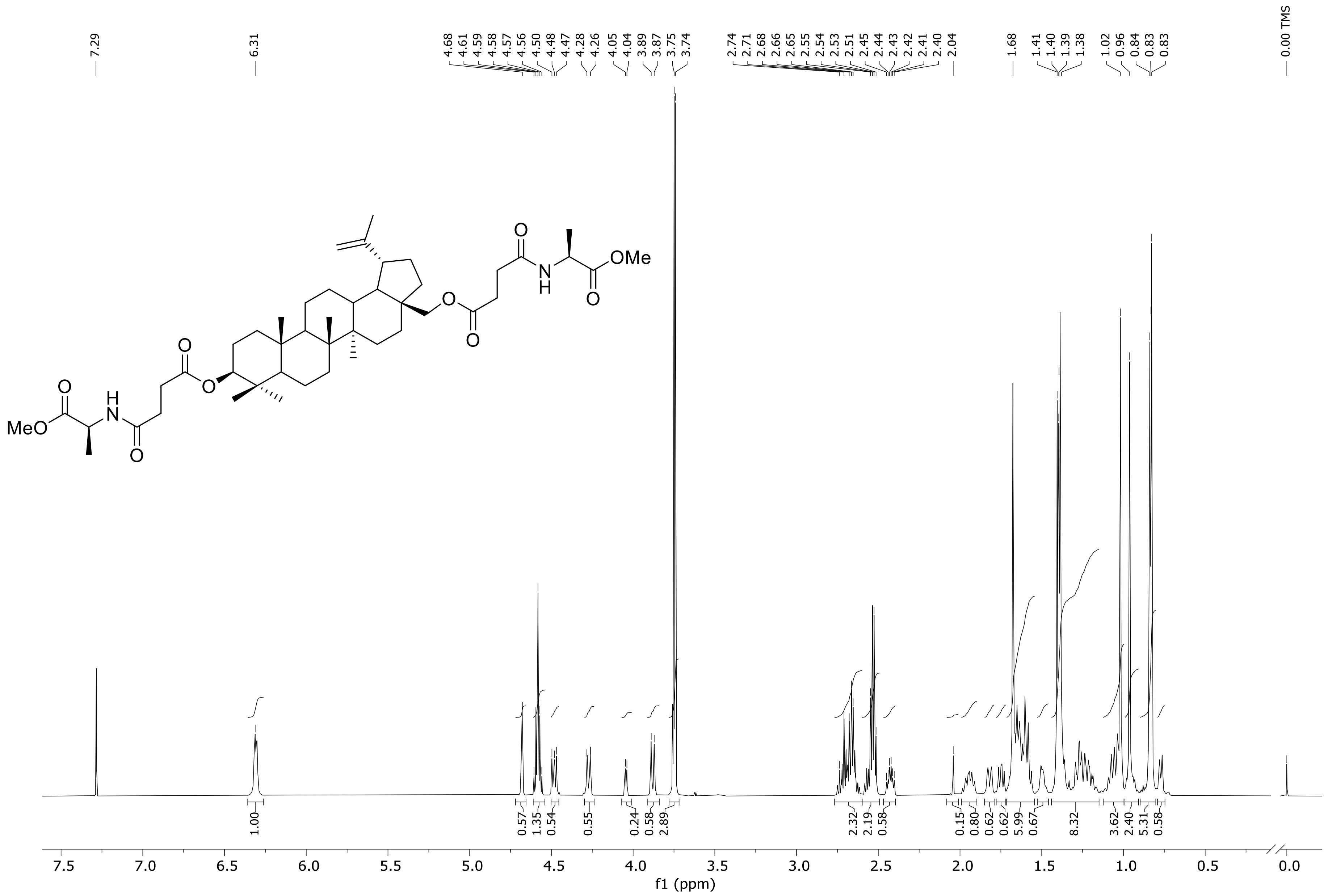

Figure S29. ^{1}H NMR spectrum of 3,28-*bis*[O-Suc-Ala(OMe)]-BN (**7b**); 600 MHz/$CDCl_3$/TMS; δ (ppm).

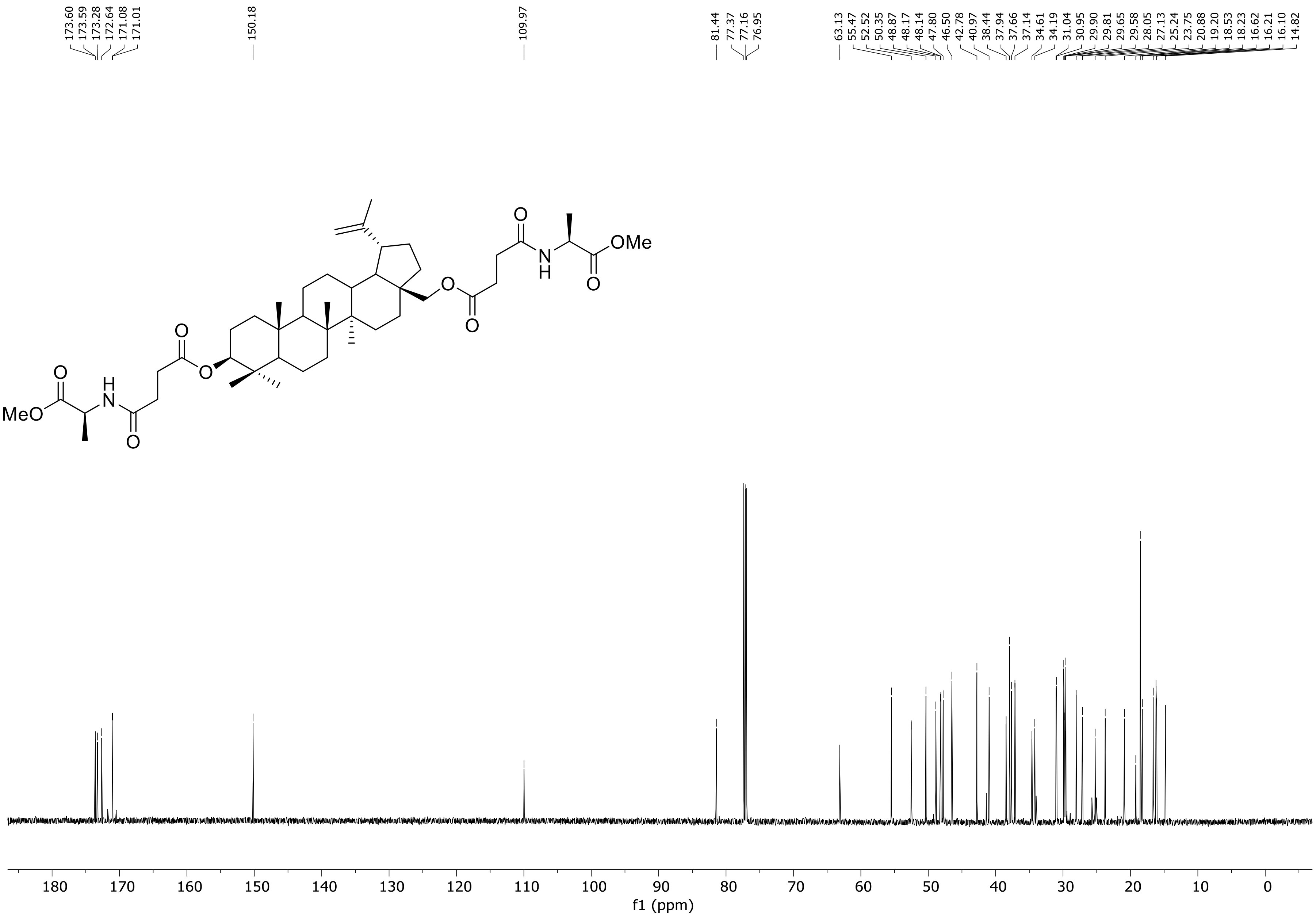

Figure S30. ^{13}C NMR spectrum of 3,28-*bis*[O-Suc-Ala(OMe)]-BN (**7b**); 150 MHz/$CDCl_3$/TMS; δ (ppm).

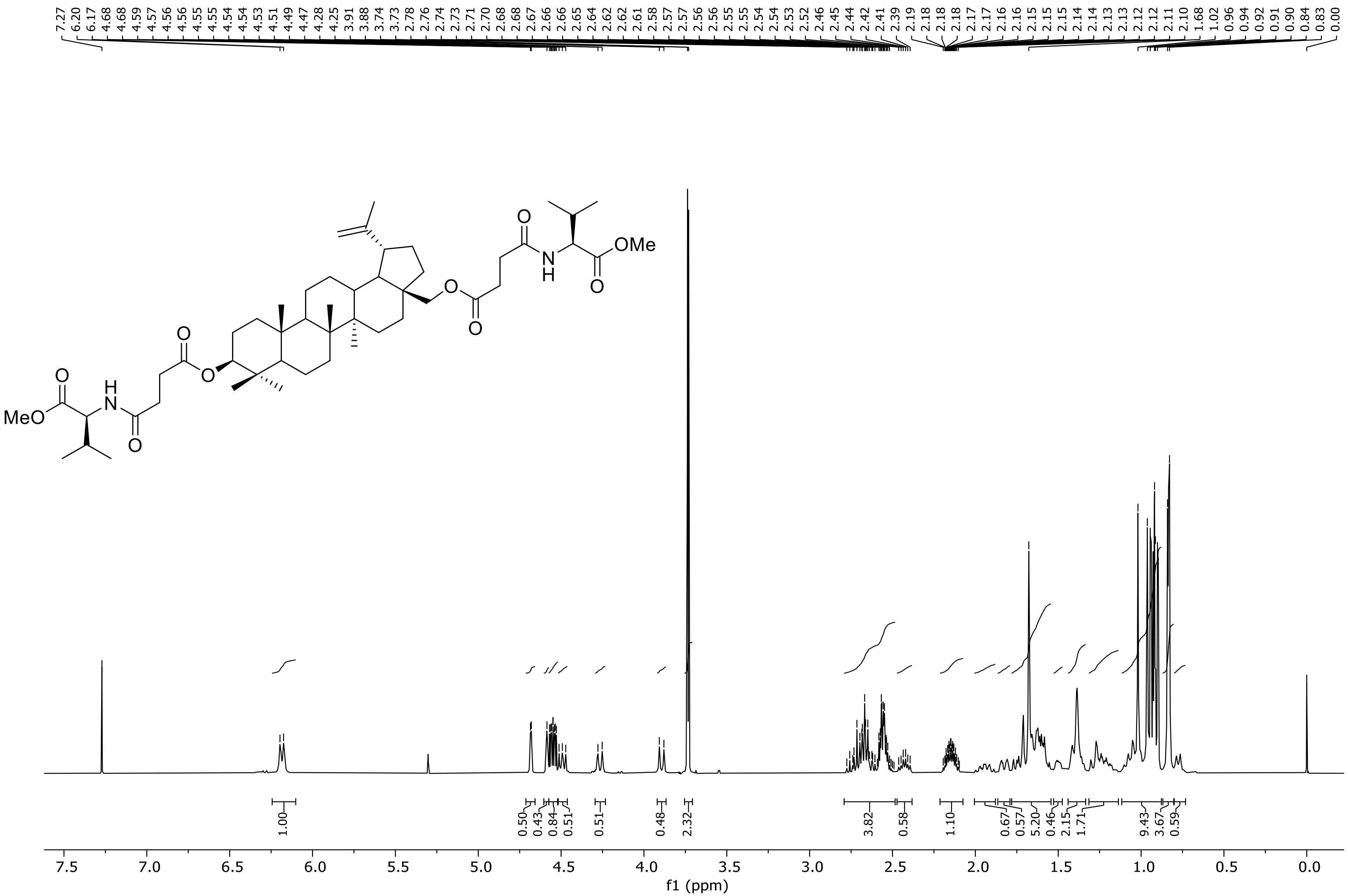

Figure S31. ^{1}H NMR spectrum of 3,28-*bis*[O-Suc-Val(OMe)]-BN (**7c**); 600 MHz/$CDCl_3$/TMS; δ (ppm).

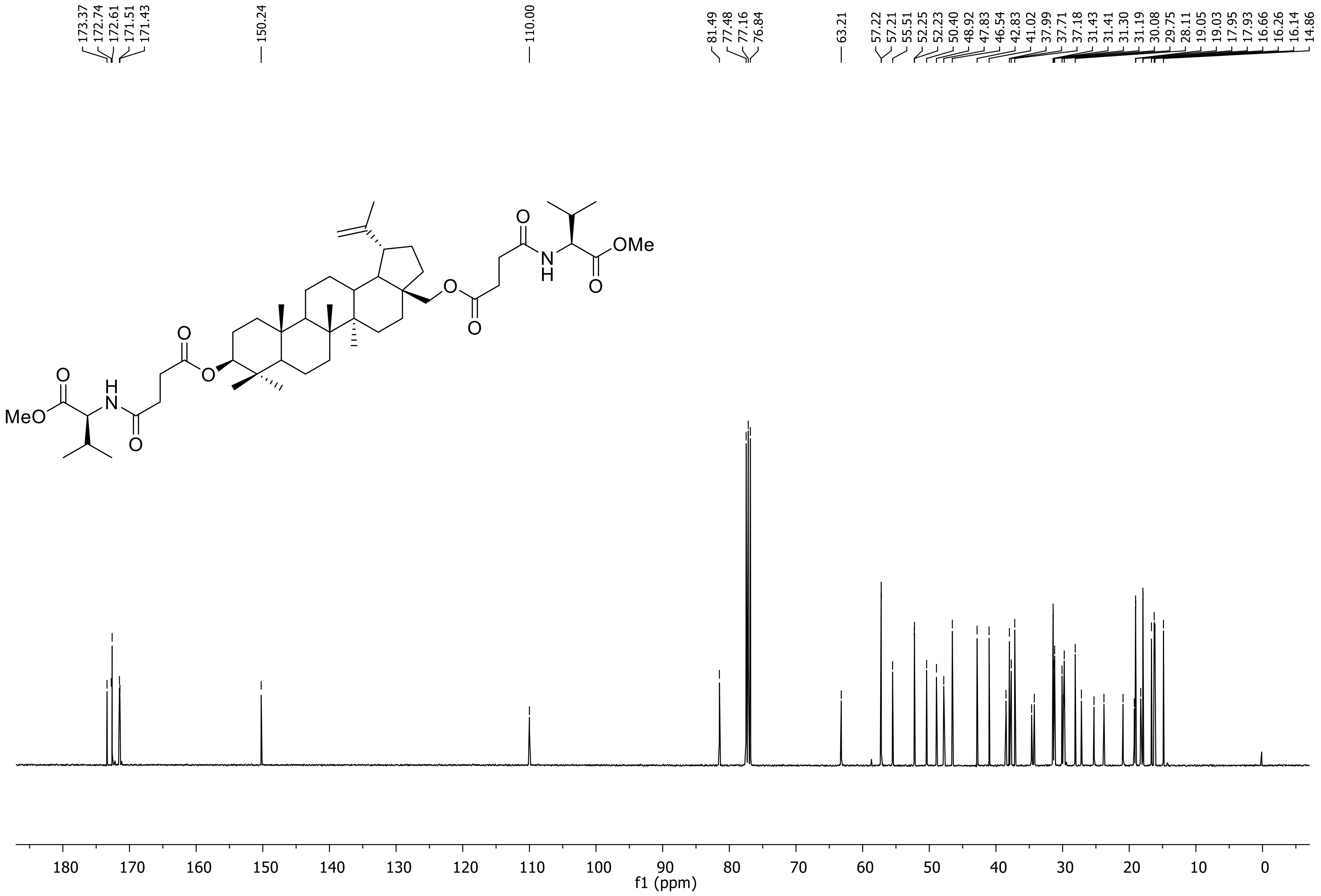

Figure S32. ^{13}C NMR spectrum of 3,28-*bis*[O-Suc-Val(OMe)]-BN (**7c**); 150 MHz/$CDCl_3$/TMS; δ (ppm).

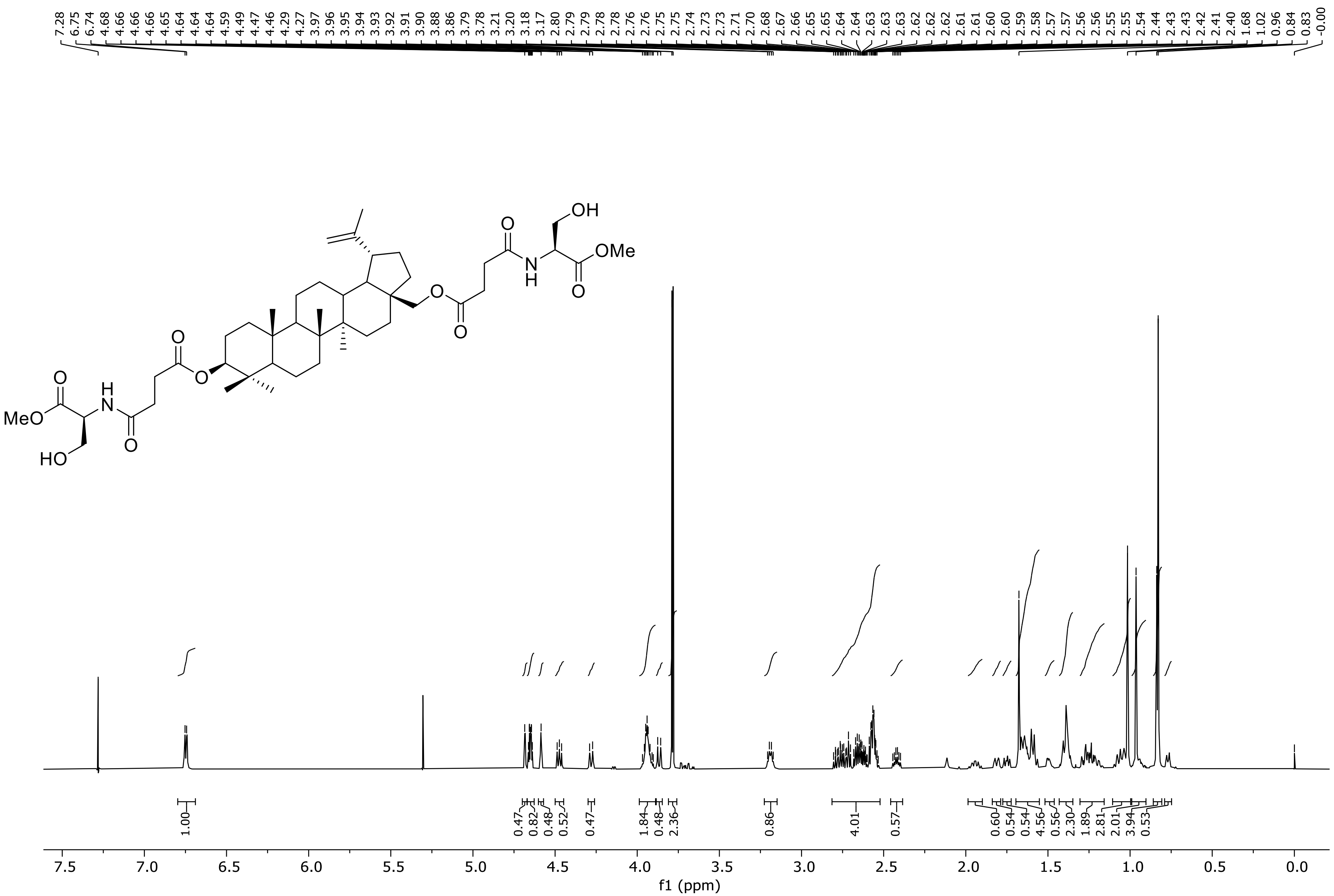

Figure S33. ^{1}H NMR spectrum of 3,28-*bis*[O-Suc-Ser(OMe)]-BN (**7d**); 600 MHz/$CDCl_3$/TMS; δ (ppm).

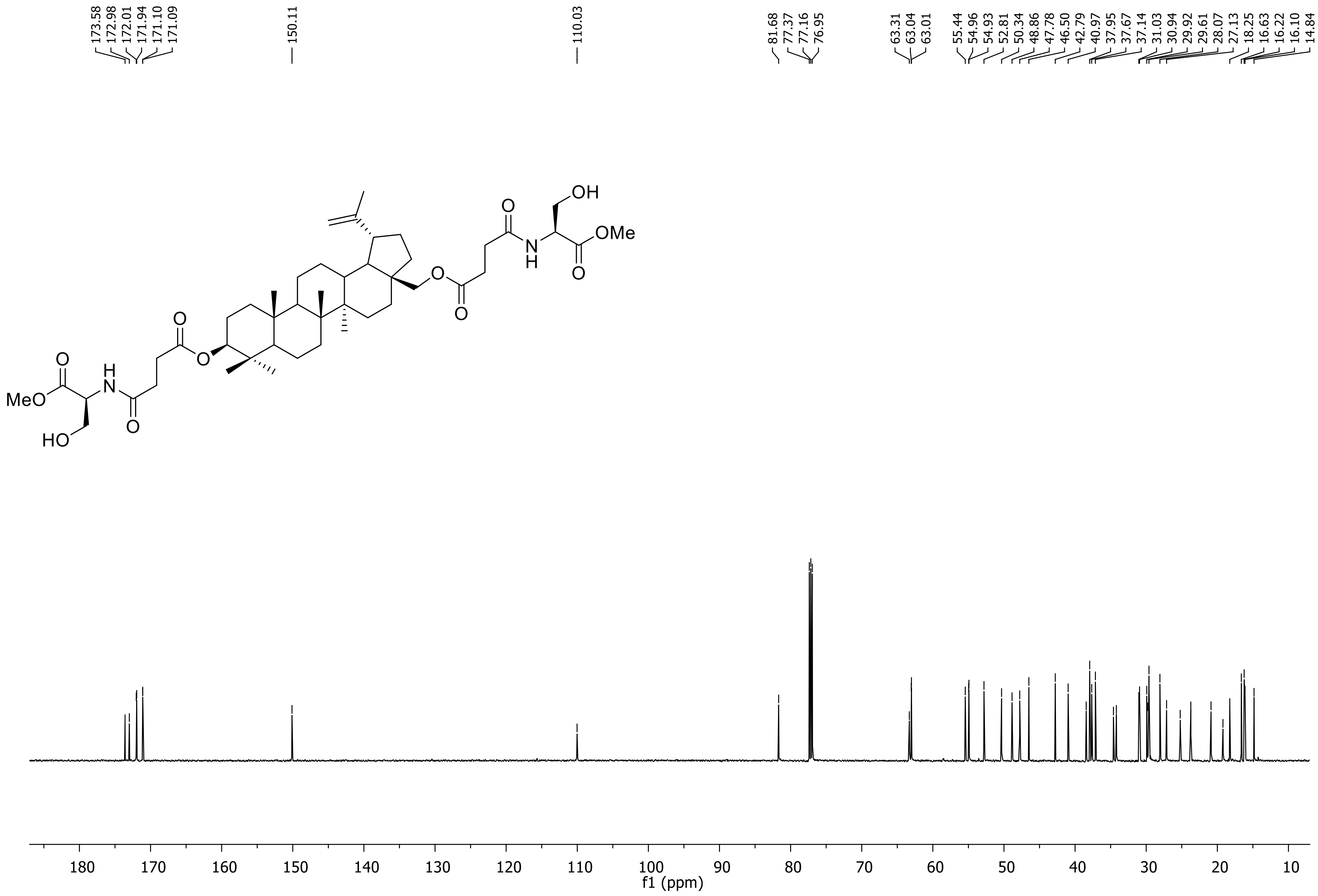

Figure S34. ^{13}C NMR spectrum of 3,28-*bis*[O-Suc-Ser(OMe)]-BN (**7d**); 150 MHz/$CDCl_3$/TMS; δ (ppm).

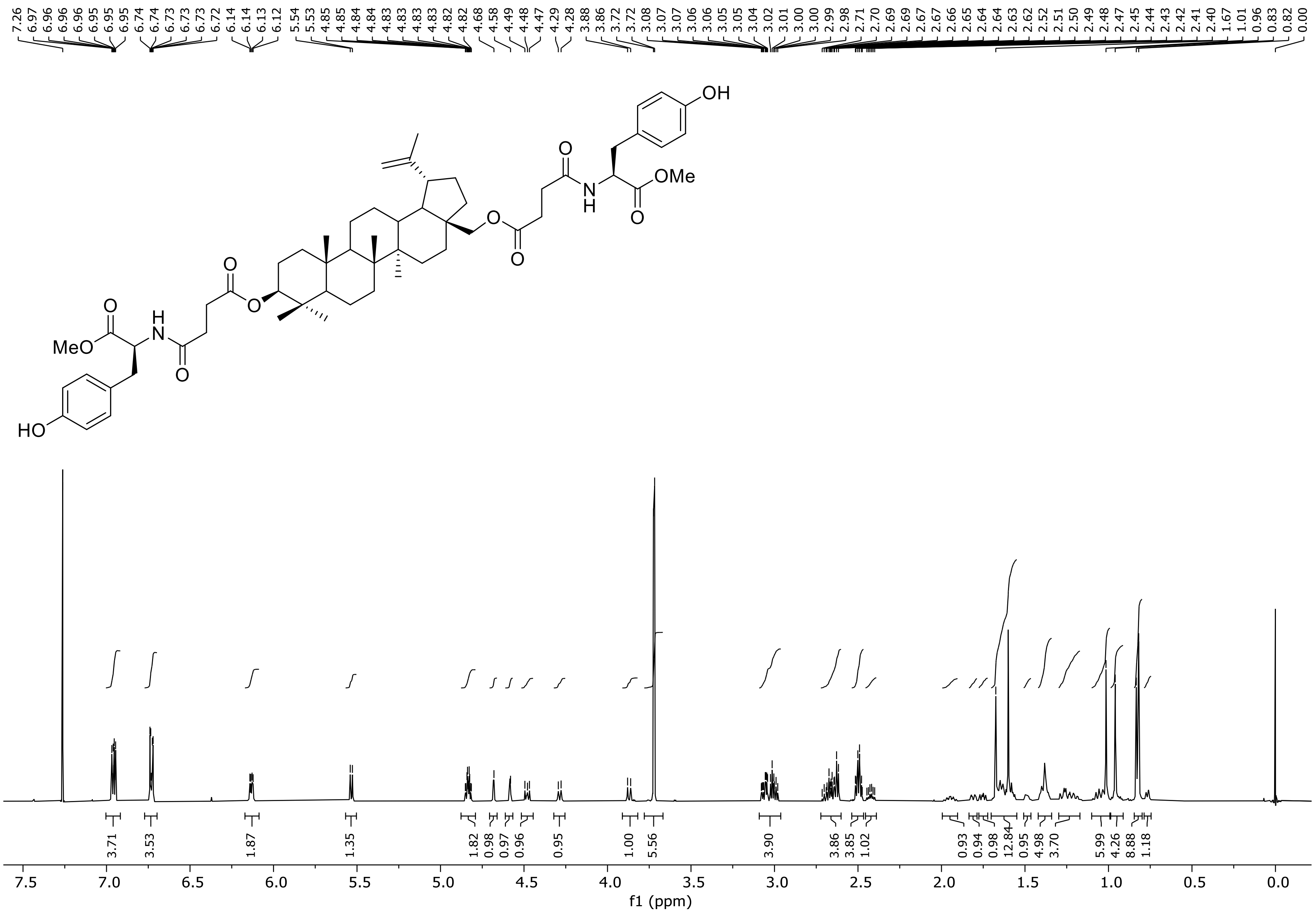

Figure S35. ^{1}H NMR spectrum of 3,28-*bis*[O-Suc-Tyr(OMe)]-BN (**7e**); 600 MHz/$CDCl_3$/TMS; *δ* (ppm).

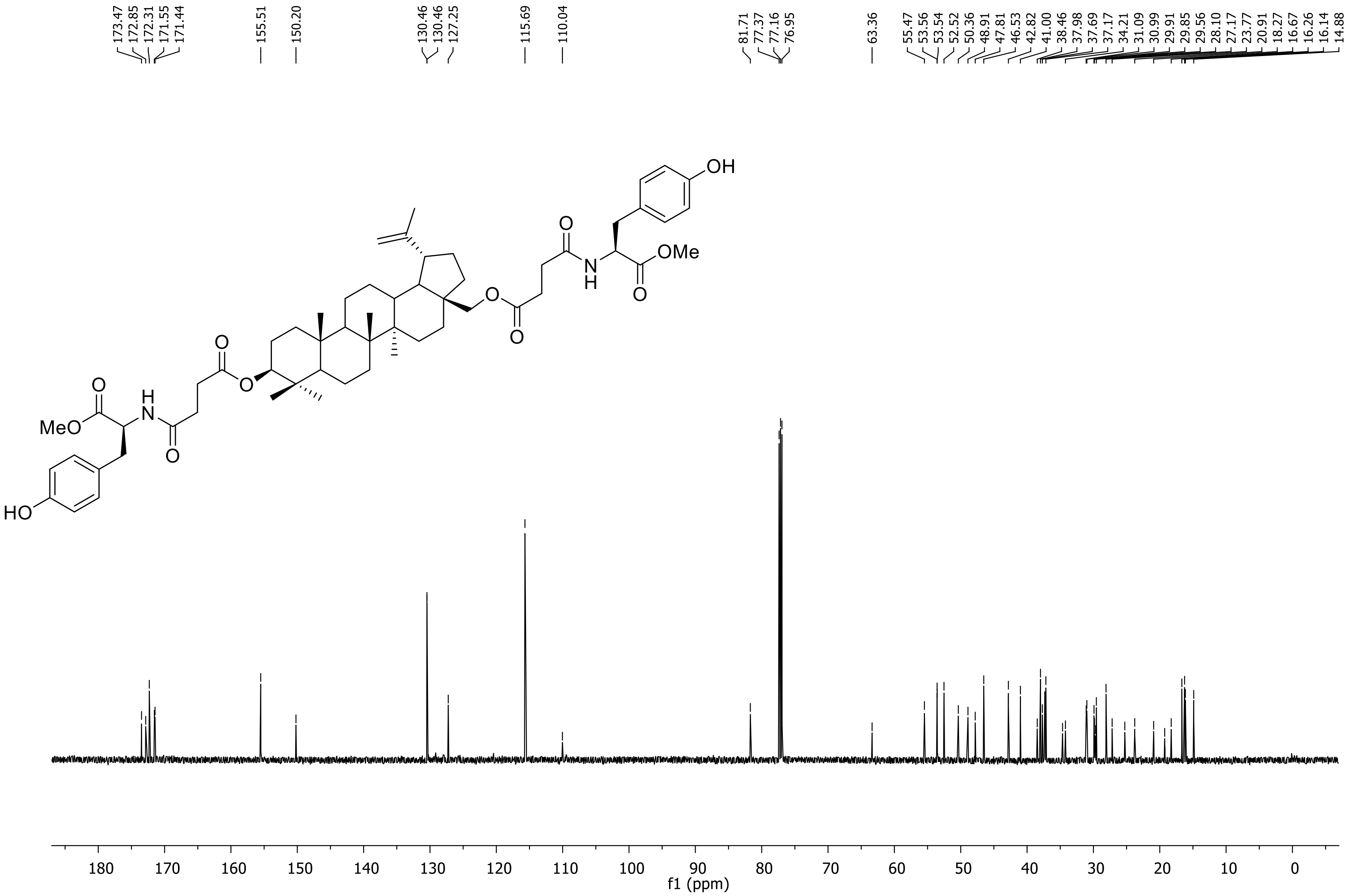

Figure S36. ^{13}C NMR spectrum of 3,28-*bis*[O-Suc-Tyr(OMe)]-BN (**7e**); 150 MHz/$CDCl_3$/TMS; δ (ppm).

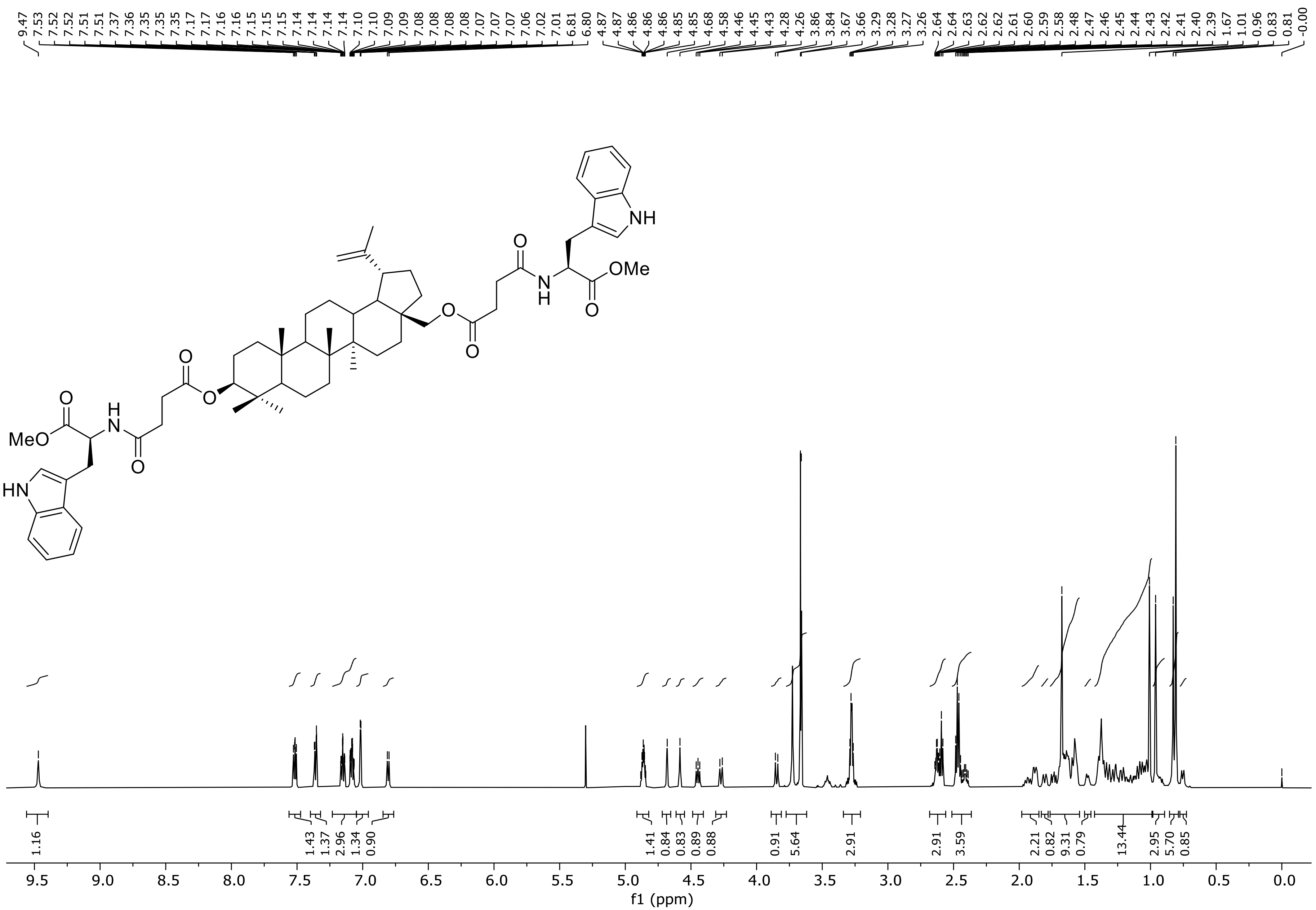

Figure S37. ^{1}H NMR spectrum of 3,28-bis[O-Suc-Trp(OMe)]-BN (**7f**); 600 MHz/$CDCl_3$/TMS; δ (ppm).

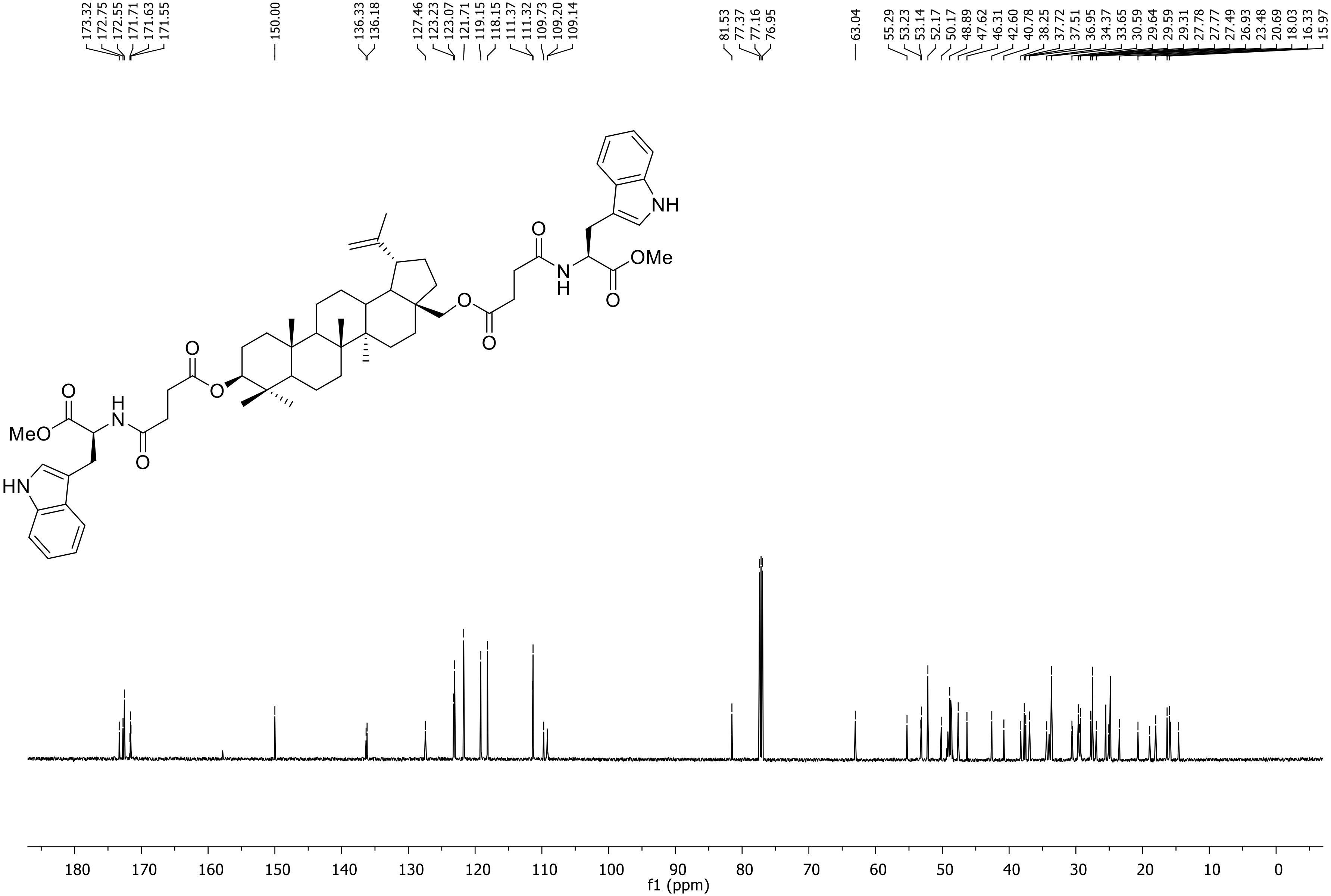

Figure S38. ^{13}C NMR spectrum of 3,28-bis[O-Suc-Trp(OMe)]-BN (**7f**); 150 MHz/$CDCl_3$/TMS; δ (ppm).

Table S1. One-way ANOVA summary table.

**Compound**	**HCT 116**		**MCF-7**		**NHDF**	
	**F**	**p**	**F**	**p**	**F**	**p**
**BN**	3.58	0.046	0.92	0.49	1.13	0.39
**3a**	3.45	0.05	7.31	0.005	1.59	0.25
**3b**	0.21	0.92	2.00	0.17	0.34	0.85
**3c**	0.30	0.87	1.83	0.20	0.81	0.55
**3d**	0.38	0.82	3.57	0.046	0.97	0.46
**3e**	33.02	0.00001	12.44	0.0007	31.07	0.00001
**3f**	8.48	0.003	31.49	0.00001	8.96	0.002
**3g**	0.94	0.48	0.28	0.89	0.41	0.80
**3h**	11.92	0.0008	7.50	0.004	62.62	0.0000005
**3i**	0.26	0.86	2.15	0.14	0.20	0.93
**3j**	0.36	0.83	16.18	0.0002	5.13	0.016
**5a**	2.85	0.08	2.05	0.16	0.38	0.81
**5b**	2.63	0.10	1.01	0.45	0.25	0.90
**7a**	3.55	0.048	15.83	0.0002	20.21	0.00009
**7b**	0.35	0.84	3.58	0.046	10.94	0.001
**7c**	0.46	0.76	4.49	0.025	13.64	0.00046
**7d**	52.92	0.000001	2.71	0.09	98.74	0.0000005
**7e**	8.74	0.003	1.66	0.23	2.55	0.10
**7f**	0.55	0.70	0.76	0.57	1.76	0.21

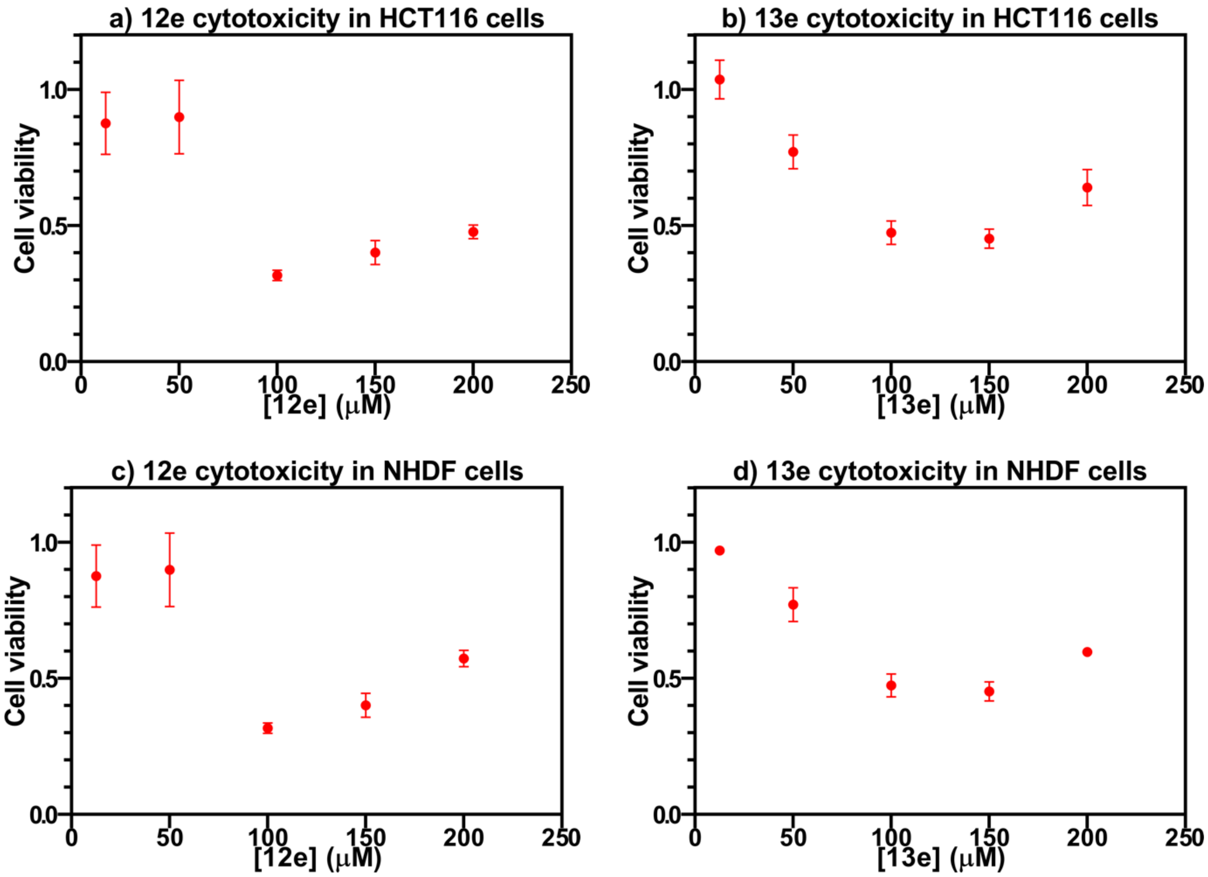

Figure S39. Effect of the tested BNAA hybrids (**12e, 13e**) on the cell viability of HCT 116 and NHDF cells. Cells were treated with different concentrations of BNAA hybrids for 24 h and then cell viability was measured by the CCK-8 assay. The results are provided as mean values with ±SD from at least three independent experiments. Cell viability was normalized to control (untreated) cells.

IC_{50} values were not calculated for the newly synthesized hybrids because the obtained concentration–response profiles did not meet the basic assumptions required for reliable nonlinear regression analysis. A classical IC_{50} determination assumes a monotonic concentration-dependent decrease in cell viability, preferably with clearly defined upper and lower plateaus. In contrast, several of the tested compounds showed non-monotonic profiles. In particular, cell viability decreased at intermediate concentrations, but then partially increased again at higher concentrations. This pattern was observed, for example, for compounds 12e and 13e, where the strongest reduction in viability occurred around 100–150 μM, whereas at 200 μM the viability was higher than expected for a standard cytotoxic dose–response relationship.

The observed profiles may reflect limited solubility, aggregation, compound precipitation, assay interference, or a non-classical biological response at higher concentrations. For this reason, the data are presented as cell viability values at individual tested concentrations rather than as fitted IC_{50} values. This approach avoids overinterpretation and provides a more transparent representation of the experimental results.
